# Supplementary material for: Among-site variability in the stochastic dynamics of East African coral reefs
Source: PeerJ. 2017 May 17;5:e3290. doi: 10.7717/peerj.3290 (PMC5437857; doi:10.7717/peerj.3290)
Supplement: Supplemental Information 1 [file peerj-05-3290-s001.pdf]

Among-site variability in the stochastic dynamics of East African coral reefs

Supporting material

Katherine A. Allen, John F. Bruno, Fiona Chong, Damian Clancy,

Tim R. McClanahan, Matthew Spencer, Kamila Żychaluk

March 20, 2017

A1 Data transformation

Proportional cover data were transformed to isometric log-ratio (ilr) coordinates (Egozcue et al., 2003). Let $\mathbf{z}_{i,j,t} = [z_{1,i,j,t}, z_{2,i,j,t}, z_{3,i,j,t}]^T$ denote a vector of observed proportional cover of coral ($z_{1,i,j,t}$), algae ($z_{2,i,j,t}$) and other ($z_{3,i,j,t}$) at site i, transect j, at time t (the T denotes transpose). Then the ilr transformation for our data is given by

$$\begin{aligned} &\text{ilr}\colon \mathbb{S}^3 \to \mathbb{R}^2, \\ &\mathbf{z}_{i,j,t} = [z_{1,i,j,t}, z_{2,i,j,t}, z_{3,i,j,t}]^T \mapsto \left[\frac{1}{\sqrt{2}}\log\left(\frac{z_{2,i,j,t}}{z_{1,i,j,t}}\right), \frac{2}{\sqrt{6}}\log\left(\frac{z_{3,i,j,t}}{\sqrt{z_{1,i,j,t}z_{2,i,j,t}}}\right)\right]^T, \end{aligned} \tag{A.1}$$

where $\mathbb{S}^3$ denotes the open 2-simplex in which three-part compositions lie. The first element of the transformed composition is proportional to the natural log of the ratio of algae to coral, and the second element is proportional to the natural log of the ratio of other to the geometric mean of algae and coral. The transformation can be thought of as stretching out the open 2-simplex (Figure A2(a)) so that it covers the whole of the real plane (Figure A2(b)).

As the domain of the transformation is the open simplex, which does not include compositions with zero parts, any observed zeros were replaced by half the smallest non-zero value recorded (0.0008) before transformation, and the other components rescaled accordingly. This is the simple replacement strategy described in Martín-Fernández et al. (2003), although more sophisticated approaches are possible. We denote the resulting transformed observations by $\mathbf{y}_{i,j,t} = [y_{1,i,j,t}, y_{2,i,j,t}]^T$.

A2 The model

For convenience, we reproduce the full model equations here:

$$
\begin{aligned}
\mathbf{x}_{i,t+1} &= \mathbf{a} + \boldsymbol{\alpha}_i + \mathbf{B}\mathbf{x}_{i,t} + \boldsymbol{\varepsilon}_{i,t}, \\
\boldsymbol{\alpha}_i &\sim \mathcal{N}(\mathbf{0}, \mathbf{Z}), \\
\boldsymbol{\varepsilon}_{i,t} &\sim \mathcal{N}(\mathbf{0}, \boldsymbol{\Sigma}), \\
\mathbf{y}_{i,j,t} &\sim t_2(\mathbf{x}_{i,t}, \mathbf{H}, \nu),
\end{aligned}
\tag{A.2}
$$

where $\mathbf{x}_{i,t}$ is the true transformed composition at site i, time t, $\mathbf{a}$ is a vector of among-site mean proportional changes evaluated at $\mathbf{x}_{i,t} = \mathbf{0}$, $\boldsymbol{\alpha}_i$ represents the amount by which these proportional changes for the ith site differ from the among-site mean, the 2×2 matrix $\mathbf{B}$ represents the effects of $\mathbf{x}_{i,t}$ on the proportional changes, $\boldsymbol{\varepsilon}_{i,t}$ represents random temporal variation,

$$
\mathbf{Z} = \begin{bmatrix} \zeta_{11} & \zeta_{12} \\ \zeta_{21} & \zeta_{22} \end{bmatrix}
$$

is the covariance matrix of the among-site term $\boldsymbol{\alpha}_i$ (note that throughout, a diagonal element such as ζ_{ii} of a covariance matrix represent the variance of the ith variable),

$$
\boldsymbol{\Sigma} = \begin{bmatrix} \sigma_{11} & \sigma_{12} \\ \sigma_{21} & \sigma_{22} \end{bmatrix}
$$

is the covariance matrix of the temporal variation, $\mathbf{y}_{i,j,t}$ is the observed log-ratio transformed cover in the jth transect of site i at time t,

$$\mathbf{H} = \begin{bmatrix} \eta_{11} & \eta_{12} \\ \eta_{21} & \eta_{22} \end{bmatrix}$$

is the scale matrix of the bivariate t distribution of the $\mathbf{y}_{i,j,t}$, and ν is the corresponding degrees of freedom.

A3 Describing measurement error and small-scale temporal variability

We initially considered using a bivariate normal distribution to describe the variability of observed transformed composition $\mathbf{y}_{i,j,t}$ around true composition $\mathbf{x}_{i,t}$, but preliminary analyses showed that a heavier-tailed distribution was needed. We therefore used the bivariate t distribution with location vector $\mathbf{x}_{i,t}$, scale matrix $\mathbf{H}$ and degrees of freedom ν, which for $\nu > 2$ has covariance matrix $\nu\mathbf{H}/(\nu-2)$ (Lange et al., 1989). Support for the choice of the t over the normal distribution was provided by expected predictive accuracy based on leave-one-out cross-validation (Vehtari et al., 2015), which was much higher for the bivariate t model than for the bivariate normal model (difference in leave-one-out cross-validation score 527, standard error 48).

A4 Visualizing model parameters

The effects of reef composition on short-term dynamics are most easily visualized by the back transformation from ilr coordinates to the simplex of the columns of the matrix $\mathbf{A} = \mathbf{B} - \mathbf{I}_2$, where $\mathbf{I}_k$ denotes the $k \times k$ identity matrix. The matrix $\mathbf{A}$ describes effects of transformed reef composition on year-to-year changes in transformed reef composition (Cooper et al., 2015). This is a better visualization than the back transformation of $\mathbf{B}$, because in the random walk case

(where there are no interesting composition effects), $\mathbf{A} = \mathbf{0}_2$ (the 2×2 matrix of zeros), and each column of the back-transformation of $\mathbf{A}$ represents a point at the origin of the simplex. In contrast, in the random walk case, each column of the back transformation of $\mathbf{B} = \mathbf{I}_2$ represents a point at a different location in the simplex. The first column $\mathbf{a}_1$ of $\mathbf{A}$ represents the effect of a unit increase in the first component of reef composition (proportional to log(algae/coral)) on year-to-year change in reef composition. For example, if the back-transformation of $\mathbf{a}_1$ lies to the left of the centre of the simplex (the origin, with equal proportions of coral, algae and other), but on the line of equal relative abundances of coral and other (the 1:1 coral-other isoproportion line), it indicates that high algal cover relative to coral tends to result in a decrease in algae relative to coral in the following year. Similarly, the second column $\mathbf{a}_2$ of $\mathbf{A}$ represents the effect of a unit increase in the second component of reef composition (proportional to log(other/geometric mean(algae,coral))) on year-to-year change in reef composition.

A5 Parameter estimation

Code for all analyses is available at `https://www.liverpool.ac.uk/~matts/kenya.zip`.

A5.1 Priors

For $\mathbf{Z}$ and $\boldsymbol{\Sigma}$, our priors were based on data from the Great Barrier Reef (Cooper et al., 2015). We inspected the sample covariance matrices for ilr-transformed year-to-year changes in composition, and among-site variation in mean composition, on 55 sites in the Great Barrier Reef, where observation error is thought to be fairly small (Cooper et al., 2015). We chose inverse Wishart priors (Gelman et al., 2003, p. 574) with 4 degrees of freedom (the smallest value for which the prior mean exists, giving a fairly uninformative prior). We chose identity scale matrices, because ellipses of unit Mahalanobis distance around the origin for the mean of this prior almost enclosed corresponding ellipses for the sample covariance matrices of both year-to-year changes and among-site mean composition, and strong correlations among

transformed components are neither assumed nor ruled out. Thus, this seems a plausible prior for Σ and $\mathbf{Z}$. In the absence of strong prior information, we used the same prior for $\mathbf{H}$.

For the degrees of freedom of measurement error, ν, we assumed a $U(2,30)$ distribution. The lower bound was dictated by the requirement that $\nu > 2$ for the covariance to exist, and the upper bound was chosen to be large enough that the resulting measurement error distribution was able to approach a multivariate normal if necessary. In practice, the posterior distribution of ν did not pile up against either of these bounds, indicating that the precise choice of prior was unlikely to matter.

We chose vague priors for the other parameters. We assumed independent $\mathcal{N}(0,10)$ priors on each element of $\mathbf{x}_{i,0}$ for each site i (where the subscript 0 denotes the first time point at which the site was observed). For each element of $\mathbf{a}$ and $\mathbf{B}$, we assumed independent $\mathcal{N}(0,100)$ priors.

A5.2 Monte Carlo simulation

We ran four Monte Carlo chains in parallel for 5000 iterations each, after a 5000-iteration warmup period. This took approximately two hours on a 64-bit Ubuntu 12.04 system with 4 3.2 GHz Intel Xeon cores and 16 GiB RAM. The potential scale reduction statistic, which takes the value 1 if all chains have converged to a common distribution, was 1.00 to two decimal places for all parameters, consistent with satisfactory convergence (Stan Development Team, 2015, pp. 414-415). Effective sample sizes, which measure the size of the sample from the posterior distribution after accounting for autocorrelation in the Monte Carlo chains (Stan Development Team, 2015, pp. 417-419), were at least 2839 for all parameters (most were much larger, with first quartile 12430 and median 17490). Inspection of trace plots did not reveal any obvious problems with sampling. In addition, we evaluated the model's performance in estimating known parameters. We generated 100 simulated data sets with identical structure to the real data, using posterior mean estimates for each parameter. We sampled the α_i, $\varepsilon_{i,t}$ and $\mathbf{y}_{i,j,t}$ from distributions defined by Equation A.2, and set the initial true transformed compositions at a given site to the sample means from all years and transects on that site in the real data. The estimates were reasonably close to the true values, and lay within the 95% HPD intervals in 89-99 out of 100

cases (Figure A3). Thus, while estimating state-space models from ecological time series data can be challenging (Auger-Méthé et al., 2015), performance appears adequate in this case, perhaps because we have many replicate transects from which to estimate measurement error and small-scale spatial variability, and most parameters are estimated using data across many sites.

A5.3 Model checking

We examined plots of Bayesian residuals (Gelman et al., 2003, p. 170) against predicted values of the two components of transformed reef composition. For the kth Monte Carlo iteration, the Bayesian residual for the jth transect on the ith site at time t is $\mathbf{y}_{i,j,t} - \mathbf{x}_{i,t} | \boldsymbol{\theta}_k$, where $\boldsymbol{\theta}_k$ denotes the estimated parameters in the kth iteration. If the model is performing well, there should be no obvious relationship between residuals and fitted values. We checked 16 randomly-chosen iterations, which did not reveal any major cause for concern (Figures A4, A5). However, no residuals for component 1 fell below an obvious diagonal line (Figure A4), which results from the treatment of observed zeros. Given the simple replacement strategy for zeros described in Section A1 and the definition of component 1 of the transformed composition in Equation A.1,

$$\begin{aligned} y_{1,i,j,t} &= \frac{1}{\sqrt{2}} \log\left(\frac{z_{2,i,j,t}}{z_{1,i,j,t}}\right) \\ &\geq \frac{1}{\sqrt{2}} \log\left(\frac{0.0008}{0.9984}\right) \quad = -5.0216. \end{aligned}$$

Thus the Bayesian residual for component 1 is constrained by

$$y_{1,i,j,t} - x_{1,i,t} | \boldsymbol{\theta}_k \geq -5.0216 - x_{1,i,t} | \boldsymbol{\theta}_k,$$

the orange line on Figure A4. Thus the assumption of a multivariate t distribution for individual transect deviations from true values (Equation A.2) cannot hold exactly. It might in future be worth attempting to develop a more mechanistic model of the process generating observed zeros, but we do not attempt this here because the majority of data are unaffected. Although a similar constraint exists on component 2, it did not appear to be important in practice, because there is no

obvious diagonal line of residuals on Figure A5.

Inspection of quantile-quantile plots and histograms of estimated skewness and kurtosis for 16 iterations did not indicate any major problems with the assumptions of multivariate normal distributions with zero mean, covariance matrices $\mathbf{Z}$ and Σ respectively for $\boldsymbol{\alpha}$ and ε, and a multivariate t distribution with zero location vector, scale matrix $\mathbf{H}$, for Bayesian residuals. Quantile-quantile plots used the natural log of a squared Mahalanobis-like distance/2 against natural log of quantiles of $\chi^2(2)$ for multivariate normal distributions, or against natural log of quantiles of $F(2, \nu)$ for multivariate t distributions (modified from Lange et al., 1989). We did not transform to asymptotically standard normal deviates because the degrees of freedom for the t distribution were small. We found it helpful to log transform both axes, particularly for the multivariate t distribution, for which some observations may have very large squared Mahalanobis-like distance. We obtained the p-values for several tests of multivariate normality of $\boldsymbol{\alpha}$ and ε: Royston's H (Royston, 1982), Henze-Zirkler's test (Henze and Zirkler, 1990), and Mardia's skewness and kurtosis (Mardia, 1970) using the MVN package in R (Korkmaz et al., 2014). There were more small p-values than expected (the distribution of p-values should be approximately uniform in the interval (0,1) if the data are normal) but that often is the case for very large samples, and does not indicate a major cause for concern.

A6 Long-term behaviour

Iterating Equation A.2 from a fixed initial transformed composition $\mathbf{x}_{i,0}$,

$$\mathbf{x}_{i,t} = \sum_{j=0}^{t-1} \mathbf{B}^j \mathbf{a} + \sum_{j=0}^{t-1} \mathbf{B}^j \boldsymbol{\alpha}_i + \mathbf{B}^t \mathbf{x}_0 + \sum_{j=0}^{t-1} \mathbf{B}^j \varepsilon_{i,t-1-j} \tag{A.3}$$

If all the eigenvalues of $\mathbf{B}$ lie inside the unit circle in the complex plane, the system will converge to a stationary distribution as $t \to \infty$ (e.g. Lütkepohl, 1993, p. 10). If the eigenvalues of $\mathbf{B}$ are complex, they will form a complex conjugate pair $\lambda = re^{\pm i\theta}$ (where r is the magnitude and θ is the argument), and there will be oscillations with period $2\pi/\theta$, whose amplitudes will change by

a factor of r each year (e.g. Otto and Day, 2007, p. 355).

The first term in Equation A.3 is deterministic, and converges to

$$\boldsymbol{\mu}^* = (\mathbf{I}_2 - \mathbf{B})^{-1}\mathbf{a} \tag{A.4}$$

(e.g. Lütkepohl, 1993, p. 10), which represents the among-site mean of stationary mean transformed composition. The third term is also deterministic, and converges to $\mathbf{0}$, so that initial conditions are forgotten.

The second term, representing among-site variation, has mean vector $\mathbf{0}$ by definition, and the covariance matrix of its limit is

$$\begin{aligned}\mathbf{Z}^* &= \mathrm{V}\left[(\mathbf{I}_2 - \mathbf{B})^{-1}\boldsymbol{\alpha}_i\right] \\ &= (\mathbf{I}_2 - \mathbf{B})^{-1}\mathrm{V}\left[\boldsymbol{\alpha}_i\right]\left((\mathbf{I}_2 - \mathbf{B})^{-1}\right)^T \\ &= (\mathbf{I}_2 - \mathbf{B})^{-1}\mathbf{Z}\left((\mathbf{I}_2 - \mathbf{B})^{-1}\right)^T,\end{aligned} \tag{A.5}$$

since $(\mathbf{I}_2 - \mathbf{B})^{-1}$ is a constant matrix and $\boldsymbol{\alpha}_i$ is a random vector. The covariance matrix $\mathbf{Z}^*$ represents the among-site variation in stationary mean transformed composition.

The fourth term represents the long-term effects of temporal variability. It has mean vector $\mathbf{0}$ by definition, and it can be shown that it has covariance matrix

$$\boldsymbol{\Sigma}^* = \mathrm{vec}^{-1}\left((\mathbf{I}_4 - \mathbf{B}\otimes\mathbf{B})^{-1}\mathrm{vec}\,(\boldsymbol{\Sigma})\right) \tag{A.6}$$

(e.g. Lütkepohl, 1993, p. 22), where the vec operator stacks the columns of a matrix, vec^{-1} unstacks them, and $\otimes$ is the Kronecker product. The covariance matrix $\boldsymbol{\Sigma}^*$ can be interpreted as the stationary covariance of transformed reef composition, conditional on the value of $\boldsymbol{\alpha}_i$. Since among-site variation and temporal variation were assumed independent, the unconditional stationary covariance is $\boldsymbol{\Sigma}^* + \mathbf{Z}^*$. Both the conditional and unconditional stationary distributions

are multivariate normal, since both $\varepsilon_{i,t}$ and $\boldsymbol{\alpha}_i$ were assumed multivariate normal. Thus the stationary distribution for a randomly-chosen site is the multivariate normal vector

$$\mathbf{x}^* \sim \mathscr{N}(\boldsymbol{\mu}^*, \boldsymbol{\Sigma}^* + \mathbf{Z}^*). \tag{A.7}$$

To find the long-term behaviour for a given site i, we condition on the value of $\boldsymbol{\alpha}_i$. Thus Equation A.4 is replaced by

$$\boldsymbol{\mu}_i^* = (\mathbf{I}_2 - \mathbf{B})^{-1}(\mathbf{a} + \boldsymbol{\alpha}_i),$$

and the stationary distribution is

$$\mathbf{x}_i^* \sim \mathscr{N}(\boldsymbol{\mu}_i^*, \boldsymbol{\Sigma}^*).$$

A7 How important is among-site variability?

From Equation A.7, the covariance matrix $\boldsymbol{\Sigma}^* + \mathbf{Z}^*$ of the stationary distribution for a randomly-chosen site contains contributions from both among- and within-site variability. To quantify the contributions from these two sources, we will use a statistic based on a ratio of generalized variances.

The generalized variance of a multivariate distribution is defined as the determinant of the covariance matrix (Wilks, 1932; Johnson and Wichern, 2007, section 3.4). In the specific case of a multivariate normal distribution, the generalized variance may be interpreted in terms of *ellipsoids of concentration*, defined as follows. Suppose a random vector $\mathbf{W}$ is distributed according to a p-dimensional normal distribution with mean vector $\boldsymbol{\mu}$ and covariance matrix $\mathbf{V}$. Then for any constant $k \geq 0$, the set $E_k = \left\{\mathbf{w} : (\mathbf{w} - \boldsymbol{\mu})^T \mathbf{V}^{-1} (\mathbf{w} - \boldsymbol{\mu}) = k\right\}$ consists of points $\mathbf{w}$ of constant probability density. In $p = 2$ dimensions, E_k is an ellipse, and may be referred to as a probability density contour. In $p > 2$ dimensions E_k is known as an ellipsoid of concentration of $\mathbf{V}$ about $\boldsymbol{\mu}$

(Kenward, 1979). Taking $k = 1$, the set E_1 is known as the unit ellipsoid of concentration. The volume within the unit ellipsoid E_1 may be used as a measure of the dispersion of the distribution, and is equal to $S_p\sqrt{|\mathbf{V}|}$, where S_p is the volume of the p-dimensional sphere of radius 1.

In the light of the above interpretation, we chose to measure the contribution of within-site variability to total variability using the quantity

$$\rho = \left(\frac{|\mathbf{\Sigma}^*|}{|\mathbf{\Sigma}^* + \mathbf{Z}^*|}\right)^{1/2}, \tag{A.8}$$

which is the ratio of volumes of two unit ellipsoids of concentration, the numerator corresponding to the stationary distribution in the absence of among-site variation, and the denominator to the full stationary distribution of transformed reef composition in the region. This ratio is undefined if $\mathbf{\Sigma}^* + \mathbf{Z}^*$ is not of full rank, but this does not occur in our application. From Minkowski's theorem (Mirsky, 1955, section 13.5) it follows that $|\mathbf{\Sigma}^*| + |\mathbf{Z}^*| \leq |\mathbf{\Sigma}^* + \mathbf{Z}^*|$, so that $0 \leq \rho \leq 1$. However, in general $|\mathbf{\Sigma}^*| + |\mathbf{Z}^*| \neq |\mathbf{\Sigma}^* + \mathbf{Z}^*|$, so that ρ cannot be simply interpreted as the proportion of total variability explained by within-site variation. Nevertheless, ρ provides an indication of how much of the total variability would remain if all among-site variability was removed.

Furthermore, ρ^2 is analogous to Wilks' Lambda (Wilks, 1932; Kenward, 1979), a likelihood-ratio test statistic often used in multivariate analysis of variance.

A8 Probability of low coral cover

For a given site i, the long-term probability $q_{\kappa,i}$ of coral cover less than or equal to κ is the integral of the multivariate normal stationary density for the site over the shaded area in Figure A37 (for $\kappa = 0.1$). This can be written as

$$q_{\kappa,i} = 1 - \int_{-\infty}^{u} P(X_2 \leq \gamma | X_1 = x_1) f_{X_1}(x_1)\,\mathrm{d}x_1, \tag{A.9}$$

where, using Equations A.1 and the constraint that the untransformed components of benthic composition must sum to 1,

$$u = \frac{1}{\sqrt{2}} \log\left(\frac{1}{\kappa} - 1\right)$$

is the largest value of the first ilr component x_1 for which it is possible to have coral cover less than or equal to κ,

$$\gamma = \frac{2}{\sqrt{6}} \log\left(\frac{1-\kappa\left(1+e^{\sqrt{2}x_1}\right)}{\kappa\sqrt{e^{\sqrt{2}x_1}}}\right)$$

is the value of the second ilr component x_2 for which coral cover is equal to κ, given the value of x_1, $P(X_2 \leq \gamma | X_1 = x_1)$ is the conditional marginal cumulative distribution of x_2, given the value of x_1, and $f_{X_1}(x_1)$ is the unconditional marginal density of the first ilr component x_1.

Since

$$\mathbf{X} = [X_1, X_2]^T \sim \mathcal{N}(\mu_i^*, \Sigma_i^*),$$

the unconditional marginal distribution of x_1 is

$$\mathcal{N}(\mu_{1,i}^*, \sqrt{\sigma_{11,i}^*}), \tag{A.10}$$

and the conditional marginal distribution of x_2 given x_1 is

$$\mathcal{N}\left(\mu_{2,i}^* + \frac{\sigma_{21,i}^*}{\sigma_{11,i}^*}(x_1 - \mu_{i,1}^*), \sigma_{22,i}^* - \frac{(\sigma_{21,i}^*)^2}{\sigma_{11,i}^*}\right) \tag{A.11}$$

(Gelman et al., 2003, p. 579). Then the integral in Equation A.9 can be approximated numerically using the `integrate()` function in R (R Core Team, 2015), which is based on routines in Piessens et al. (1983). The same approach can be used for q_κ for a randomly-chosen site, replacing the elements of $\boldsymbol{\mu}_i^*$ and Σ_i^* in Equations A.10 and A.11 with the corresponding

elements of $\boldsymbol{\mu}^*$ and Σ^*.

A9 Effects of among-site variability on the relationship between probability of low coral cover and sample mean coral cover

We generated simulated data sets with the same number of sites, number and spacing of observation times, and numbers of transects at each observation time, as the real data. Initial true transformed compositions at a given site were set to the sample means from all years and transects on that site in the real data. We used the posterior mean of each parameter from the real data to simulate these data, except that we set the among-site covariance matrix to $c\mathbf{Z}$, where $\mathbf{Z}$ was the posterior mean among-site covariance matrix from the real data, and c took nine equally-spaced values between 0 and 1. Thus $c = 0$ gives no among-site variability, and $c = 1$ gives as much among-site variability as was estimated from the real data. For each value of c, we calculated the sample mean coral cover in the simulated data for each site, and the long-term probability of coral cover ≤ 0.1 at each site as described in section A8. We then plotted these site-specific probabilities against the simulated sample mean coral cover for each site (Figure A39).

A10 Spline correlograms for spatial pattern in probability of low coral cover

We calculated a spline correlogram (Bjørnstad and Falck, 2001) for each set of $q_{0.1,i}$ in the 20000 Monte Carlo iterations, using the `spline.correlog()` function in the R package `ncf` version 1.15. We constructed a 95% highest-density envelope (Hyndman, 1996) for the resulting set of correlograms using the R package `hdrcde` version 3.1.

A11 Which model parameters have the largest effects on the probability of low coral cover?

For a given threshold κ, we can calculate (by numerical integration) the probability $q_\kappa = P(\text{coral cover} \leq \kappa)$, for a composition drawn from the stationary distribution on a site chosen at random from the region. The probability q_κ is a function of 12 parameters: all four elements of $\mathbf{B}$; both elements of $\mathbf{a}$; elements σ_{11}, σ_{21} and σ_{22} of Σ; and elements ζ_{11}, ζ_{21} and ζ_{22} of $\mathbf{Z}$. Note that because Σ and $\mathbf{Z}$ are covariance matrices, they must be symmetric, and so σ_{12} and ζ_{12} are not free parameters. These 12 parameters can be thought of as the coordinates of a point in $\mathbb{R}^{12}$. The steepest reduction in q_κ as we move through $\mathbb{R}^{12}$ is achieved by moving in the direction of $-\nabla q_\kappa$, where ∇q_κ is the gradient vector $[\partial q_\kappa / \partial b_{11}, \ldots, \partial q_\kappa / \partial \zeta_{22}]^T$ (Riley et al., 2002, p. 355).

To understand the effects of each parameter, note that the probability q_κ depends on these parameters only through $\boldsymbol{\mu}^*$, Σ^* and $\mathbf{Z}^*$. Thus, for any parameter matrix Θ, using the chain rule for matrix derivatives,

$$Dq_\kappa(\Theta) = Dq_\kappa(\boldsymbol{\mu}^*) D\boldsymbol{\mu}^*(\Theta) + Dq_\kappa(\Sigma^*) D\Sigma^*(\Theta) + Dq_\kappa(\mathbf{Z}^*) D\mathbf{Z}^*(\Theta),$$

where $D\mathbf{E}(\mathbf{X})$ denotes the matrix derivative of $\mathbf{E}$ with respect to $\mathbf{X}$ (Magnus and Neudecker, 2007, p. 108). This allows us to break up the effects of a parameter into its effects via the stationary mean and stationary within- and among-site covariances. In each term, the first factor ($Dq_\kappa(\boldsymbol{\mu}^*)$, $Dq_\kappa(\Sigma^*)$ or $D\Sigma^*(\Theta)$) can only be found numerically. The non-zero second factors are

$$
\begin{aligned}
D\boldsymbol{\mu}^*(\mathbf{B}) &= (\mathbf{a}^T \otimes \mathbf{I}_2)\left[\left((\mathbf{I}_2-\mathbf{B})^{-1}\right)^T \otimes (\mathbf{I}_2-\mathbf{B})^{-1}\right], \\
D\Sigma^*(\mathbf{B}) &= \mathbf{F}\left[(\mathrm{vec}\Sigma)^T \otimes \mathbf{I}_4\right]\left[\left((\mathbf{I}_4-\mathbf{B}\otimes\mathbf{B})^{-1}\right)^T \otimes (\mathbf{I}_4-\mathbf{B}\otimes\mathbf{B})^{-1}\right] \\
&\qquad (\mathbf{I}_2\otimes\mathbf{K}_4\otimes\mathbf{I}_2)(\mathbf{I}_4\otimes\mathrm{vec}\mathbf{B}+\mathrm{vec}\mathbf{B}\otimes\mathbf{I}_4), \\
D\mathbf{Z}^*(\mathbf{B}) &= \mathbf{F}\left[(\mathrm{vec}\mathbf{Z})^T\otimes\mathbf{I}_4\right](\mathbf{I}_2\otimes\mathbf{K}_4\otimes\mathbf{I}_2)\left[\mathbf{I}_4\otimes\mathrm{vec}(\mathbf{I}_2-\mathbf{B})^{-1}+\mathrm{vec}(\mathbf{I}_2-\mathbf{B})^{-1}\otimes\mathbf{I}_4\right] \\
&\qquad \left[\left((\mathbf{I}_2-\mathbf{B})^{-1}\right)^T\otimes(\mathbf{I}_2-\mathbf{B})^{-1}\right], \\
D\boldsymbol{\mu}^*(\mathbf{a}) &= (\mathbf{I}_2-\mathbf{B})^{-1}, \\
D\Sigma^*(\Sigma) &= \mathbf{F}(\mathbf{I}_4-\mathbf{B}\otimes\mathbf{B})^{-1}\mathbf{G}, \\
D\mathbf{Z}^*(\mathbf{Z}) &= \mathbf{F}\left[(\mathbf{I}_2-\mathbf{B})^{-1}\otimes(\mathbf{I}_2-\mathbf{B})^{-1}\right]\mathbf{G},
\end{aligned}
\tag{A.12}
$$

where $\mathbf{K}_4$ is the 4×4 commutation matrix (Magnus and Neudecker, 2007, p. 54),

$$
\mathbf{F} = \begin{bmatrix} 1 & 0 & 0 & 0 \\ 0 & 1 & 0 & 0 \\ 0 & 0 & 0 & 1 \end{bmatrix},
$$

and

$$
\mathbf{G} = \begin{bmatrix} 1 & 0 & 0 \\ 0 & 1 & 0 \\ 0 & 1 & 0 \\ 0 & 0 & 1 \end{bmatrix}.
$$

A12 Elasticity of probability of low coral cover

The derivatives in section A11 measure the rate of change of the probability of low coral cover, q_κ, with respect to absolute changes in parameters. However, because parameters may differ in magnitude, it is also of interest to measure the rate of relative change of q_κ with respect to relative

change in each parameter, in other words the elasticity of q_κ with respect to the parameter. The usual definition of the elasticity $\mathrm{El}_\theta(q_\kappa(\theta))$ of q_κ with respect to a parameter θ is

$$\begin{aligned}\mathrm{El}_\theta(q_\kappa(\theta)) &= \lim_{\Delta\theta\to 0}\frac{(\Delta q_\kappa)/q_\kappa(\theta)}{(\Delta\theta)/\theta}\\ &= \lim_{\Delta\theta\to 0}\frac{\theta}{q_\kappa(\theta)}\frac{q_\kappa(\theta+\Delta\theta)-q_\kappa(\theta)}{\Delta\theta}\\ &= \frac{\theta}{q_\kappa(\theta)}q'_\kappa(\theta)\end{aligned} \tag{A.13}$$

(Nievergelt, 1983) for $\theta \neq 0$ (typically, $\theta > 0$) and $q_\kappa(\theta) \neq 0$. We need to slightly change the usual definition because in our model there are three parameters (b_{12}, b_{21} and ζ_{12}) for which both positive and negative values occur in the sample from the posterior. First, although the first line of Equation A.13 is not defined at $\theta = 0$, the continuous function on the second line is defined (and has the value 0) at $\theta = 0$, agrees with the first line at all points other than $\theta = 0$, and tends to 0 as $\theta \to 0$. It therefore fills the gap in a natural way. Second, we would like the elasticity to be positive when the derivative of q_κ with respect to θ is positive, even when θ is negative. We therefore calculated elasticities as

$$\mathrm{El}_\theta(q_\kappa(\theta)) = \frac{|\theta|}{q_\kappa(\theta)}q'_\kappa(\theta).$$

A13 How informative is a snapshot about long-term site properties?

Denote the true state of a randomly-chosen site at a given time by $\mathbf{x}$, and the corresponding stationary mean for that site by $\boldsymbol{\mu}^*$. Under the model of Equation A.2, $\boldsymbol{\mu}^*$ has covariance matrix $\mathbf{Z}^*$ (Equation A.5). Write the true state as $\mathbf{x} = \boldsymbol{\mu}^* + \boldsymbol{\Delta}$, where $\boldsymbol{\Delta}$ is the deviation from the stationary mean, which has covariance matrix $\boldsymbol{\Sigma}^*$ (Equation A.6). The correlation ρ_k between the kth component x_k of $\mathbf{x}$ and the corresponding component μ_k^* of $\boldsymbol{\mu}^*$ is an obvious way to measure how informative the snapshot will be for this component. This is

$$
\begin{aligned}
\rho_k &= \frac{\operatorname{cov}(\mu_k^* + \Delta_k, \mu_k^*)}{\sqrt{V[\mu_k^* + \Delta_k]V[\mu_k^*]}} \\
&= \frac{V[\mu_k^*] + \operatorname{cov}(\mu_k^*, \Delta_k)}{\sqrt{V[\mu_k^* + \Delta_k]V[\mu_k^*]}} \\
&= \frac{V[\mu_k^*]}{\sqrt{(V[\mu_k^*] + V[\Delta_k])V[\mu_k^*]}} \qquad \text{(because } \alpha \text{ and } \varepsilon \text{ assumed independent)} \\
&= \left(\frac{\zeta_{kk}^*}{\zeta_{kk}^* + \sigma_{kk}^*} \right)^{1/2},
\end{aligned}
$$

where ζ_{kk}^* is the kth diagonal element of $\mathbf{Z}^*$, and σ_{kk}^* is the kth diagonal element of Σ^*. If ρ_k is far from zero, a snapshot will be a reliable guide to the long-term value of the kth component of transformed reef composition. On the other hand, if ρ_k is close to zero, a snapshot will be unreliable. Thus ρ_k measures the extent to which conservation and management decisions could be based on observations at a single time point. We computed both ρ_1 which tells us how much we could learn about the log of the ratio of algae to coral and ρ_2, which tells us how much we could learn about the log of the ratio of other to the geometric mean of coral and algae.

A14 Dynamics

Consistent with the patterns suggesting negative feedbacks that will tend to maintain fairly stable reef composition, every set of sampled parameters led to a stationary distribution (Figure A38: all sampled eigenvalues of $\mathbf{B}$ fell inside the unit circle in the complex plane, with maximum magnitude 0.84). In 27% of iterations, there was evidence for oscillations on the approach to the stationary distribution, because the eigenvalues were complex. In such cases, the oscillations had a long period (posterior mean 113 years, 95% HPD interval $(21, 284)$ years), but their amplitude more than halved within three years because the magnitudes of the eigenvalues involved were small (original posterior mean magnitude of complex eigenvalues 0.59, 95% credible interval $(0.51, 0.67)$, cubed posterior mean magnitude 0.21, 95% HPD interval $(0.13, 0.30)$). The distribution of eigenvalues was very different from that of the Great Barrier Reef (Cooper et al., 2015, Appendix A.10), where the largest eigenvalue lay close to the point beyond which the

stationary distribution would not exist (bootstrap mean magnitude 0.95), and there was no evidence for oscillations (no bootstrap replicates had complex eigenvalues). However, a different estimation method was used in Cooper et al. (2015), so the eigenvalues may not be directly comparable.

A15 Probability of low coral cover: signs of derivatives

Here, we explain the signs of the derivatives of the probability of low coral cover with respect to each parameter. We concentrate on coral cover threshold 0.1. The overall stationary mean $\boldsymbol{\mu}^*$ lies in the region where coral cover is greater than 0.1 for all iterations (Figure A37, black circle, shows a point estimate for $\boldsymbol{\mu}^*$, based on the stationary means of **a** and **B**). The shaded region of Figure A37 has coral cover ≤ 0.1. Because of the shape of the boundary of the shaded region, either increasing μ_1^* (increasing the ratio of algae to coral) or increasing μ_2^* (increasing the ratio of other to the geometric mean of coral and algae) will move the stationary mean closer to this region. Also, since the stationary mean lies outside the region of interest, increasing the variability in the stationary distribution by increasing the elements of $\boldsymbol{\Sigma}^*$ or $\mathbf{Z}^*$ will increase the probability of falling in the region of interest. Hence the derivatives of $q_{0.1}$ with respect to $\boldsymbol{\mu}^*$, $\boldsymbol{\Sigma}^*$, $\mathbf{Z}^*$ contain only positive elements.

It is then intuitively obvious that the derivatives of $q_{0.1}$ with respect to $\boldsymbol{\Sigma}$ and $\mathbf{Z}$ will contain only positive elements. Increasing the amount of year-to-year temporal variability or among-site variability will increase the variability in the stationary distribution, and hence the long-term probability of coral cover less than or equal to 0.1.

The signs of the derivatives of $q_{0.1}$ with respect to **a** are also easy to understand. The components a_1, a_2 represent the rates of increase of x_1 and x_2 respectively, so we would expect that increasing either of them will increase the corresponding component of the stationary mean. Thus the derivatives of $\boldsymbol{\mu}^*$ with respect to **a** will be positive, and from Figure A37, increasing either component of $\boldsymbol{\mu}^*$ will increase the probability of coral cover ≤ 0.1.

The derivatives of $q_{0.1}$ with respect to $\mathbf{B}$ are a little harder to understand. They are (predominantly) negative with respect to b_{11} and b_{21}, but positive with respect to b_{12} and b_{22}. Since $\mathbf{B}$ affects both the stationary mean (Equation A.4) and the stationary covariance, which is the sum of $\mathbf{\Sigma}^*$ (Equation A.6) and $\mathbf{Z}^*$ (Equation A.5), all of these effects could be important. However, in 93% of iterations,

$$|Dq_{0.1}(\boldsymbol{\mu}*)D\boldsymbol{\mu}^*(\mathbf{B})| \succ |Dq_{0.1}(\mathbf{\Sigma}^*)D\mathbf{\Sigma}^*(\mathbf{B}) + Dq_{0.1}(\mathbf{Z}^*)D\mathbf{Z}^*(\mathbf{B})|,$$

where $\succ$ is an elementwise inequality, and $|\mathbf{D}|$ indicates the elementwise magnitude, such that for two matrices $\mathbf{D}$ and $\mathbf{E}$ with the same dimensions, $|\mathbf{D}| \succ |\mathbf{E}|$ if and only if the magnitude of every d_{ij} is greater than the magnitude of the corresponding e_{ij}. In other words, in almost all iterations, the sign of the effect of $\mathbf{B}$ on $q_{0.1}$ via $\boldsymbol{\mu}^*$ determines the sign of the overall effect of $\mathbf{B}$ on $q_{0.1}$. We therefore concentrate on understanding how $\mathbf{B}$ affects $\boldsymbol{\mu}^*$.

To understand the signs of the effects of b_{11} and b_{22} on $\boldsymbol{\mu}^*$, consider the one-dimensional deterministic analogue

$$x_{t+1} = a + bx_t.$$

Iterating this gives

$$x_t = a\left(1 + b + b^2 + \ldots + b^{t-1}\right) + b^t x_0.$$

For $0 < b < 1$, the term $b^t x_0 \to 0$ as $t \to \infty$. Then the derivative of x_∞ with respect to b has the same sign as a. In our system, $a_1 < 0$ and $a_2 > 0$, so we expect the signs of derivatives of $\boldsymbol{\mu}^*$ with respect to b_{11} to be negative, and the signs of derivatives of $\boldsymbol{\mu}^*$ with respect to b_{22} to be positive.

To understand the signs of the effects of b_{12} and b_{21} on $\boldsymbol{\mu}^*$, recall that b_{12} is the effect of component 2 (which typically takes positive values) on component 1, and b_{21} is the effect of component 1 (which typically takes negative values) on component 2. If, as in our system, b_{12} and b_{21} are both positive, and the system is linear, we would expect that the signs of their effects

on $\boldsymbol{\mu}^*$ will be the same as the signs of components 2 and 1 respectively.

Then, by the graphical argument above (Figure A37), we expect the signs of the derivatives of $q_{0.1}$ with respect to b_{11}, b_{21}, b_{12} and b_{22} to be $-$,$-$,$+$,$+$ respectively.

A16 Probability of low coral cover: rank order, other thresholds and elasticities

For threshold 0.05, the signs of the effects of b_{11} and b_{21} were not clearly negative. The four most important parameters were (in descending order: Figure A43) ζ_{21}, ζ_{22}, b_{22} and b_{12} (the same four as for threshold 0.1, but in a different order). For threshold 0.2, the signs were as for threshold 0.1, but the four most important parameters were (in descending order) b_{22}, b_{21}, b_{12} and ζ_{21} (with ζ_{22} now in fifth place: Figure A45). Thus, while the details depend to some extent on the threshold, the overall conclusion that both internal dynamics and among-site variability are the most important factors affecting the probability of low coral cover is robust.

The effects of within-site temporal variability on the probability of low coral cover were always relatively unimportant (threshold 0.1, Figure A41, three of the last four positions in the ranked list; threshold 0.05, Figure A43, three of the last five positions; threshold 0.20, Figure A45, last three positions).

For elasticities, the four most important parameters (in descending order) for threshold 0.1 were b_{22}, a_1, a_2 and ζ_{11} (Figure A46). The rank order of importance was similar for thresholds 0.05 (four most important parameters b_{22}, a_1, ζ_{11} and a_2, Figure A47) and 0.2 (four most important parameters b_{22}, a_1, a_2 and β_{11}, Figure A48). In all cases, elasticities were higher for elements of the among-site covariance matrix $\mathbf{Z}$ than for the corresponding elements of the within-site temporal variability covariance matrix $\boldsymbol{\Sigma}$, again supporting the argument that among-site variability is more important than within-site temporal variability.

References

Auger-Méthé, M., Field, C., Albertsen, C. M., Derocher, A. E., Lewis, M. A., Jonsen, I. D., and Mills Flemming, J. (2015). State-space models' dirty little secrets: even simple linear Gaussian models can have estimation problems. *unpublished*, arXiv:1508.04325v1.

Bjørnstad, O. N. and Falck, W. (2001). Nonparametric spatial covariance functions: Estimation and testing. *Environmental and Ecological Statistics*, 8:53–70.

Cooper, J. K., Spencer, M., and Bruno, J. F. (2015). Stochastic dynamics of a warmer Great Barrier Reef. *Ecology*, 96:1802–1811.

Egozcue, J. J., Pawlowsky-Glahn, V., Mateu-Figueras, G., and Barceló-Vidal, C. (2003). Isometric logratio transformations for compositional data analysis. *Mathematical Geology*, 35(3):279–300.

Gelman, A., Carlin, J. B., Stern, H. S., and Rubin, D. B. (2003). *Bayesian Data Analysis*. Chapman and Hall/CRC, Boca Raton, second edition.

Henze, N. and Zirkler, B. (1990). A class of invariant consistent tests for multivariate normality. *Communications in Statistics - Theory and Methods*, 19:3595–3617.

Hyndman, R. J. (1996). Computing and graphing highest density regions. *The American Statistician*, 50(2):120–126.

Johnson, R. A. and Wichern, D. W. (2007). *Applied multivariate statistical analysis*. Pearson, 6th edition.

Kenward, M. G. (1979). An intuitive approach to the MANOVA test criteria. *Journal of the Royal Statistical Society Series D*, 28(3):193–198.

Korkmaz, S., Goksuluk, D., and Zararsiz, G. (2014). MVN: An R package for assessing multivariate normality. *The R Journal*, 6:151–162.

Lange, K. L., Little, R. J. A., and Taylor, J. M. G. (1989). Robust statistical modeling using the t distribution. *Journal of the American Statistical Association*, 84:881–896.

Lütkepohl, H. (1993). *Introduction to multiple time series analysis*. Springer-Verlag, Berlin, 2nd edition.

Magnus, J. R. and Neudecker, H. (2007). *Matrix differential calculus with applications in statistics and econometrics*. John Wiley & Sons, Chichester, third edition.

Mardia, K. V. (1970). Measures of multivariate skewnees and kurtosis with applications. *Biometrika*, 57:519–530.

Martín-Fernández, J. A., Barceló-Vidal, C., and Pawlowsky-Glahn, V. (2003). Dealing with zeros and missing values in compositional data sets using nonparametric imputation. *Mathematical Geology*, 35(3):253–278.

Mirsky, L. (1955). *An introduction to linear algebra*. Oxford University Press, Oxford.

Nievergelt, Y. (1983). The concept of elasticity in economics. *SIAM Review*, 25:261–265.

Otto, S. P. and Day, T. (2007). *A biologist's guide to mathematical modeling in ecology and evolution*. Princeton University Press, Princeton, New Jersey.

Piessens, R., de Doncker-Kapenga, E., Überhuber, C. W., and Kahaner, D. (1983). *QUADPACK: a subroutine package for automatic integration*. Springer-Verlag, Berlin.

R Core Team (2015). *R: A Language and Environment for Statistical Computing*. R Foundation for Statistical Computing, Vienna, Austria.

Riley, K. F., Hobson, M. P., and Bence, S. J. (2002). *Mathematical methods for physics and engineering*. Cambridge University Press, Cambridge, second edition.

Royston, J. (1982). An extension of Shapiro and Wilk's W test for normality to large samples. *Applied Statistics*, 31:115–124.

Stan Development Team (2015). *Stan Modeling Language Users Guide and Reference Manual, Version 2.7.0*.

Vehtari, A., Gelman, A., and Gabry, J. (2015). Efficient implementation of leave-one-out cross-validation and WAIC for evaluating fitted Bayesian models. *unpublished*, arXiv:1507.04544v1.

Wilks, S. S. (1932). Certain generalizations in the analysis of variance. *Biometrika*, 24:471–494.

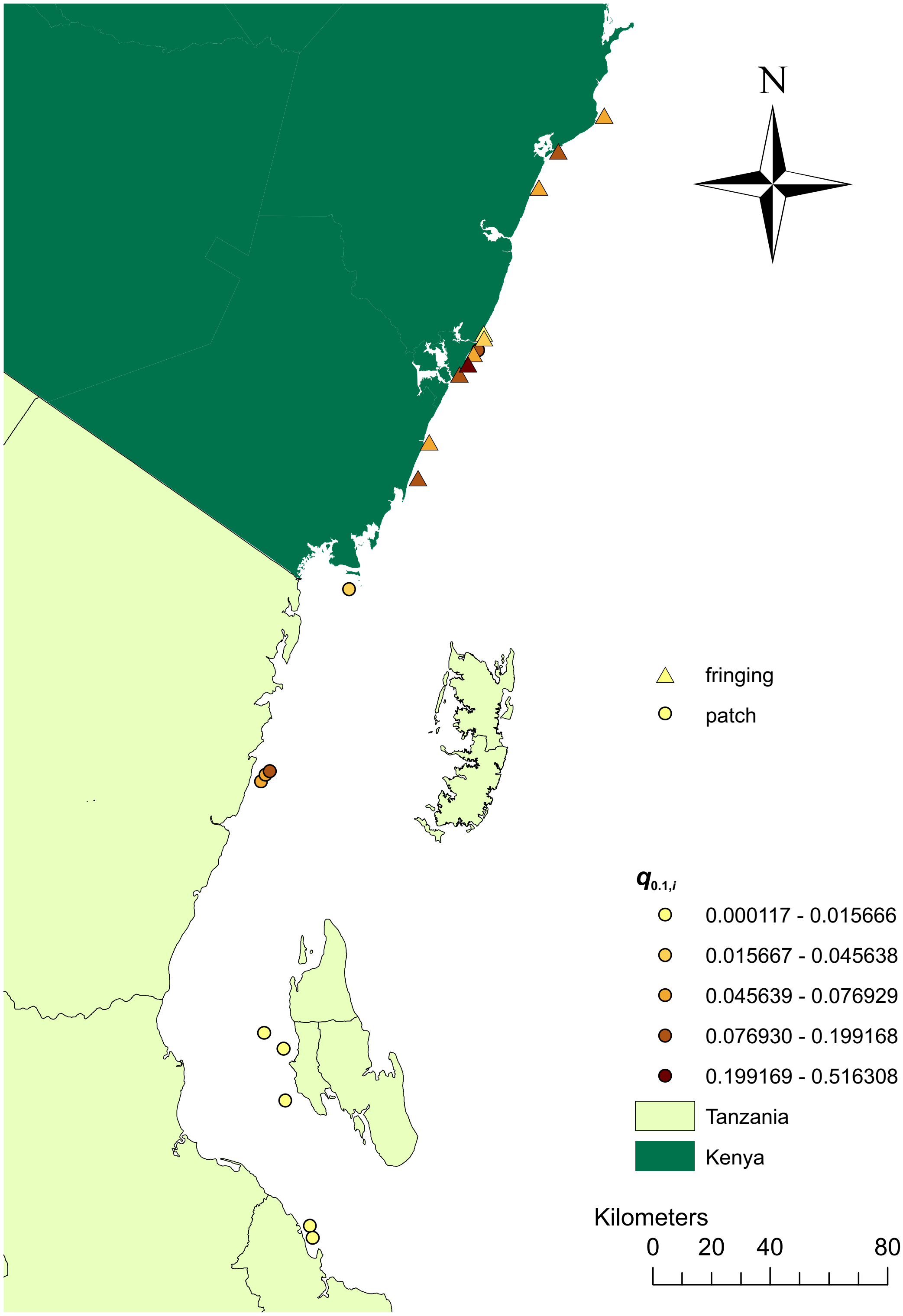

Figure A1: Map of study sites, showing fringing reefs (triangles) and patch reefs (circles), shaded by the site-specific long-term probability $q_{0.1,i}$ of coral cover ≤ 0.1 (for reefs with one site) or the mean of site-specific probabilities (for reefs with two sites).

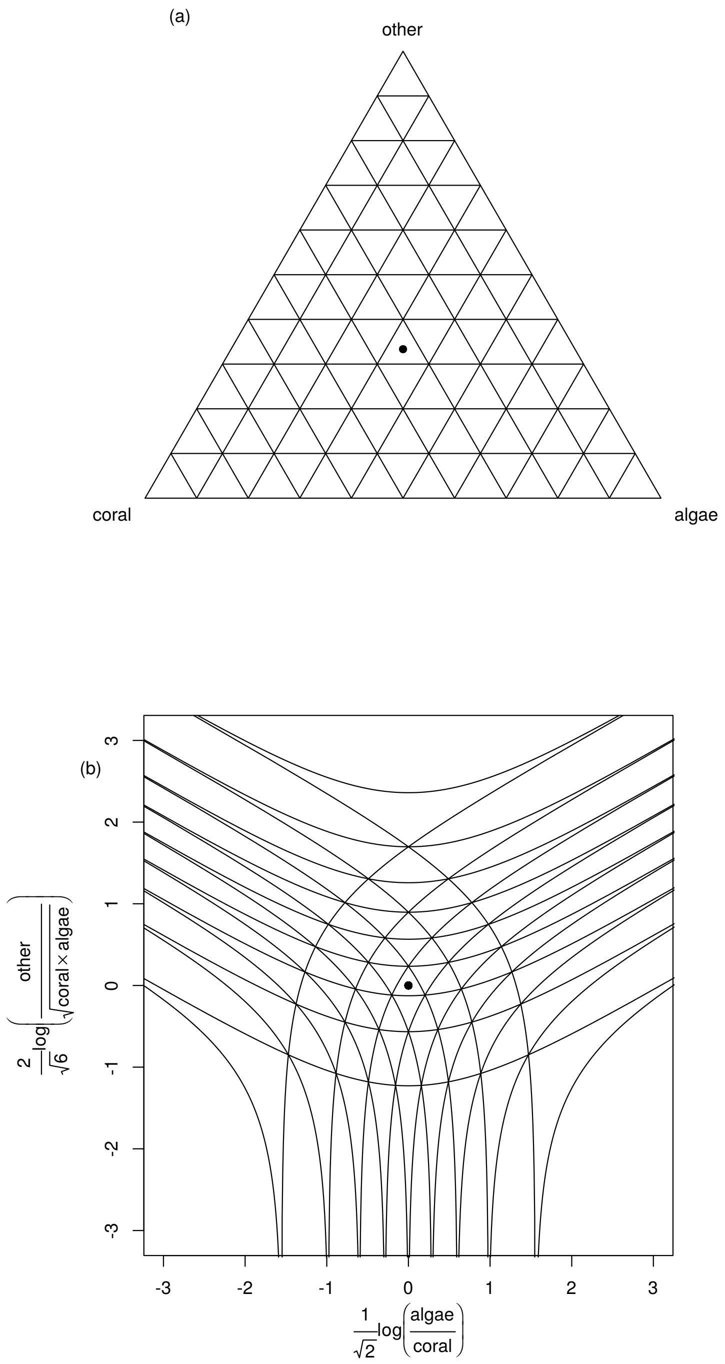

Figure A2: The ilr transformation given by Equation A.1. (a) The open 2-simplex $\mathbb{S}^3$, in which three-part compositions lie. The dot represents the composition with equal relative abundances of coral, algae and other. Lines are contours of constant relative abundance of one part. (b) The ilr-transformed composition in $\mathbb{R}^2$, with dot and contours as in (a).

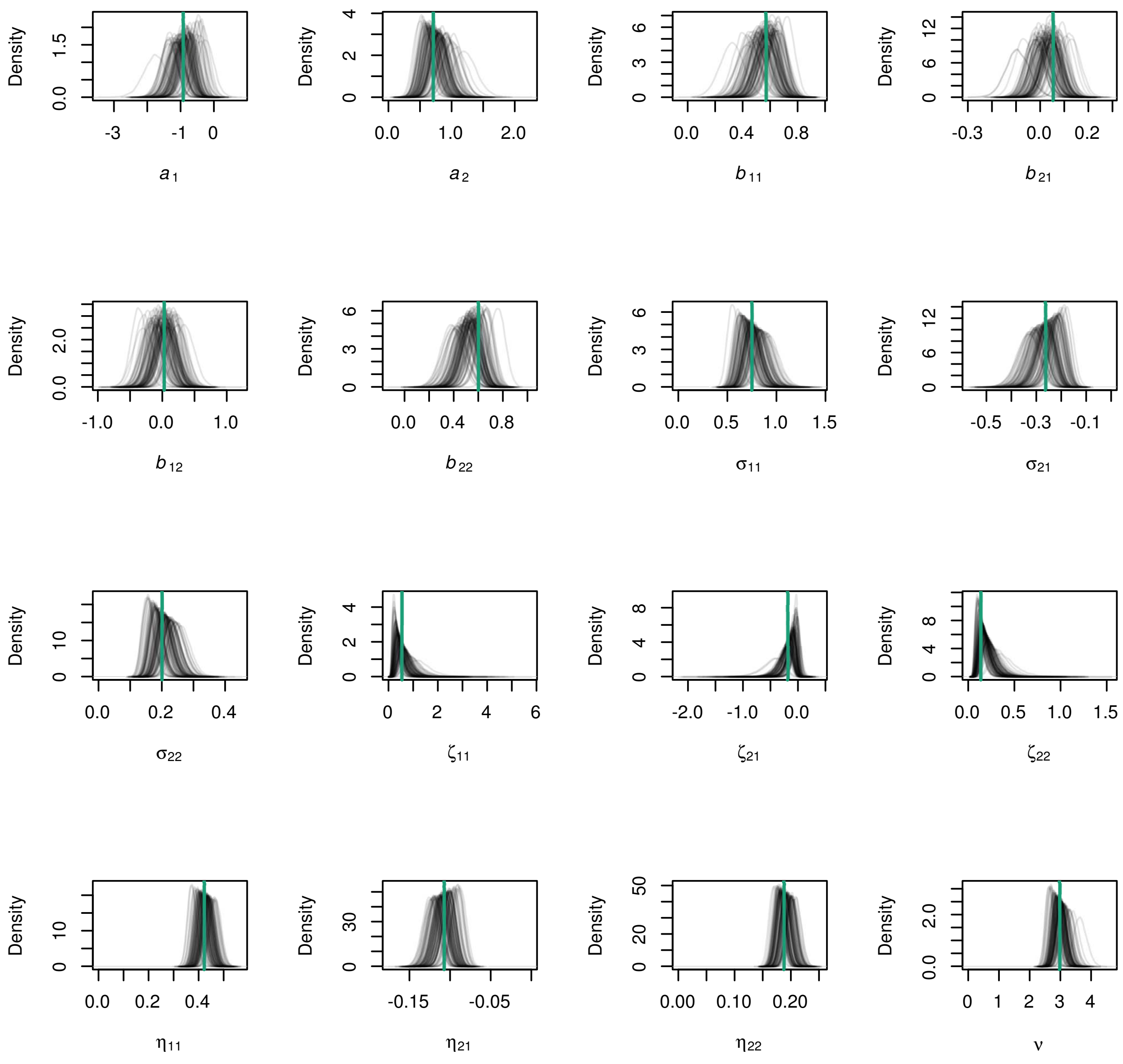

Figure A3: Posterior distributions of parameters estimated from simulated data. Thick green vertical lines: parameter values used to generate simulated data (posterior means from real data). Black lines: kernel density estimates of posterior distributions from 100 simulated data sets, each with the same number of sites, number and spacing of observation times, and numbers of transects at each observation time, as the real data. Number of simulated data sets in which true value was within 95% HPD interval: 89 (a_1), 95 (a_2), 97 (b_{11}), 91 (b_{21}), 95 (b_{12}), 90 (b_{22}), 99 (σ_{11}), 96 (σ_{21}), 93 (σ_{22}), 96 (ζ_{11}), 93 (ζ_{21}), 98 (ζ_{22}), 93 (η_{11}), 93 (η_{21}), 96 (η_{22}), 93 (ν).

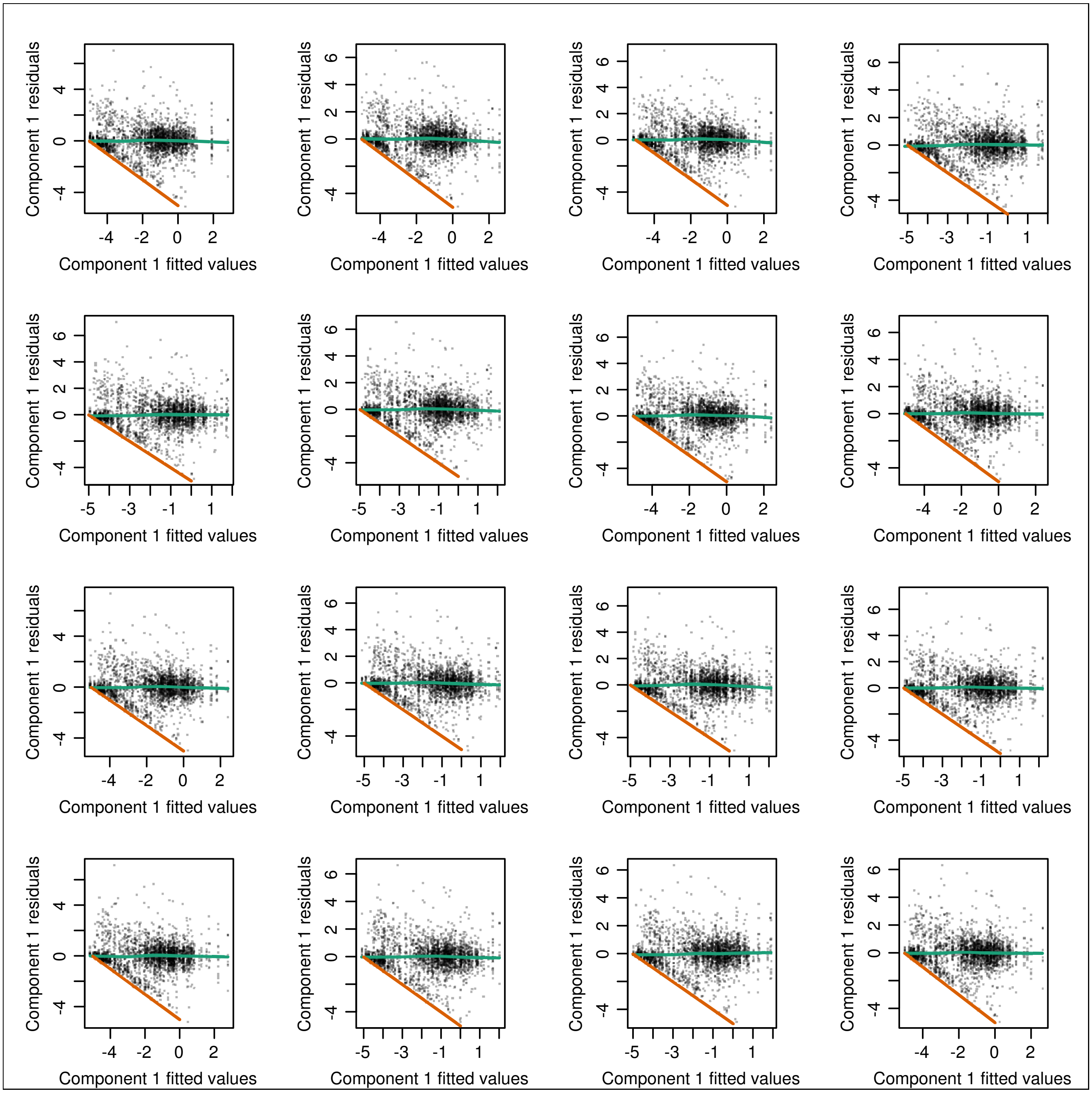

Figure A4: Fitted values against Bayesian residuals for component 1. Each panel is a single randomly-chosen Monte Carlo iteration. Dots represent Bayesian residuals against fitted values for individual transects. The green line is a loess smoother. The orange line is the minimum possible value for component 1 residuals.

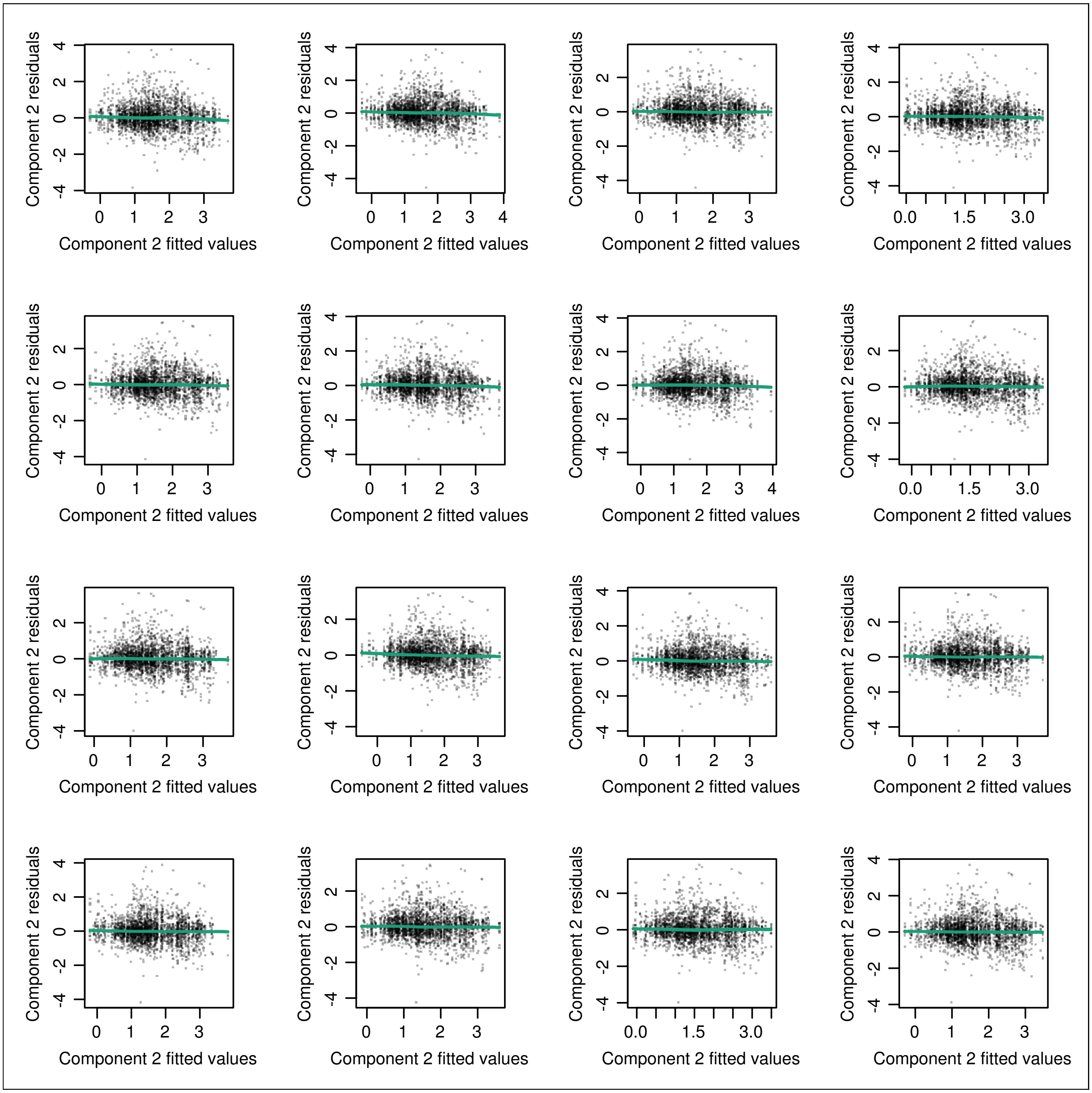

Figure A5: Fitted values against residuals for component 2. Each panel is a single randomly-chosen Monte Carlo iteration. Dots represent Bayesian residuals against fitted values for individual transects. The green line is a loess smoother.

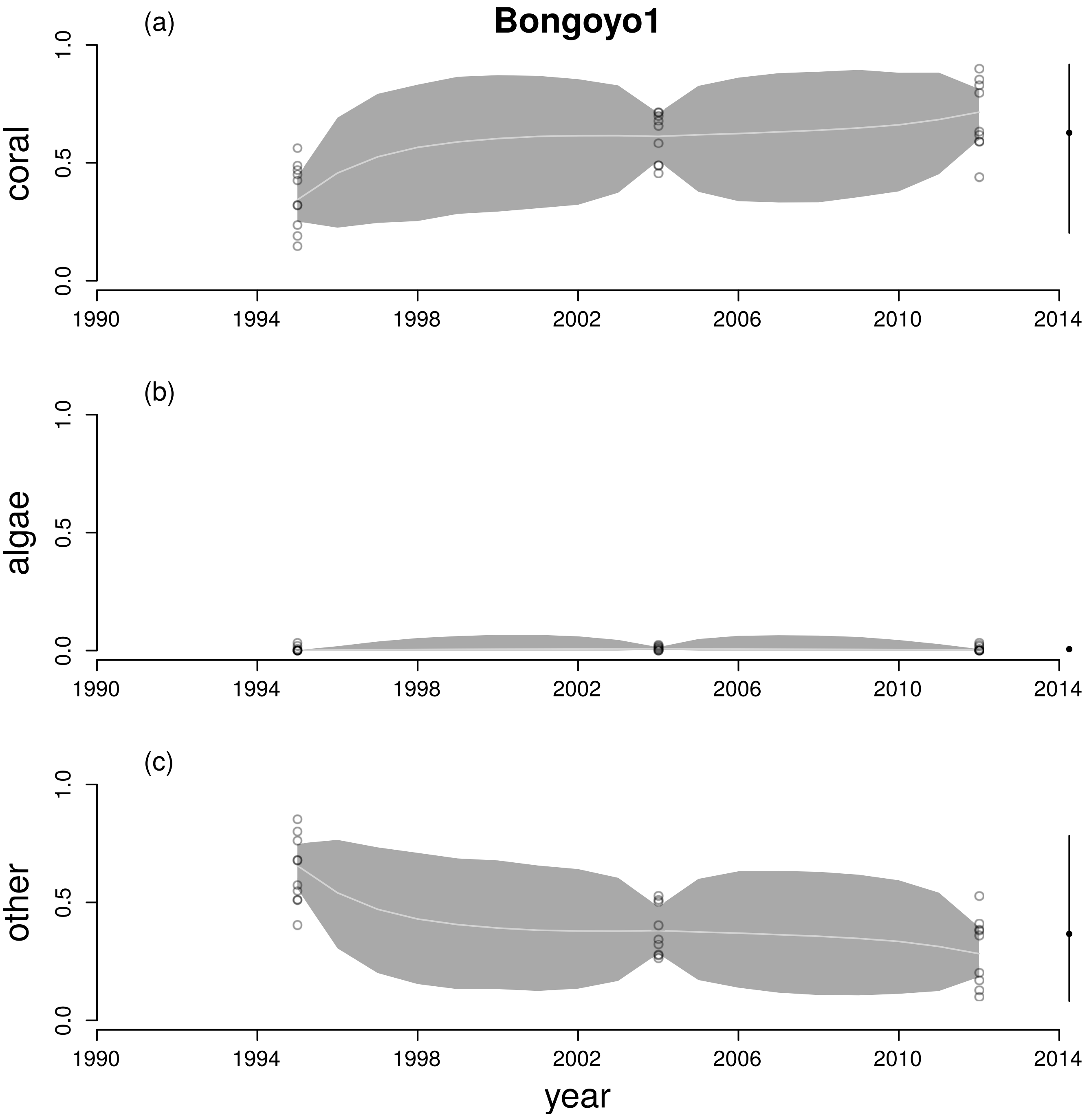

Figure A6: Time series for cover of hard corals (a), macroalgae (b) and other (c) at Bongoyo1. Circles are observations from individual transects. Grey lines join back-transformed posterior mean true states from Equation A.2 and the shaded region is a 95% HPD band. The stationary mean composition for the site is the black dot after the time series and the bar is a 95% HPD interval.

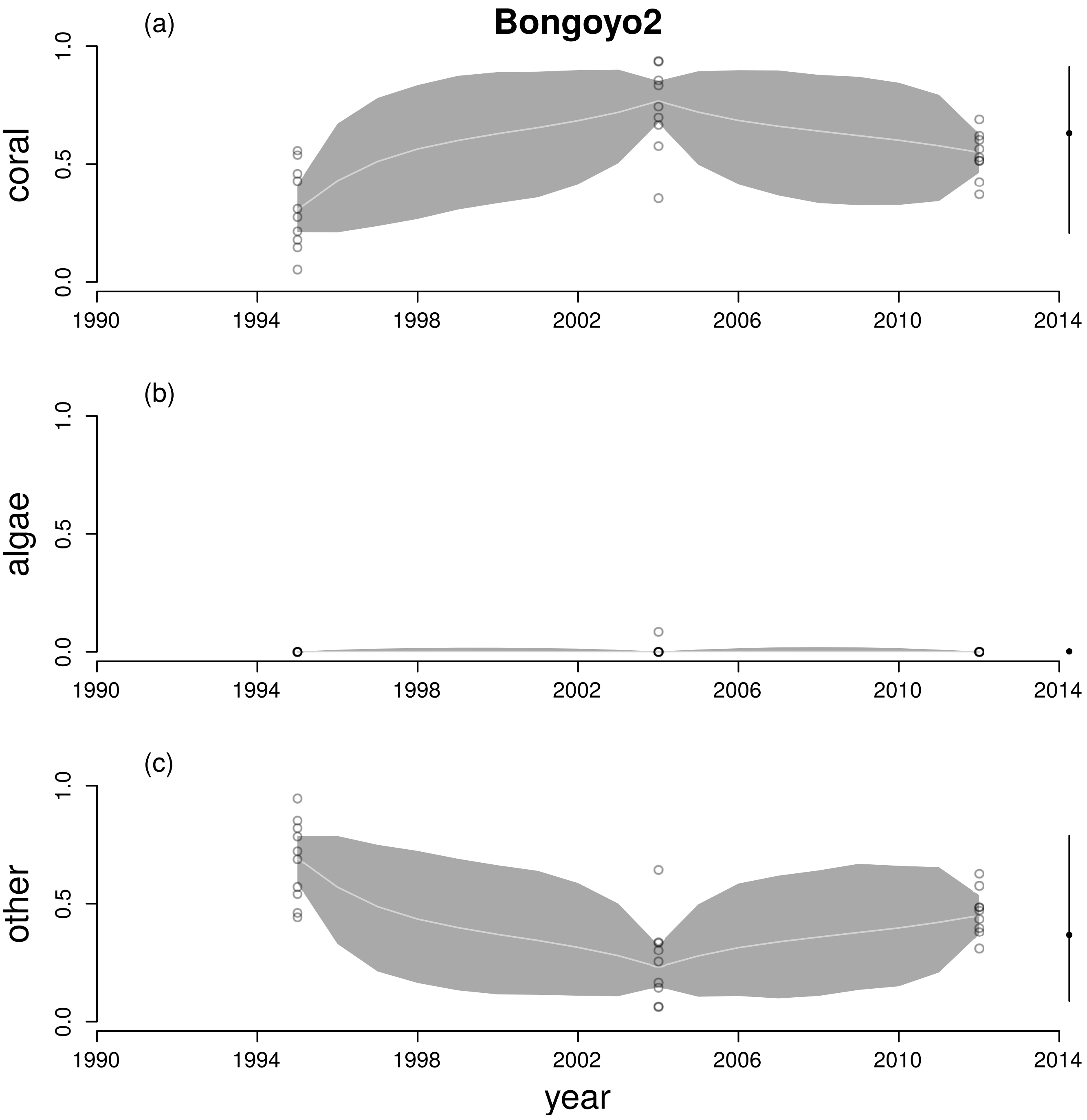

Figure A7: Time series for cover of hard corals (a), macroalgae (b) and other (c) at Bongoyo2. See Figure A6 legend for explanation.

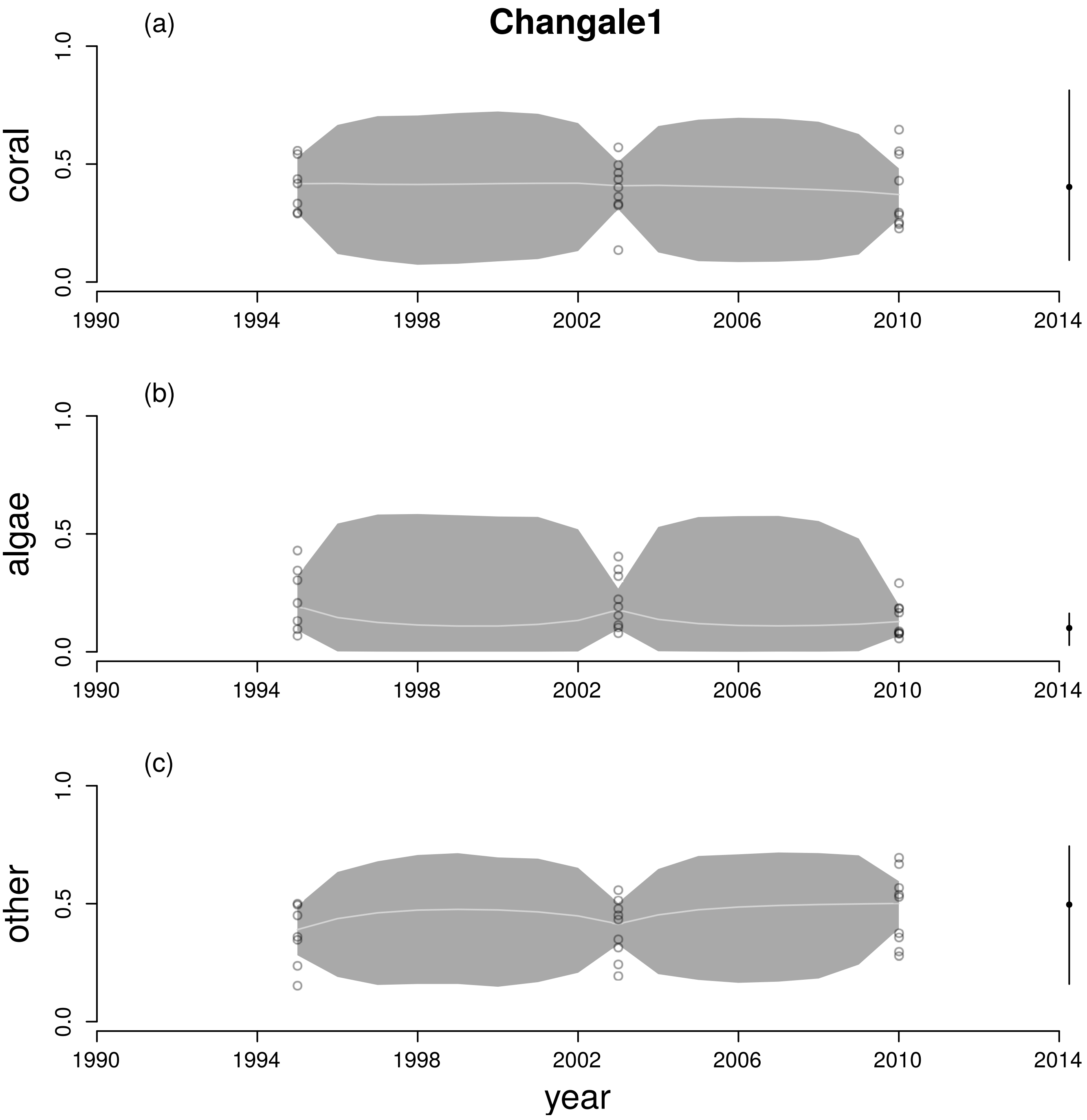

Figure A8: Time series for cover of hard corals (a), macroalgae (b) and other (c) at Changale1. See Figure A6 legend for explanation.

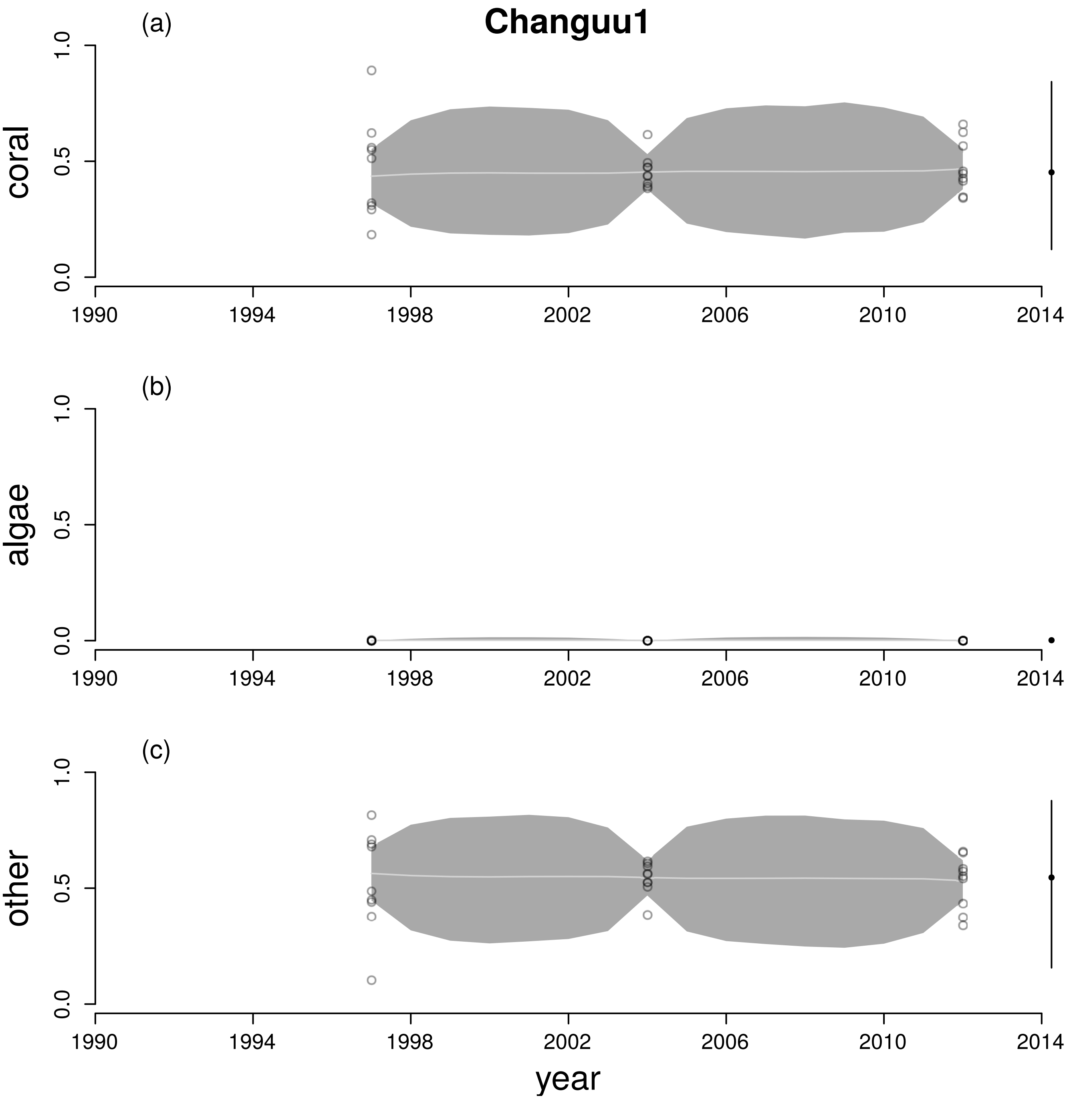

Figure A9: Time series for cover of hard corals (a), macroalgae (b) and other (c) at Changuu1. See Figure A6 legend for explanation.

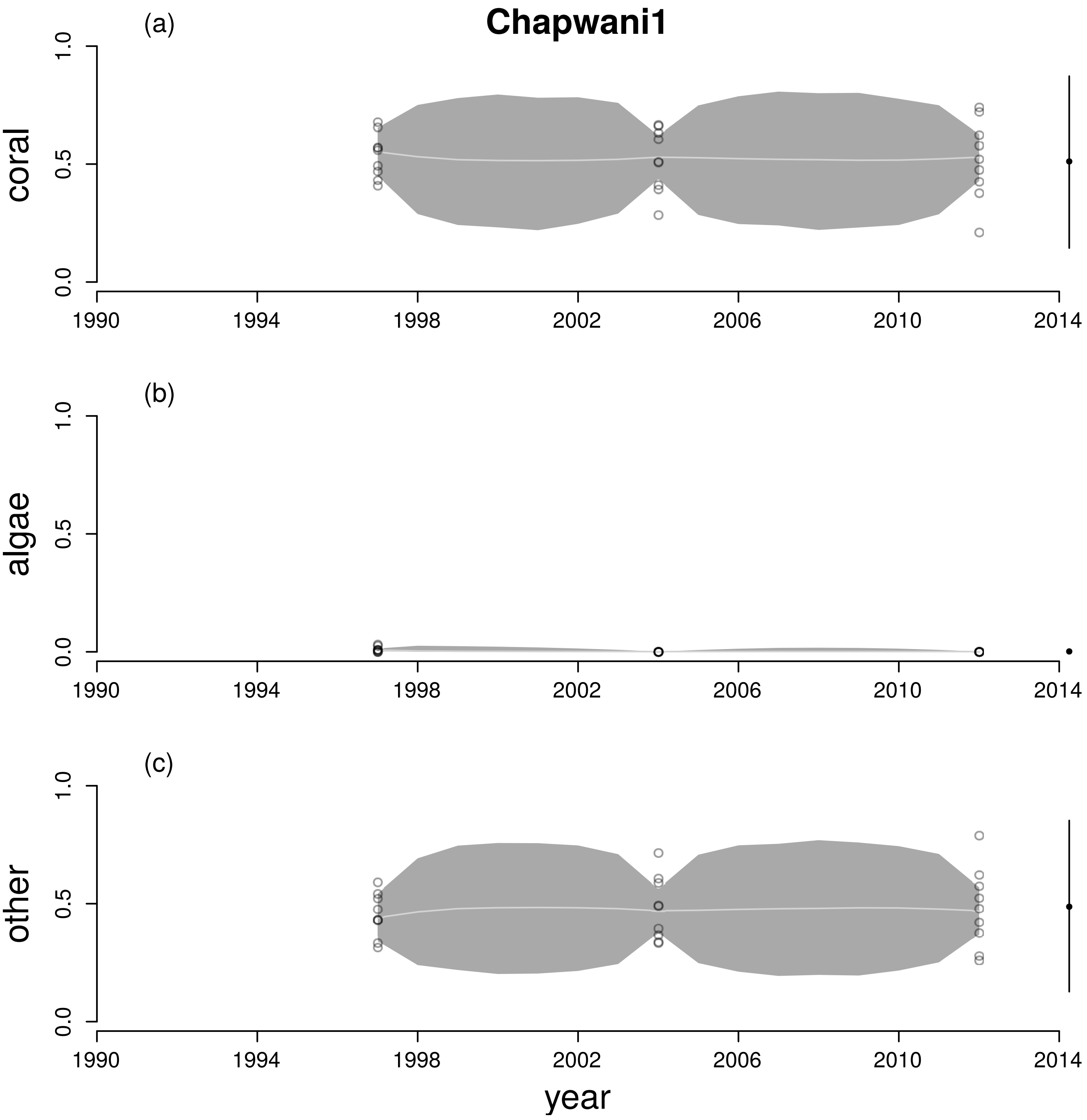

Figure A10: Time series for cover of hard corals (a), macroalgae (b) and other (c) at Chapwani1. See Figure A6 legend for explanation.

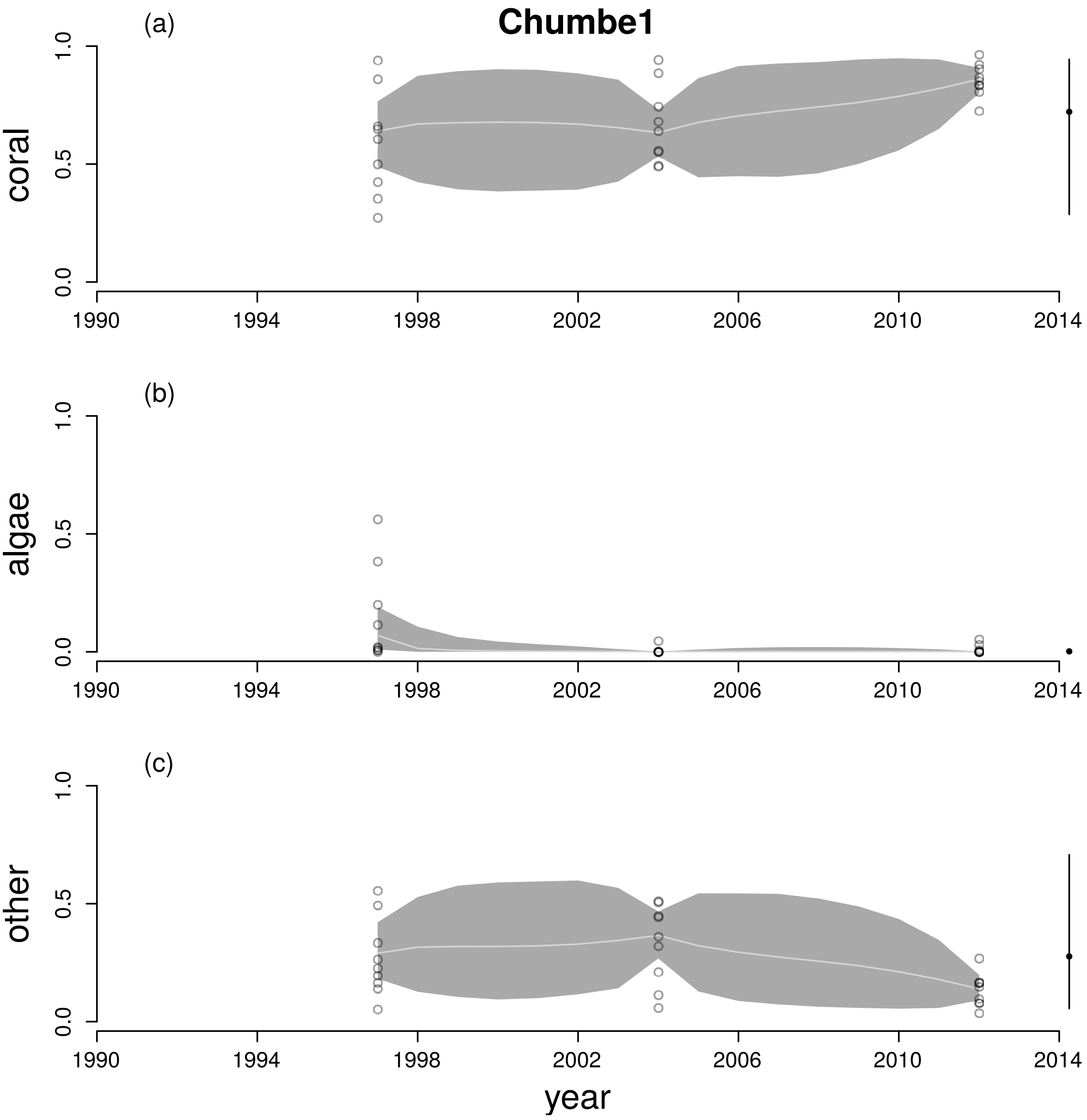

Figure A11: Time series for cover of hard corals (a), macroalgae (b) and other (c) at Chumbe1. See Figure A6 legend for explanation.

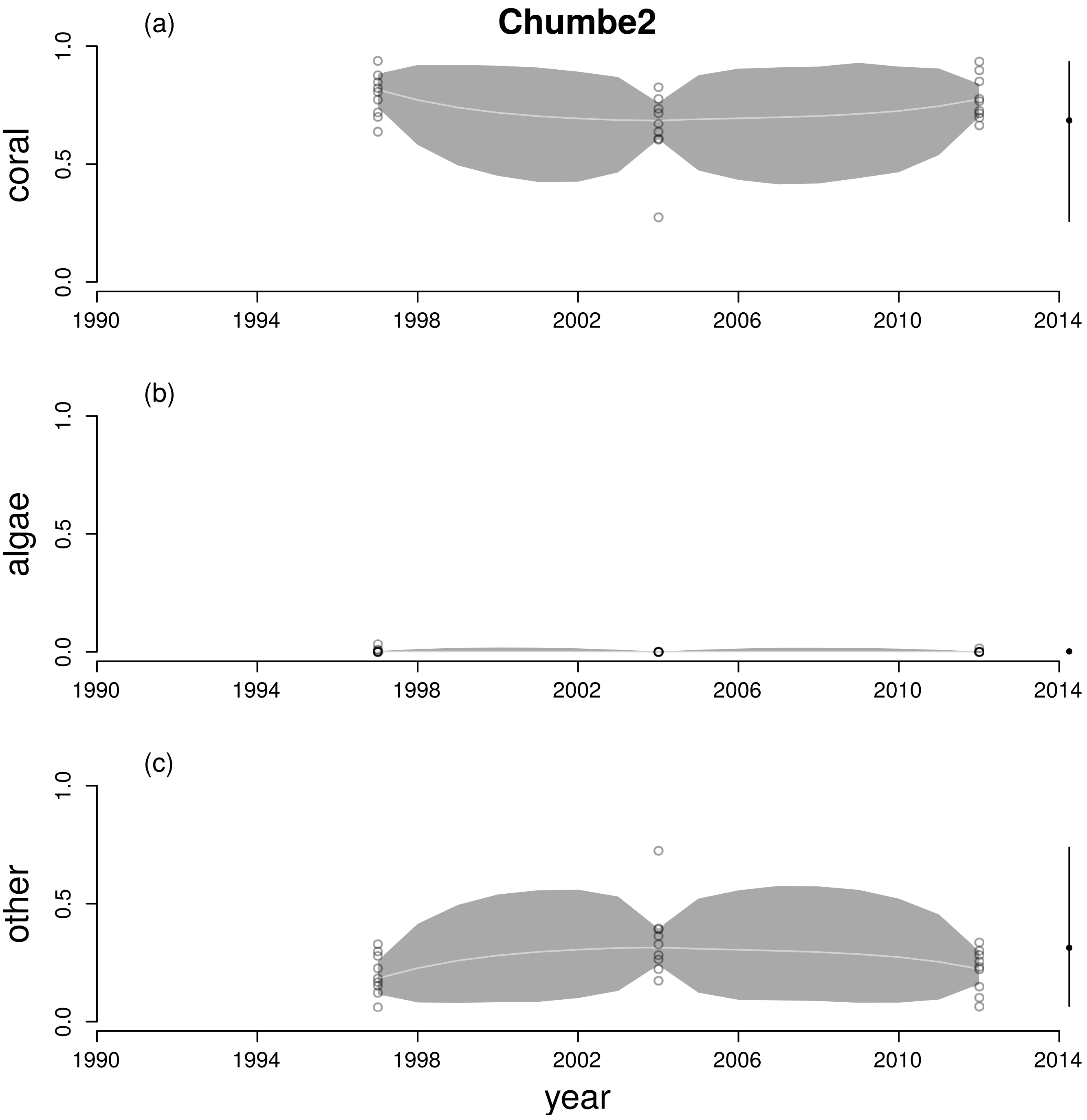

Figure A12: Time series for cover of hard corals (a), macroalgae (b) and other (c) at Chumbe2. See Figure A6 legend for explanation.

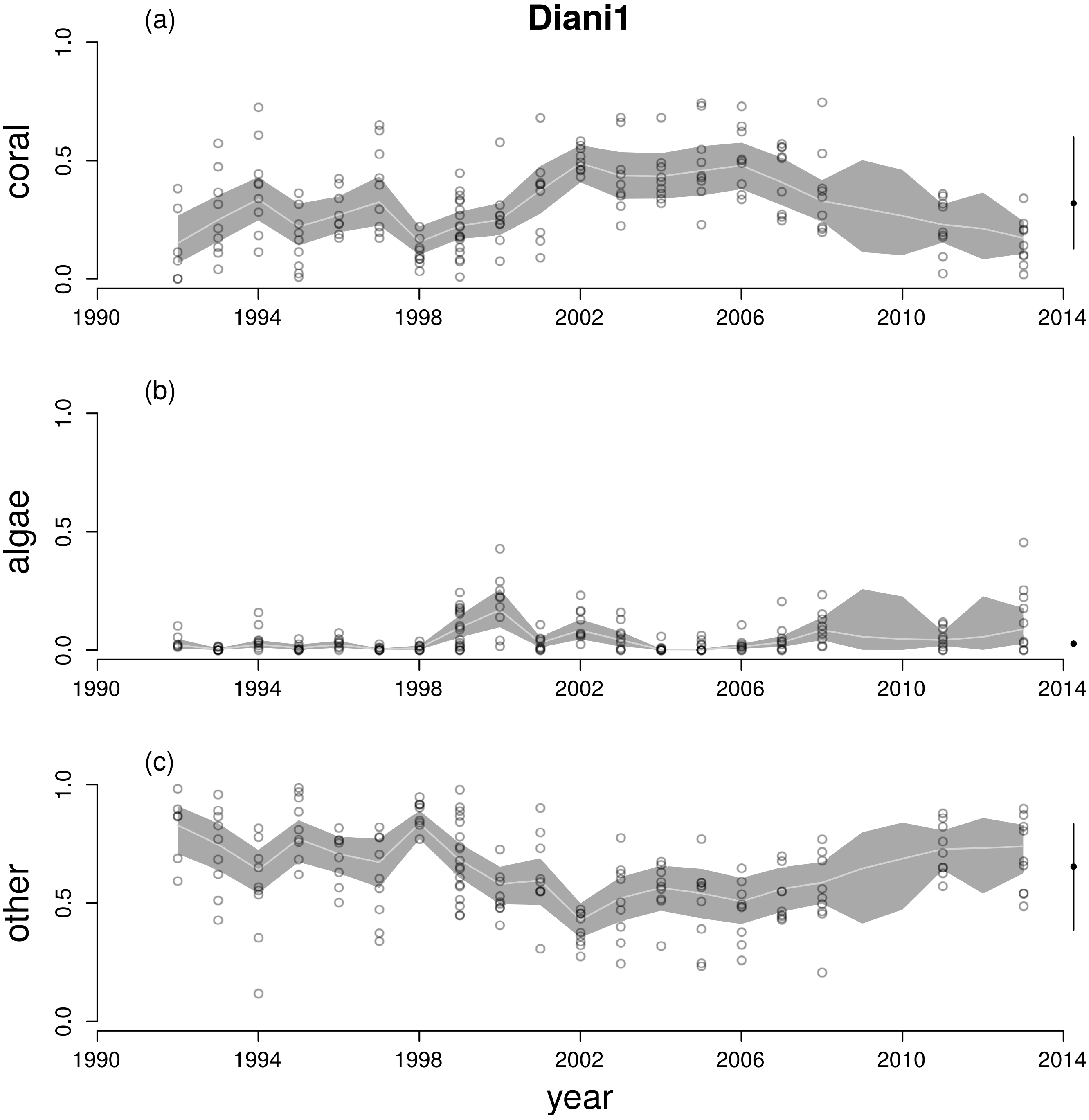

Figure A13: Time series for cover of hard corals (a), macroalgae (b) and other (c) at Diani1. See Figure A6 legend for explanation.

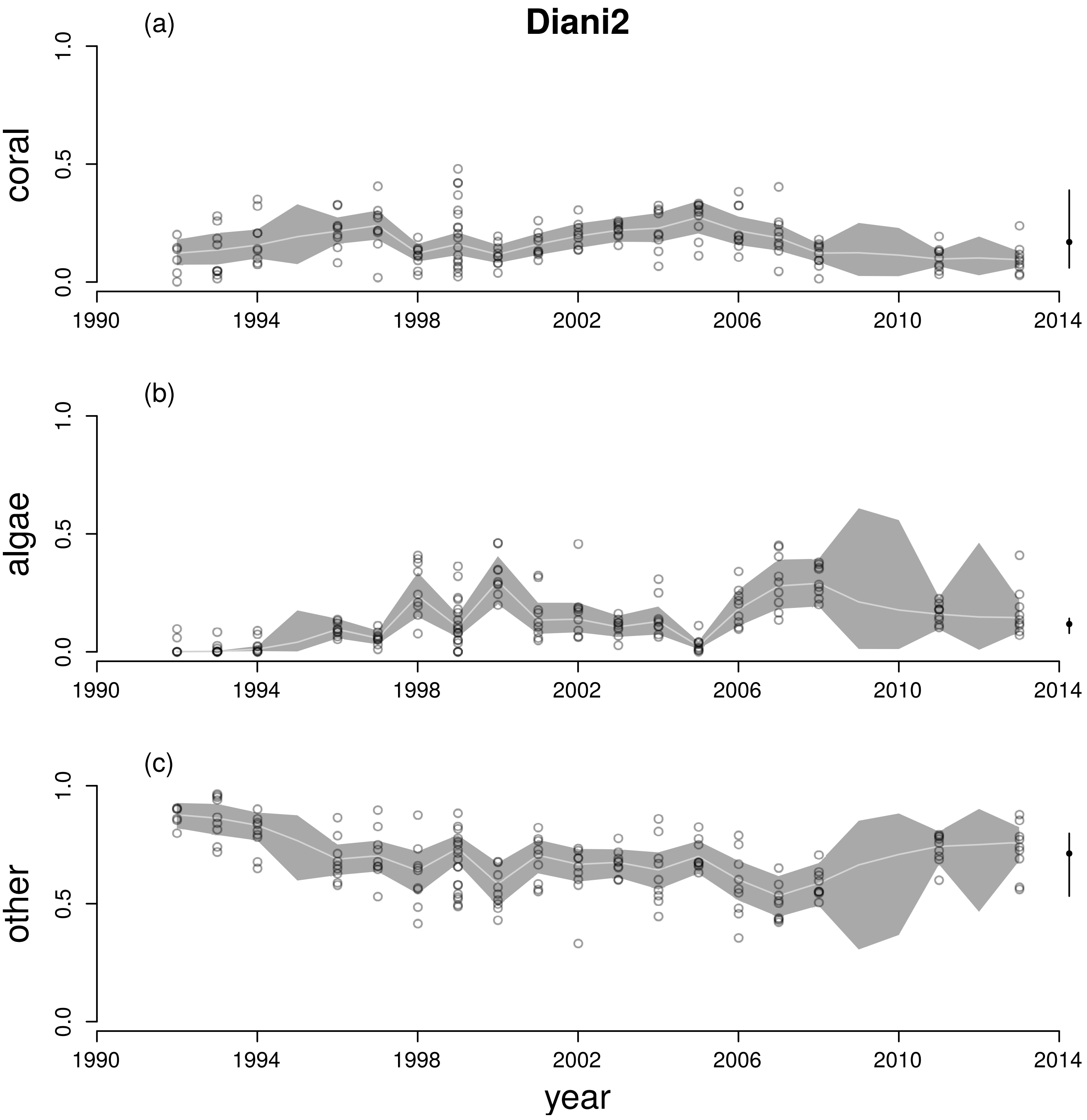

Figure A14: Time series for cover of hard corals (a), macroalgae (b) and other (c) at Diani2. See Figure A6 legend for explanation.

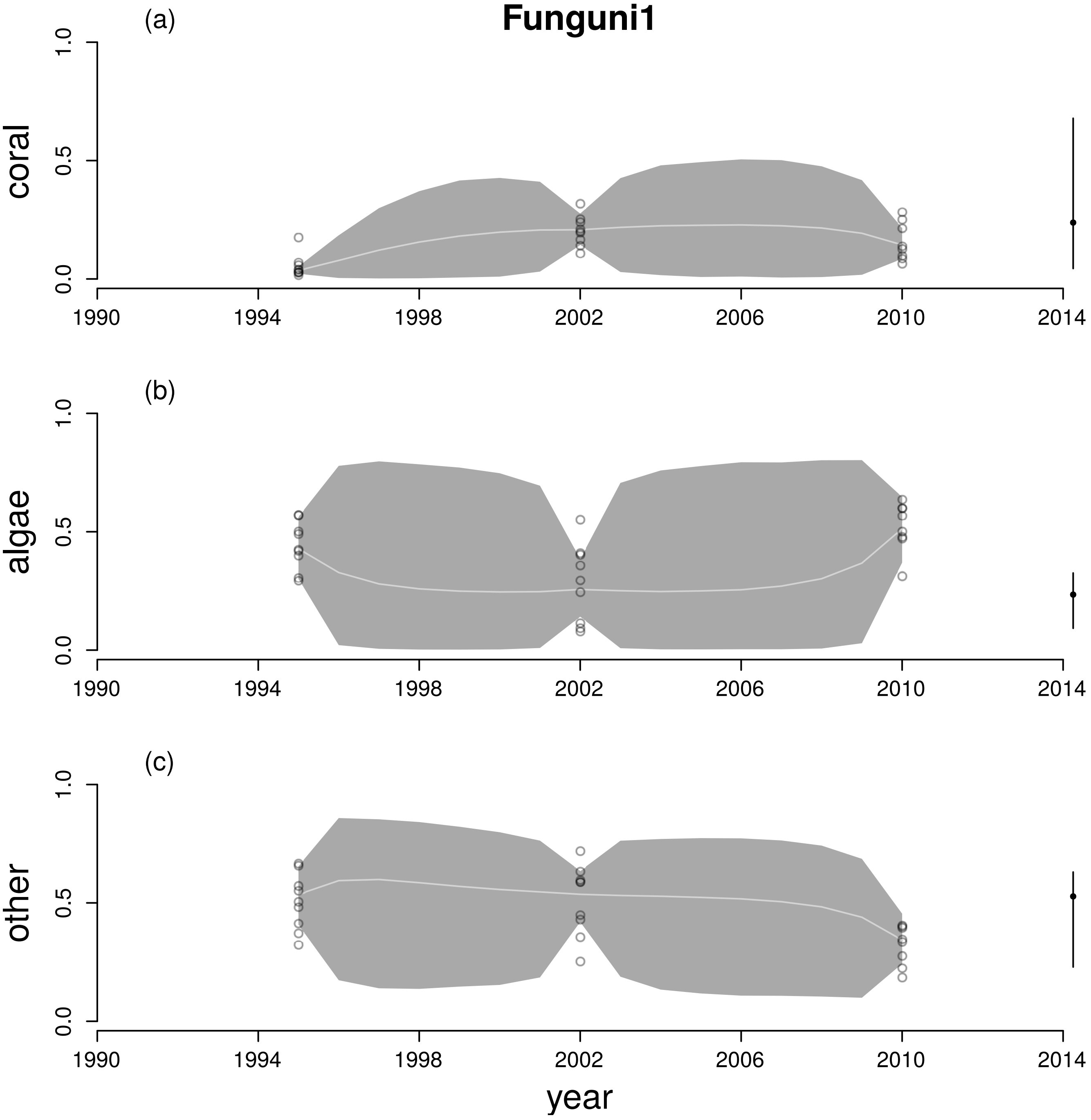

Figure A15: Time series for cover of hard corals (a), macroalgae (b) and other (c) at Funguni1. See Figure A6 legend for explanation.

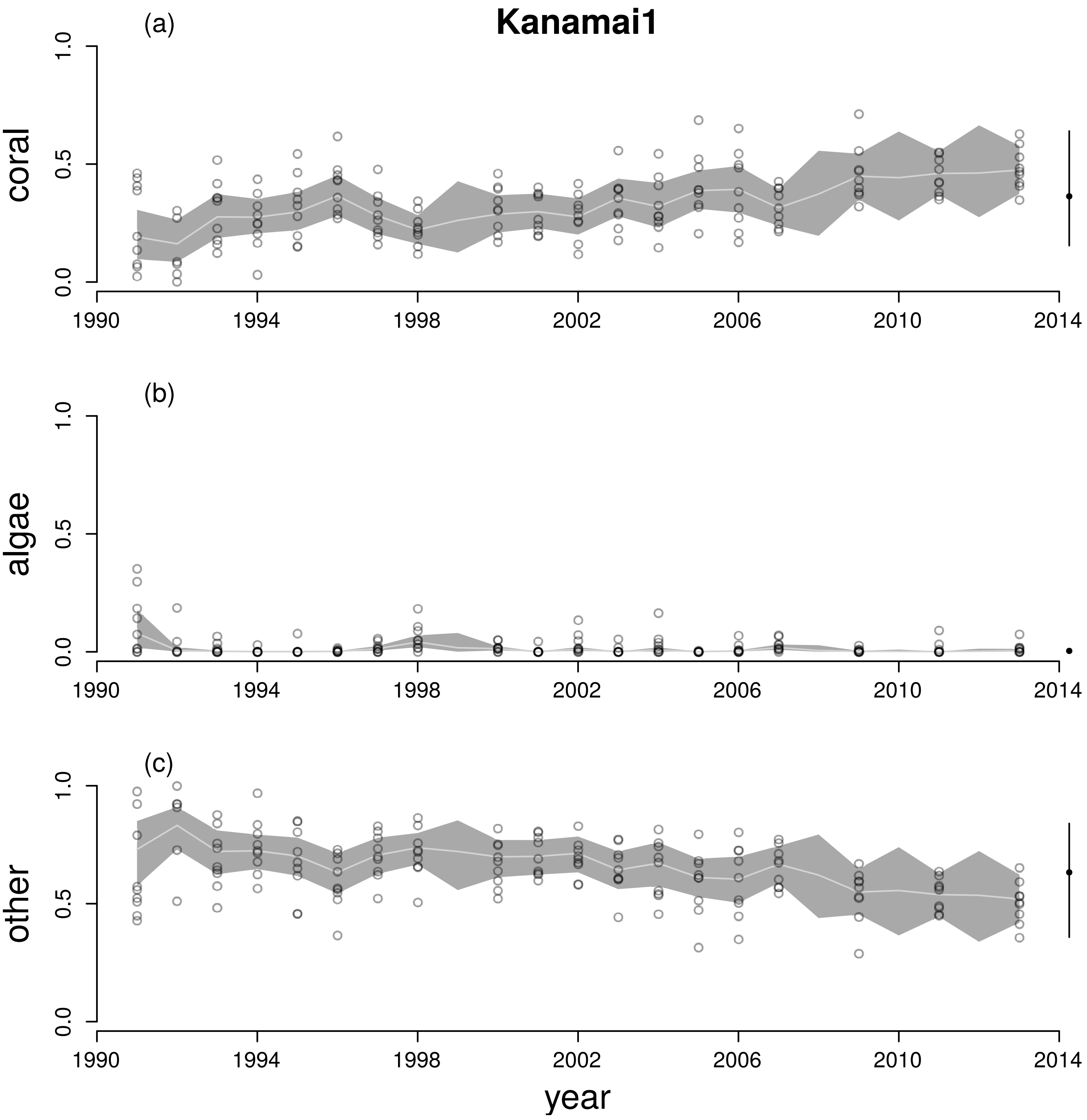

Figure A16: Time series for cover of hard corals (a), macroalgae (b) and other (c) at Kanamai1. See Figure A6 legend for explanation.

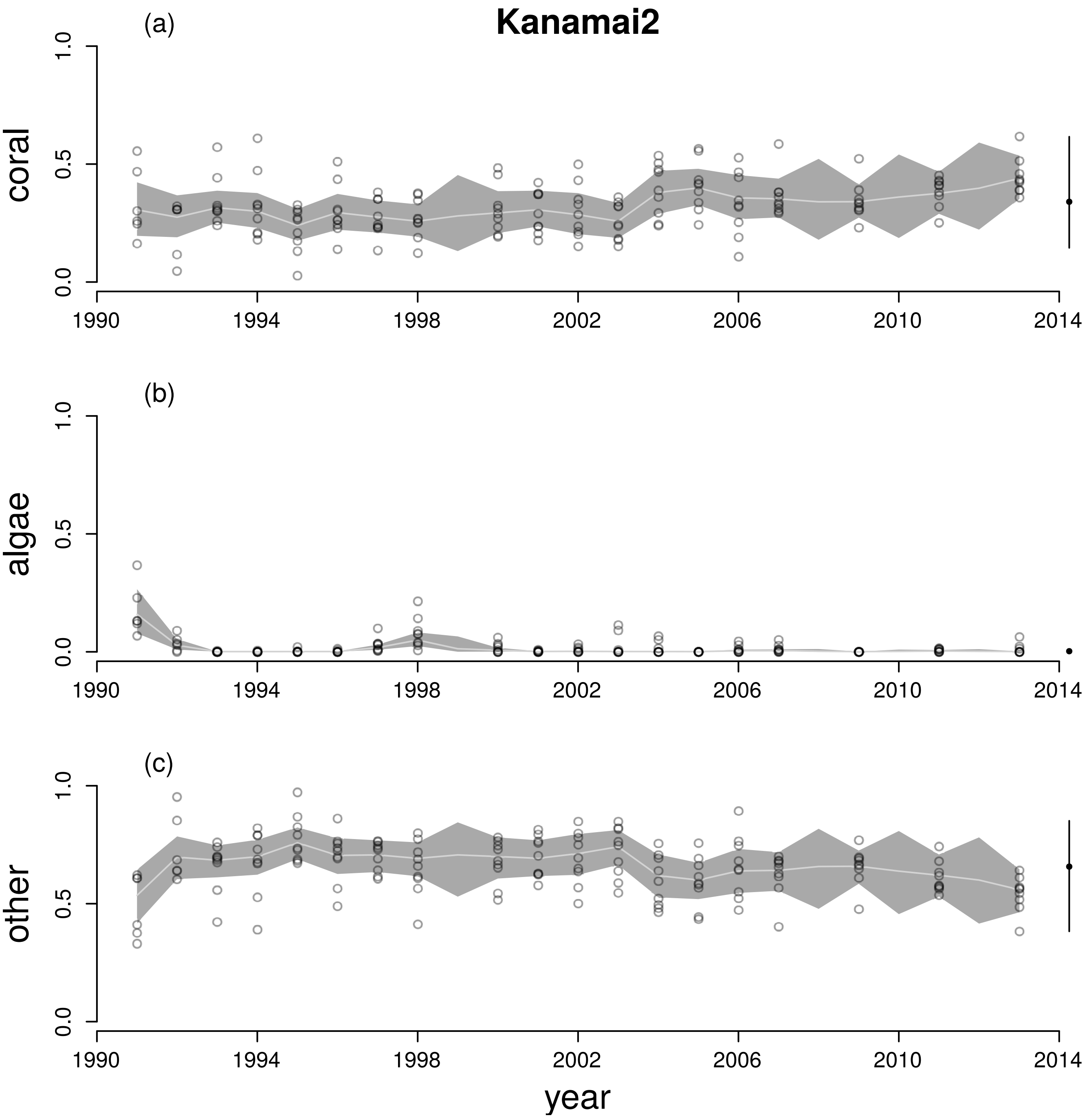

Figure A17: Time series for cover of hard corals (a), macroalgae (b) and other (c) at Kanamai2. See Figure A6 legend for explanation.

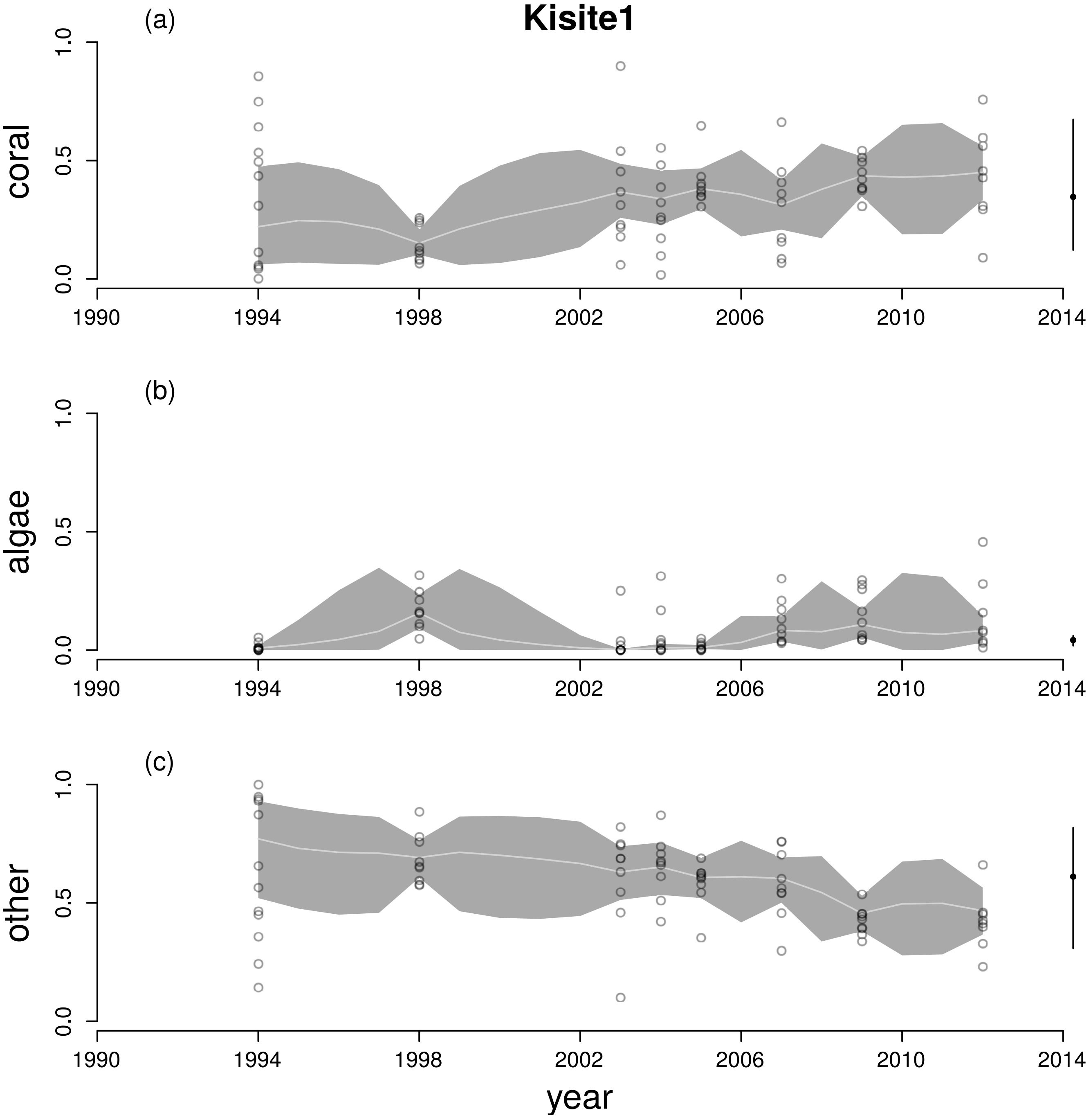

Figure A18: Time series for cover of hard corals (a), macroalgae (b) and other (c) at Kisite1. See Figure A6 legend for explanation.

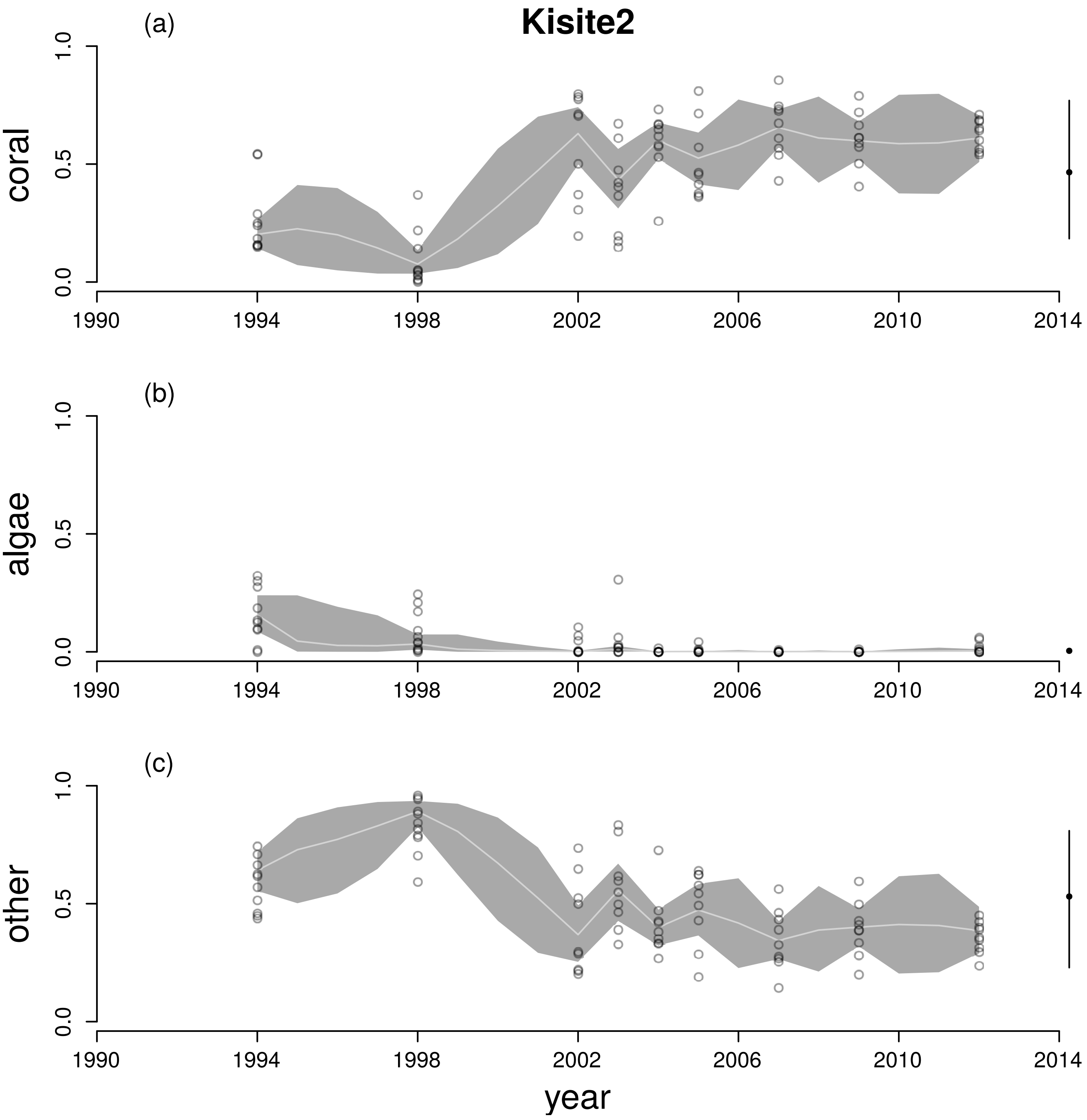

Figure A19: Time series for cover of hard corals (a), macroalgae (b) and other (c) at Kisite2. See Figure A6 legend for explanation.

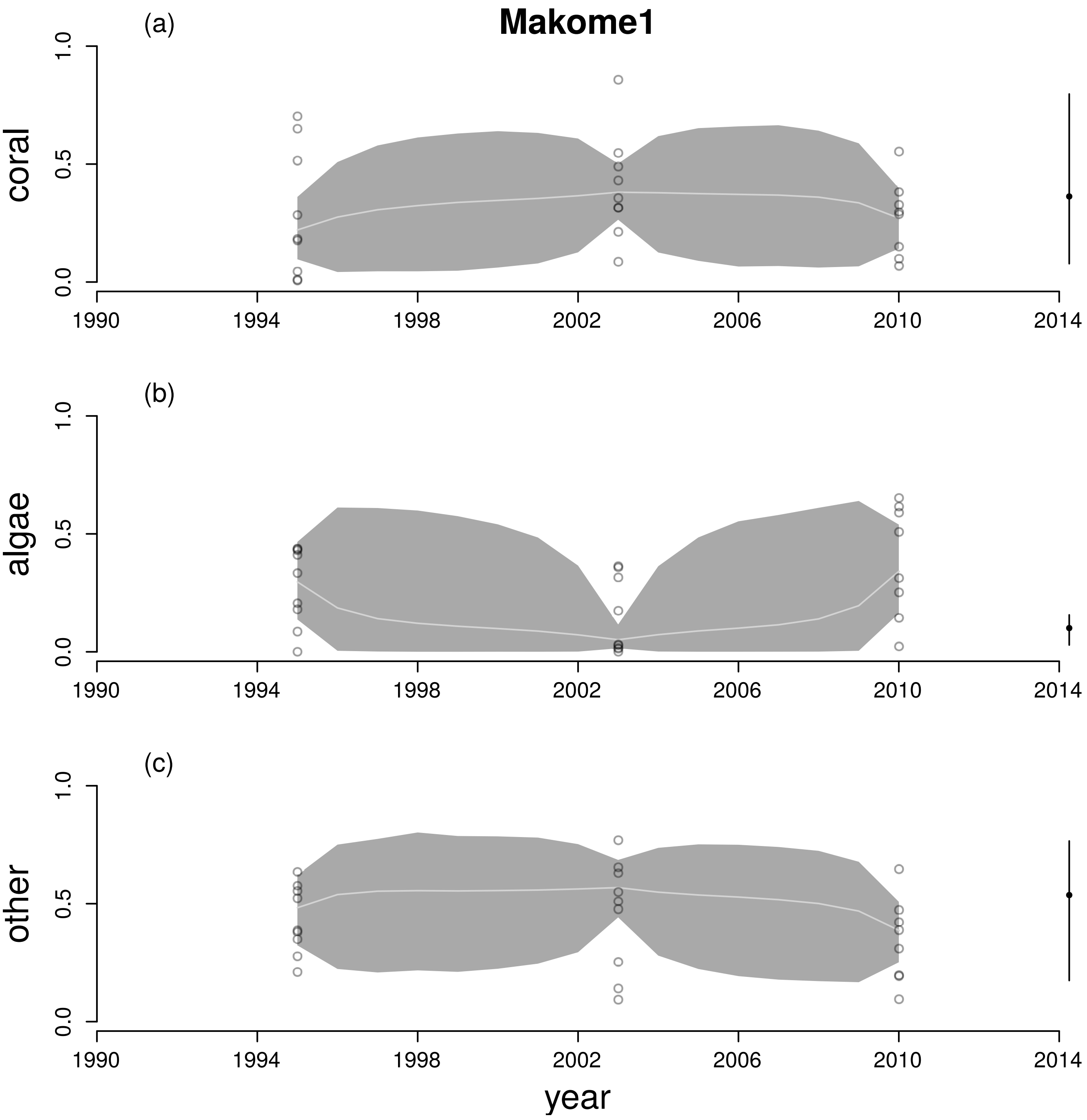

Figure A20: Time series for cover of hard corals (a), macroalgae (b) and other (c) at Makome1. See Figure A6 legend for explanation.

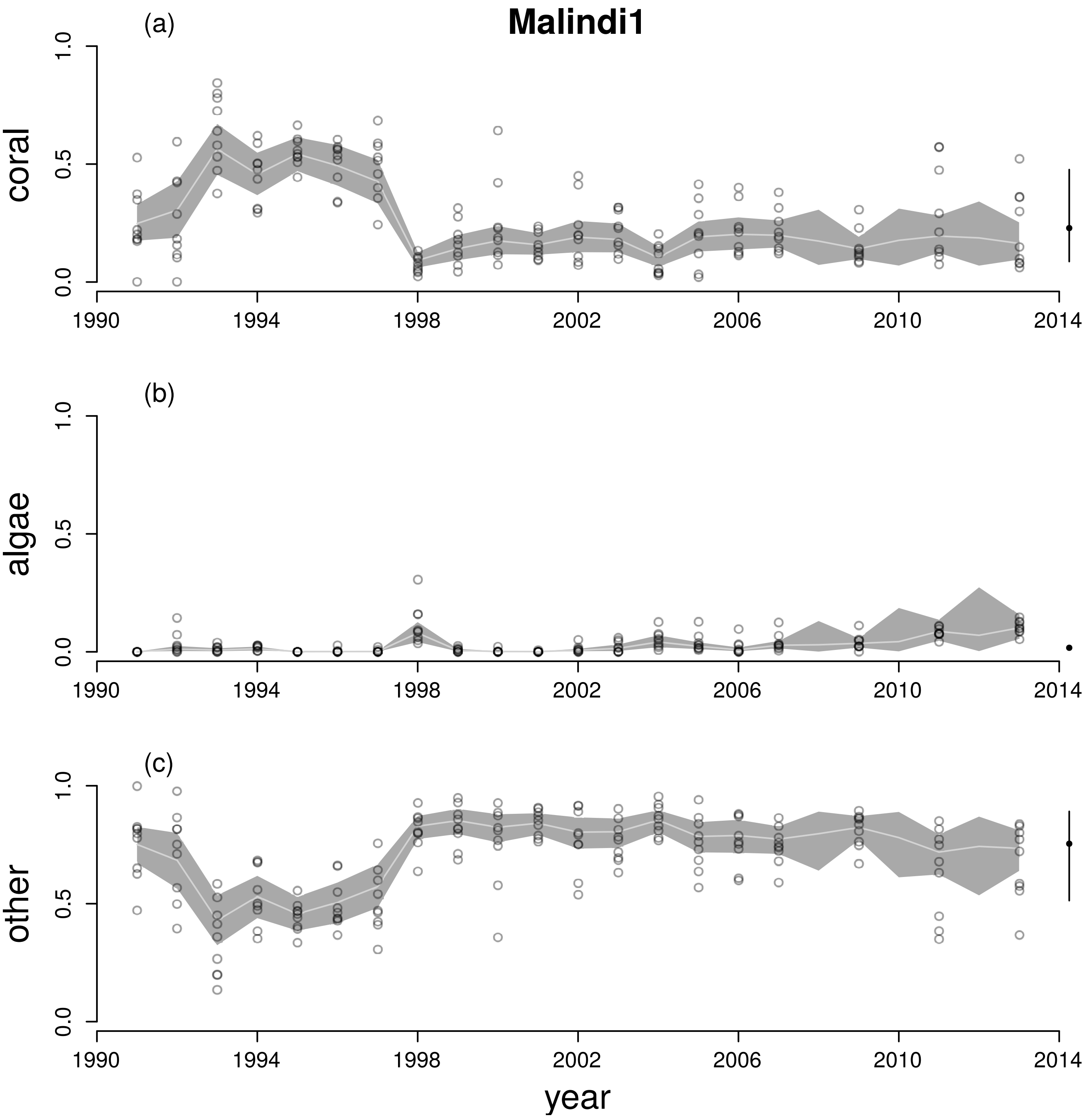

Figure A21: Time series for cover of hard corals (a), macroalgae (b) and other (c) at Malindi1. See Figure A6 legend for explanation.

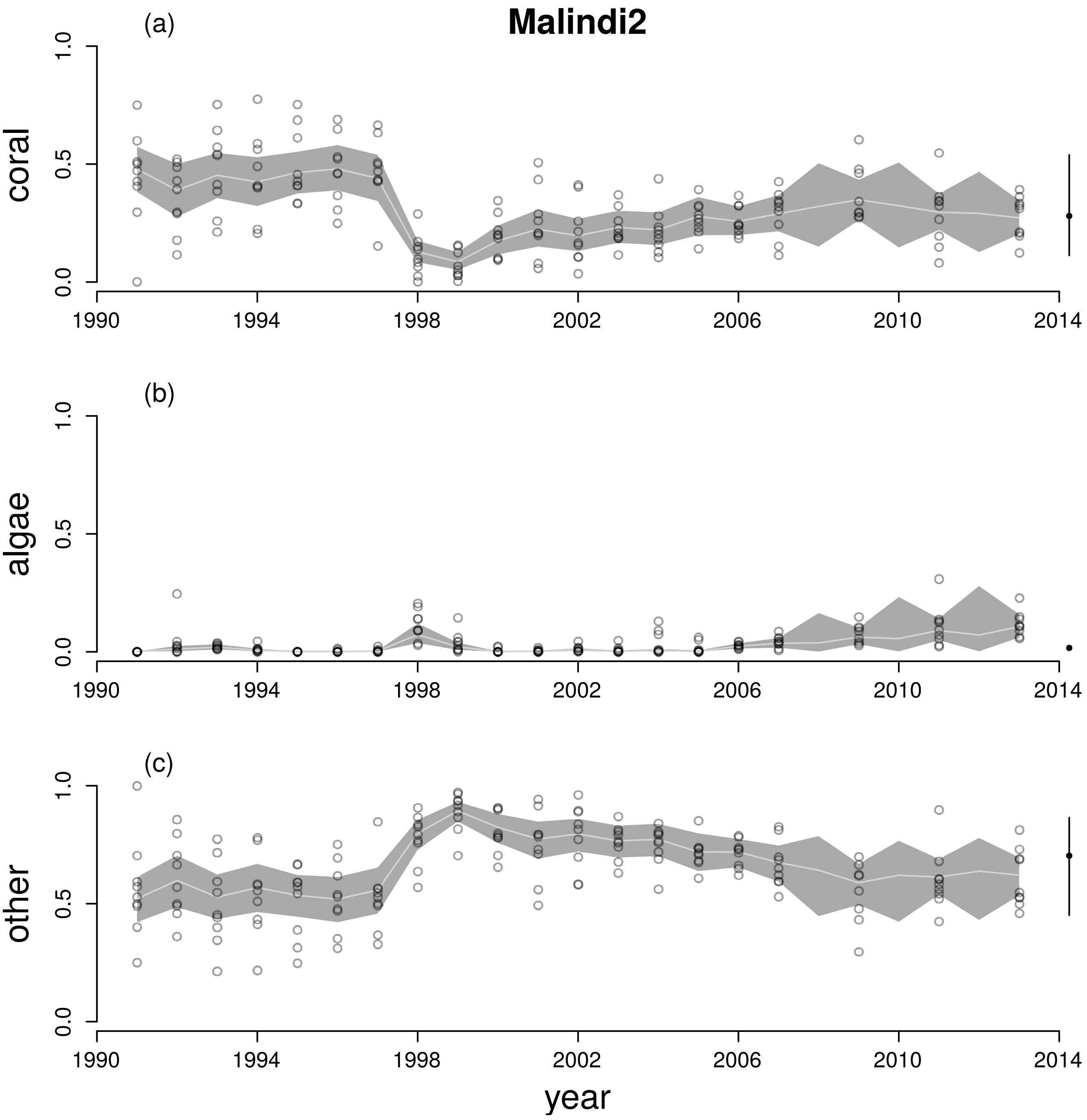

Figure A22: Time series for cover of hard corals (a), macroalgae (b) and other (c) at Malindi2. See Figure A6 legend for explanation.

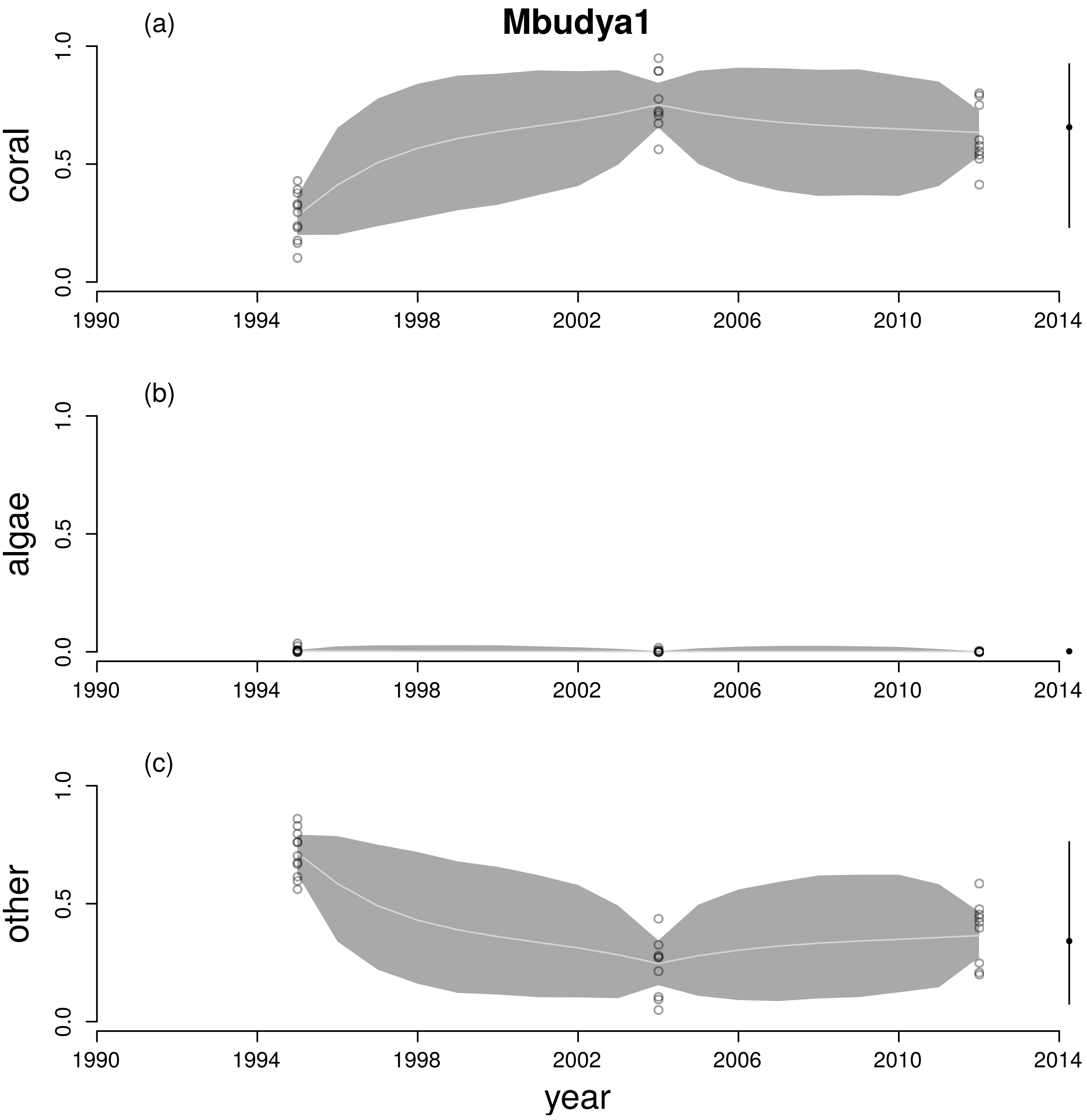

Figure A23: Time series for cover of hard corals (a), macroalgae (b) and other (c) at Mbudya1. See Figure A6 legend for explanation.

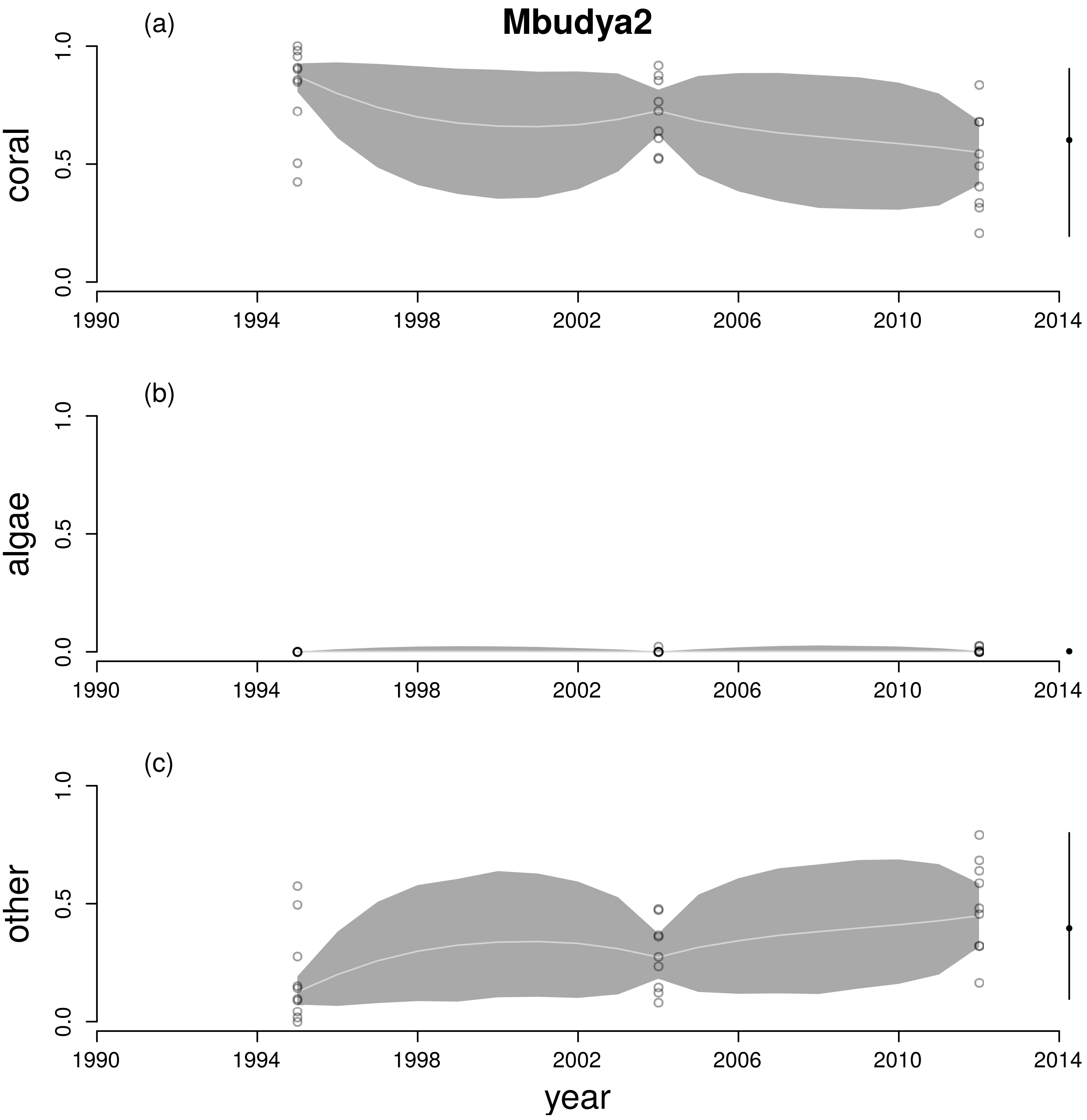

Figure A24: Time series for cover of hard corals (a), macroalgae (b) and other (c) at Mbudya2. See Figure A6 legend for explanation.

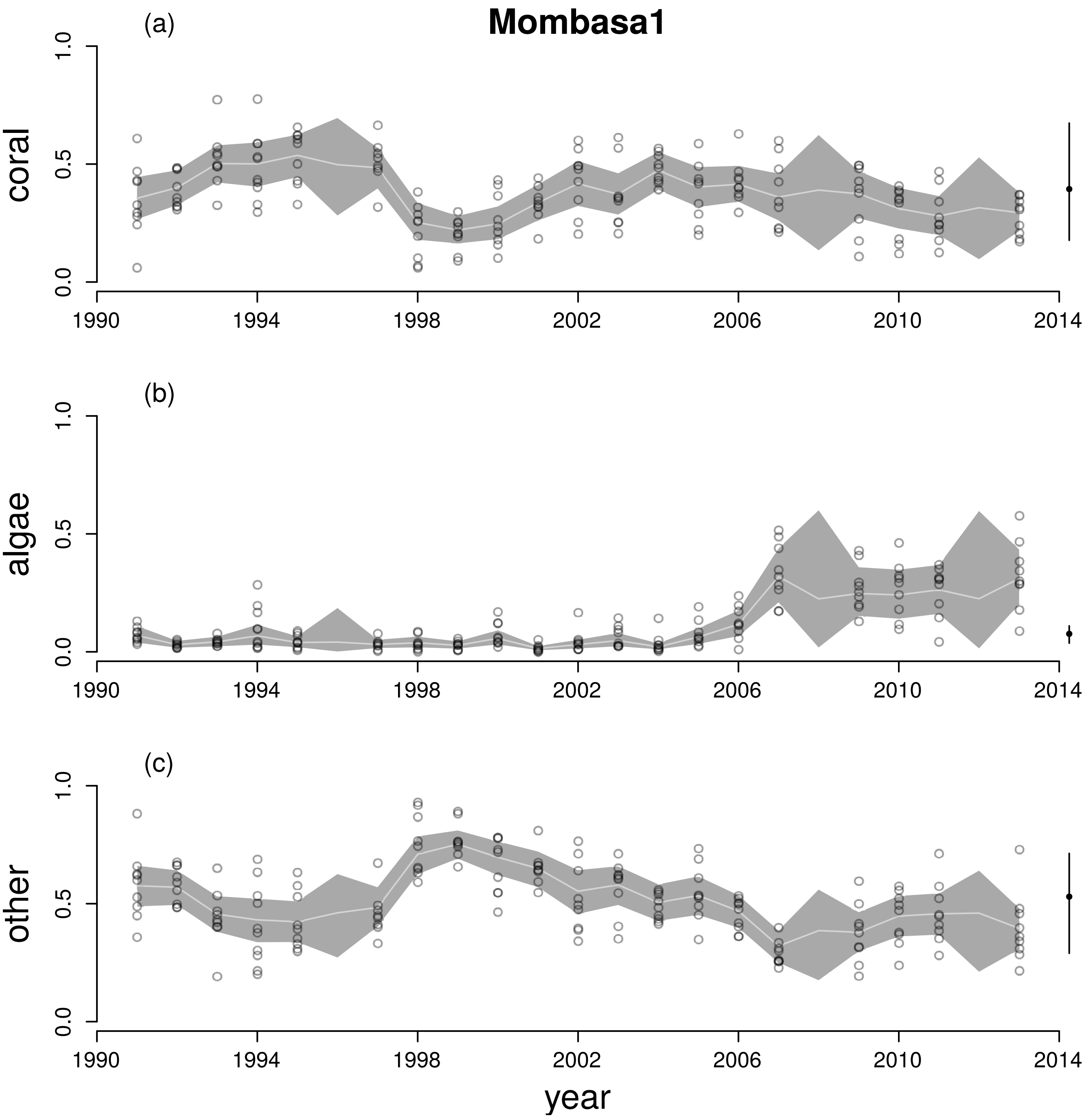

Figure A25: Time series for cover of hard corals (a), macroalgae (b) and other (c) at Mombasa1. See Figure A6 legend for explanation.

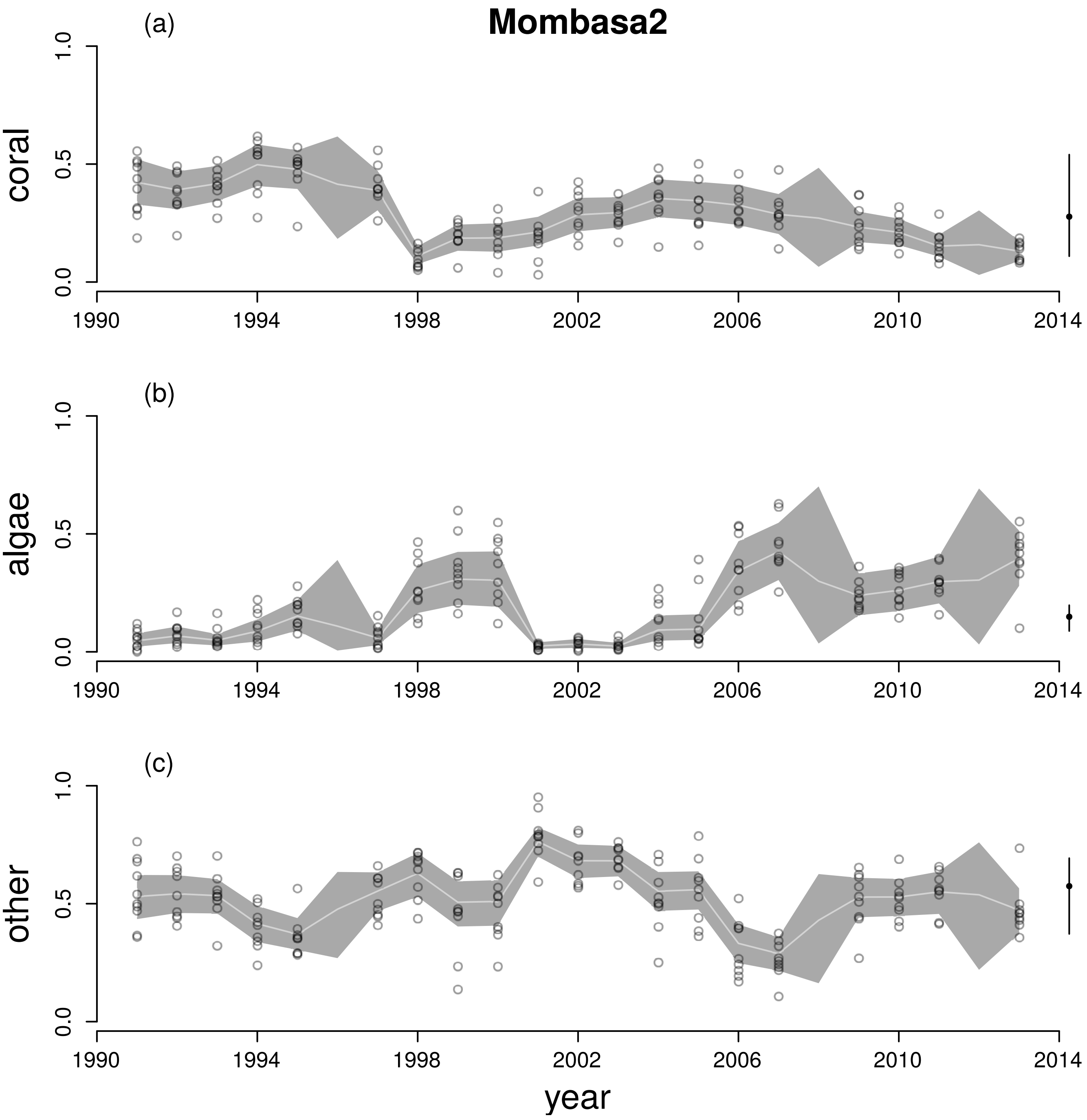

Figure A26: Time series for cover of hard corals (a), macroalgae (b) and other (c) at Mombasa2. See Figure A6 legend for explanation.

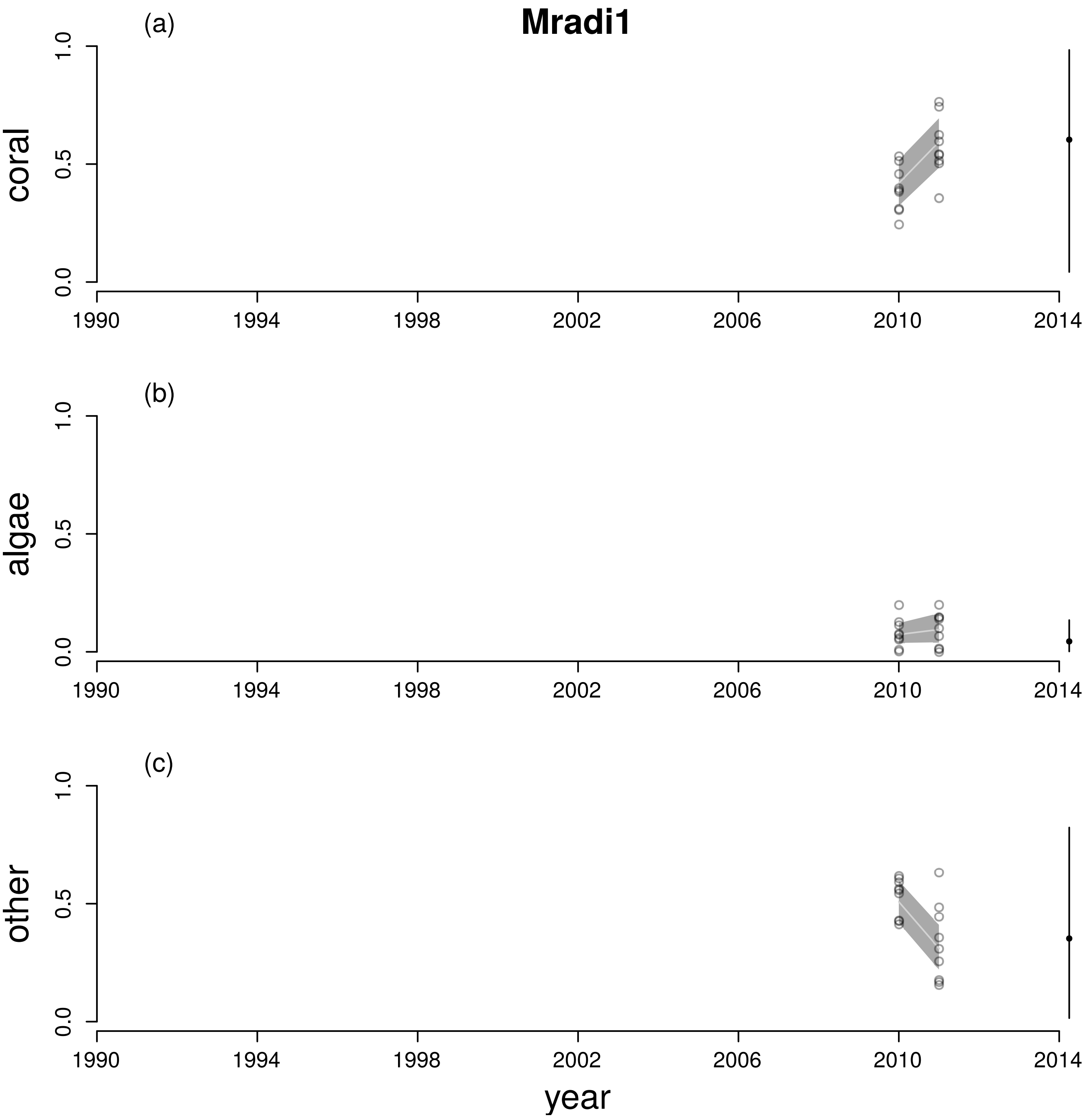

Figure A27: Time series for cover of hard corals (a), macroalgae (b) and other (c) at Mradi1. See Figure A6 legend for explanation.

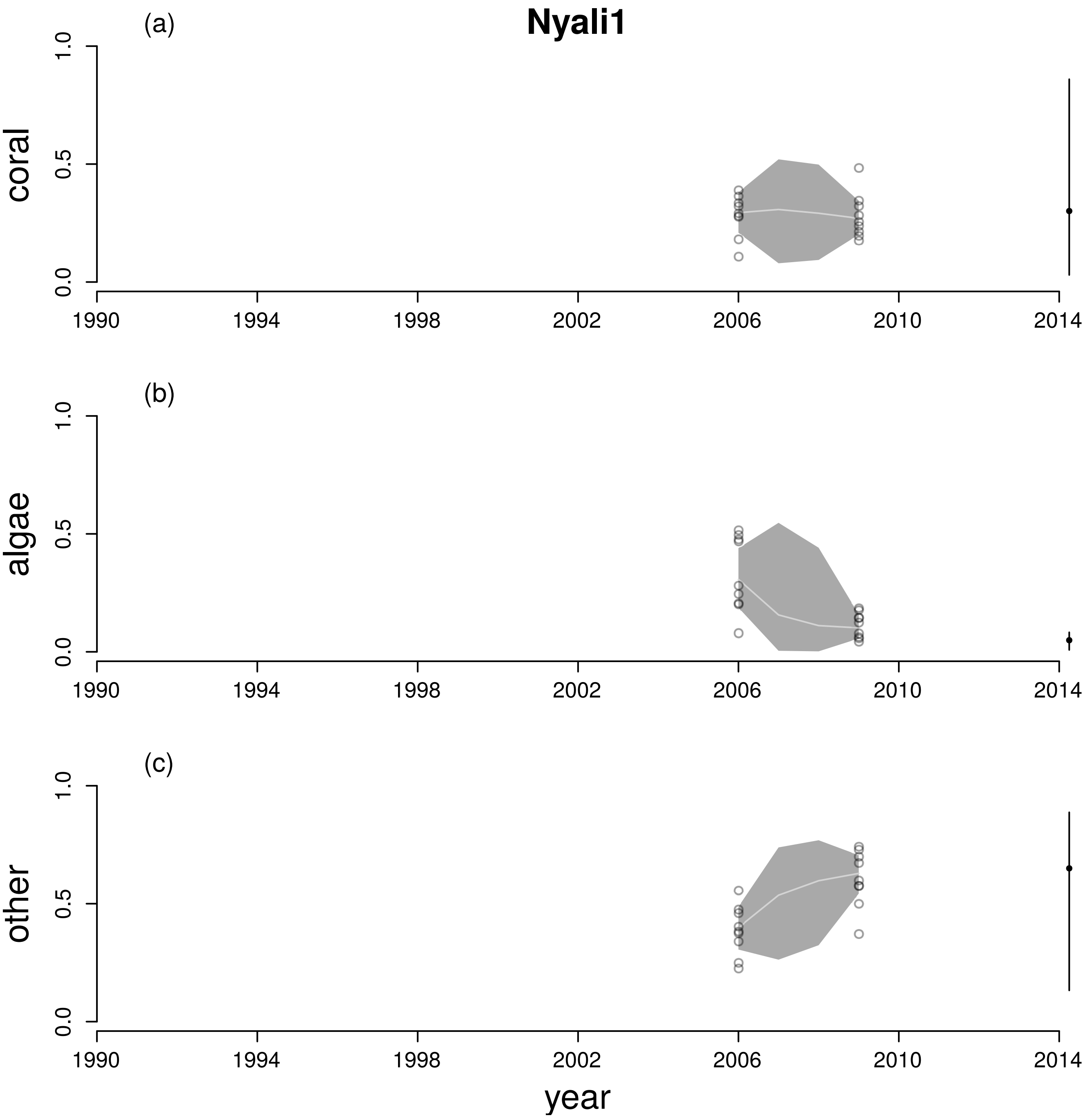

Figure A28: Time series for cover of hard corals (a), macroalgae (b) and other (c) at Nyali1. See Figure A6 legend for explanation.

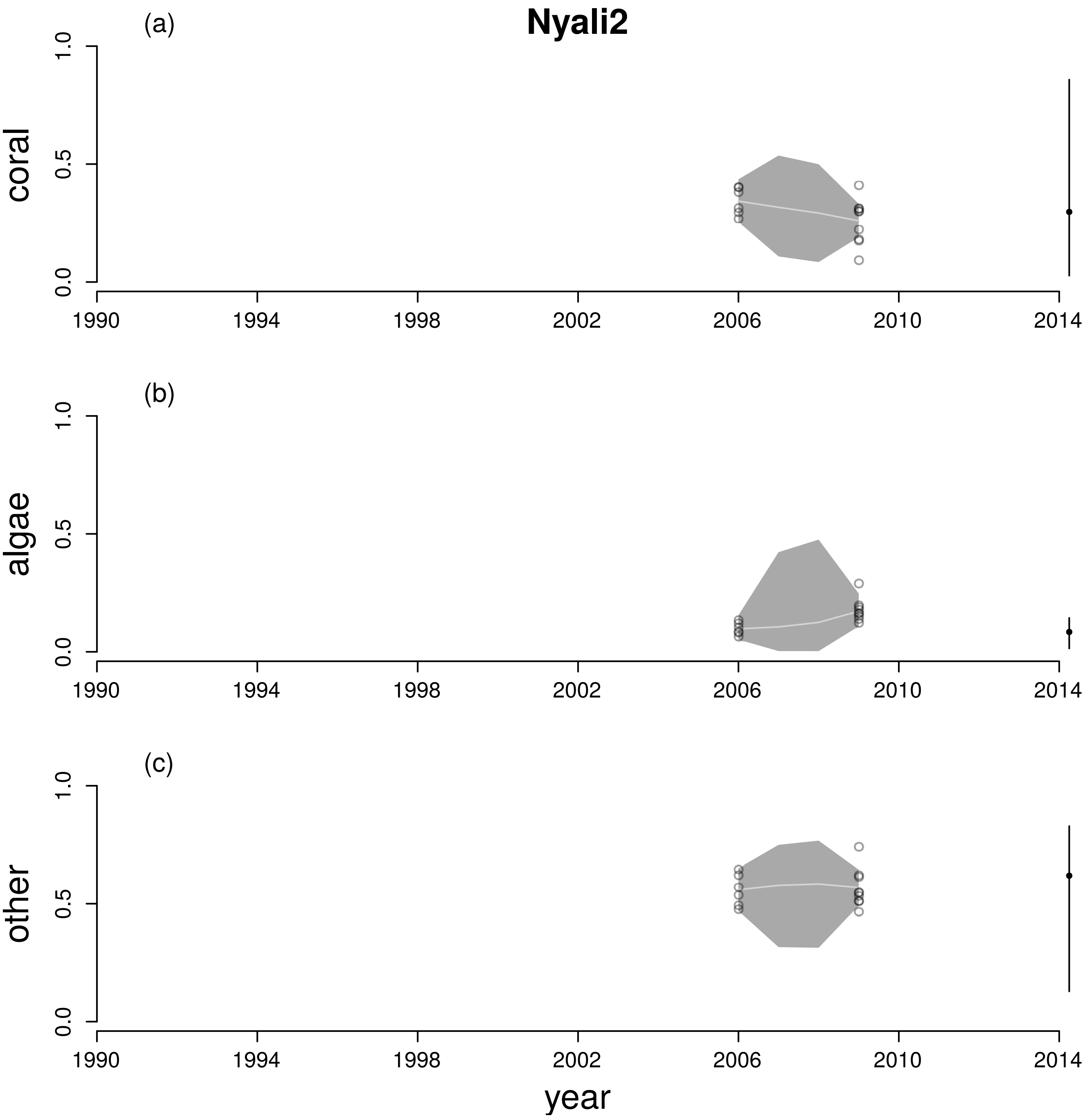

Figure A29: Time series for cover of hard corals (a), macroalgae (b) and other (c) at Nyali2. See Figure A6 legend for explanation.

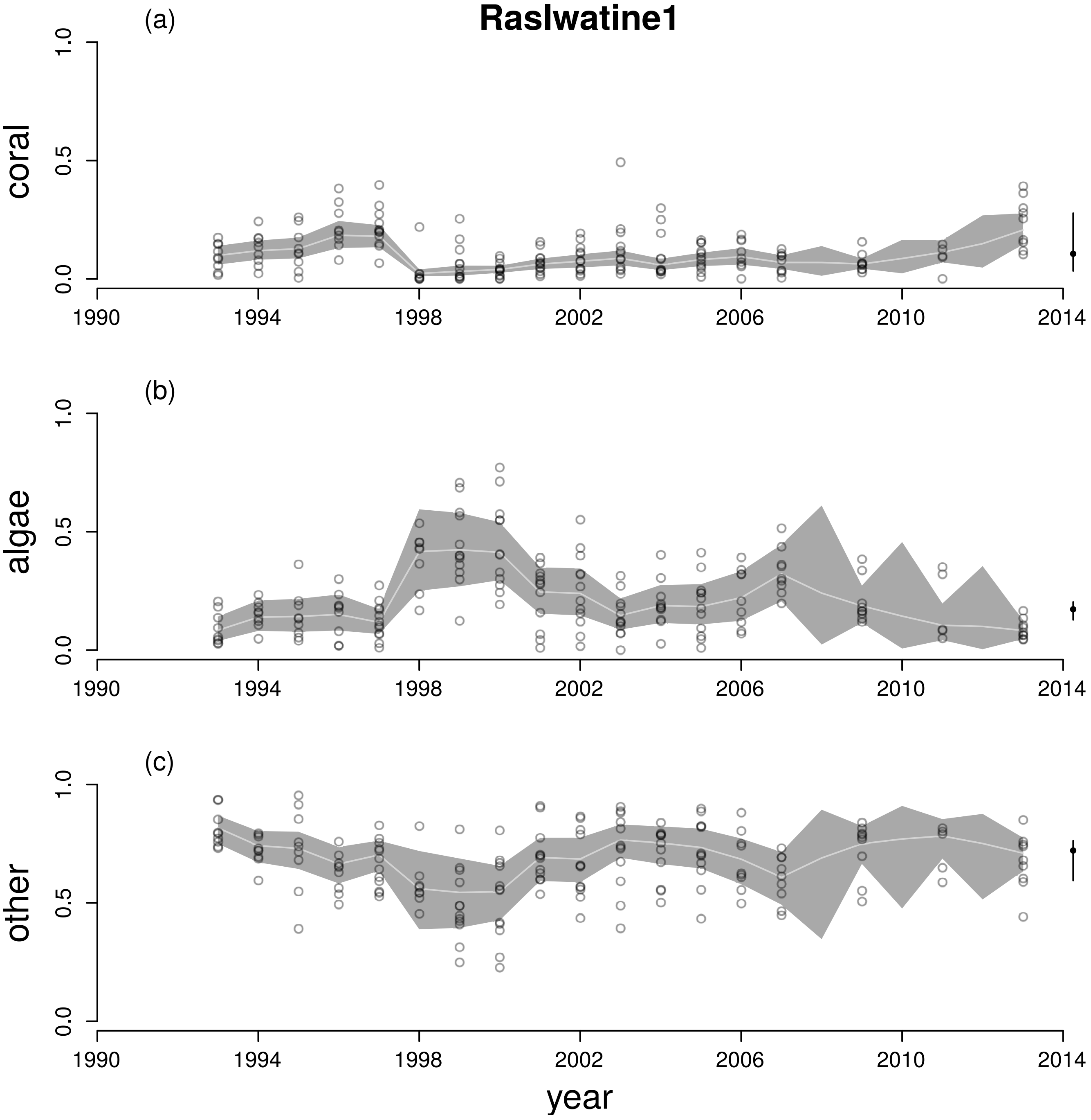

Figure A30: Time series for cover of hard corals (a), macroalgae (b) and other (c) at RasIwatine1. See Figure A6 legend for explanation.

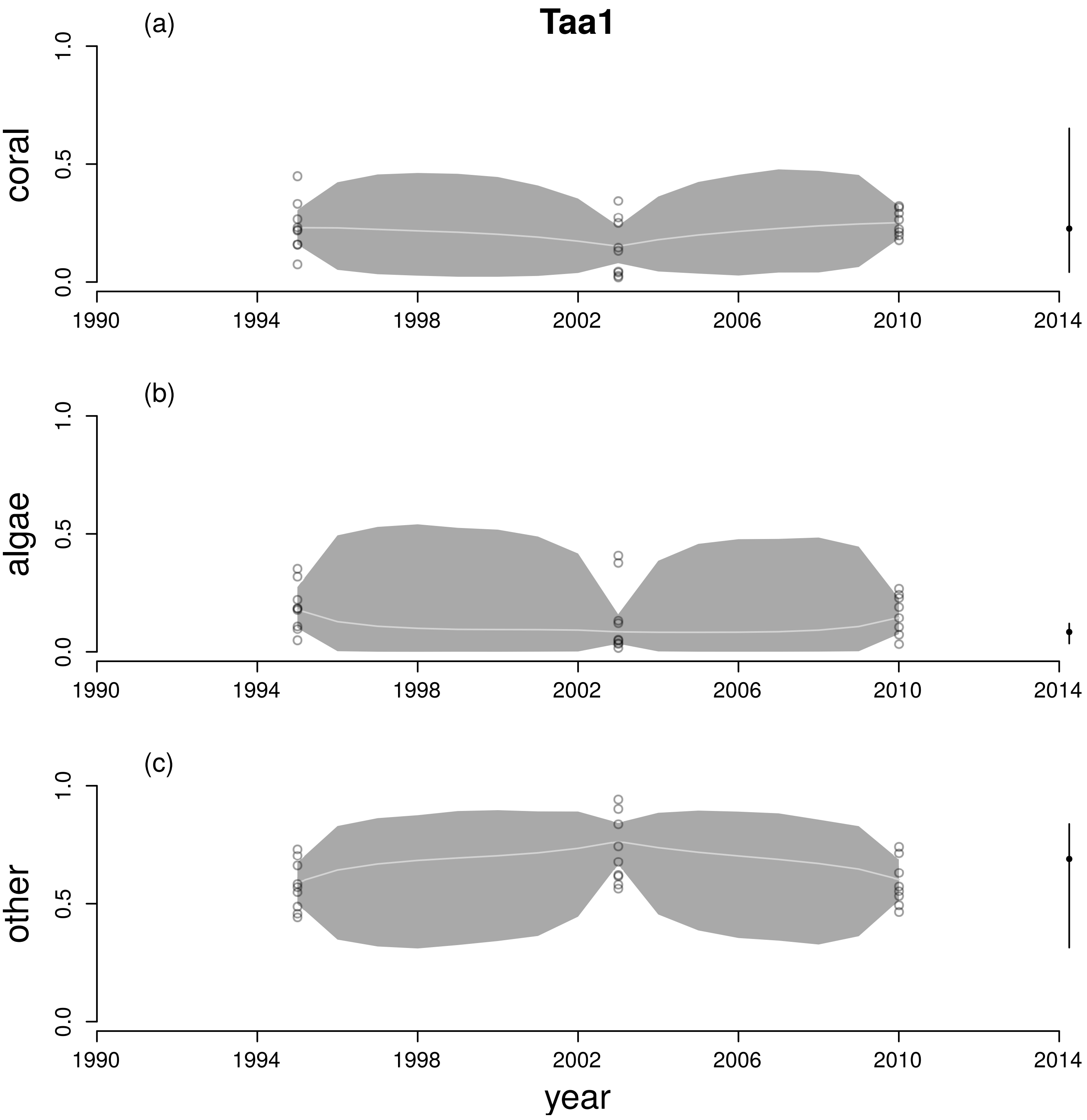

Figure A31: Time series for cover of hard corals (a), macroalgae (b) and other (c) at Taa1. See Figure A6 legend for explanation.

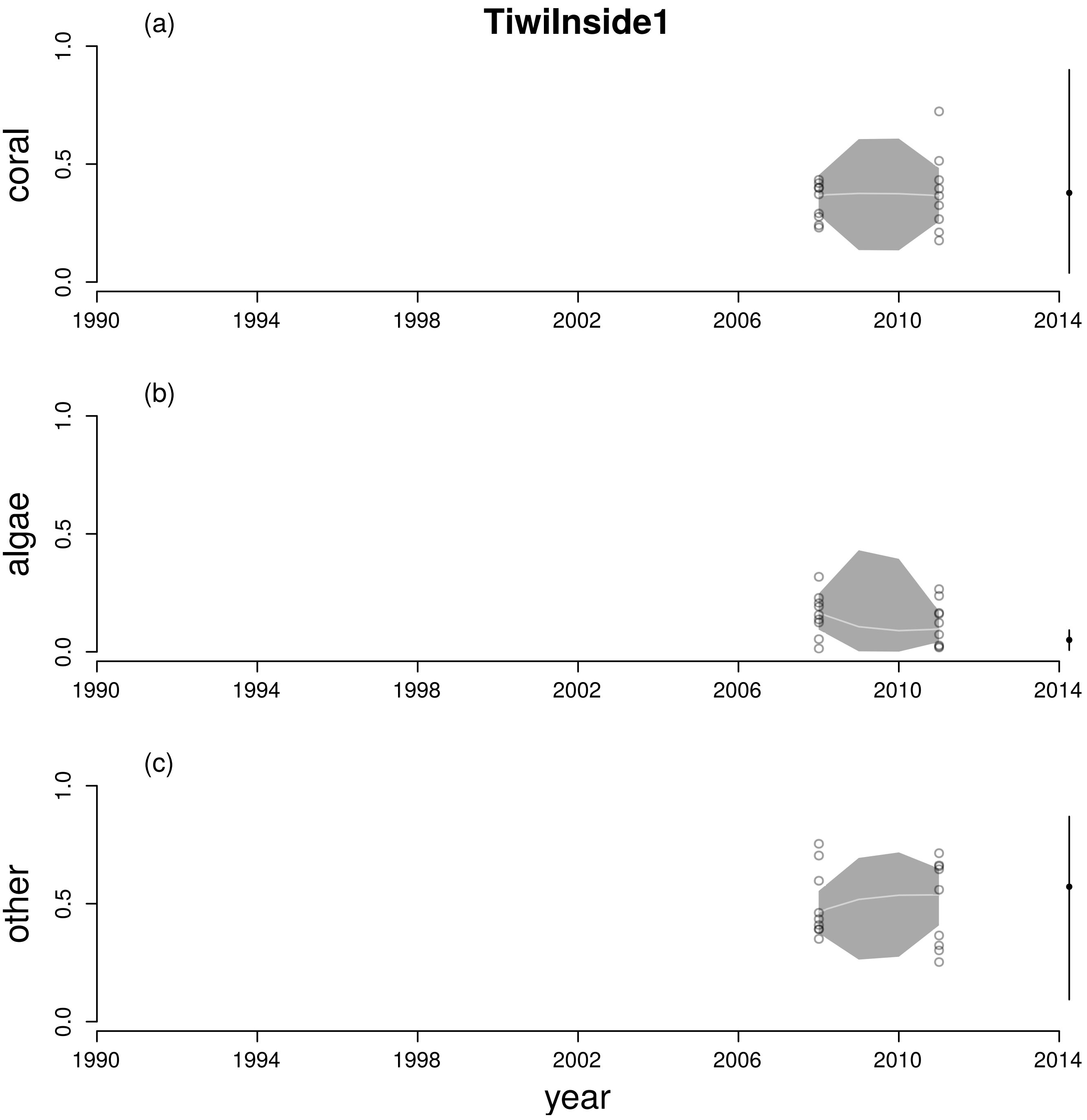

Figure A32: Time series for cover of hard corals (a), macroalgae (b) and other (c) at TiwiInside1. See Figure A6 legend for explanation.

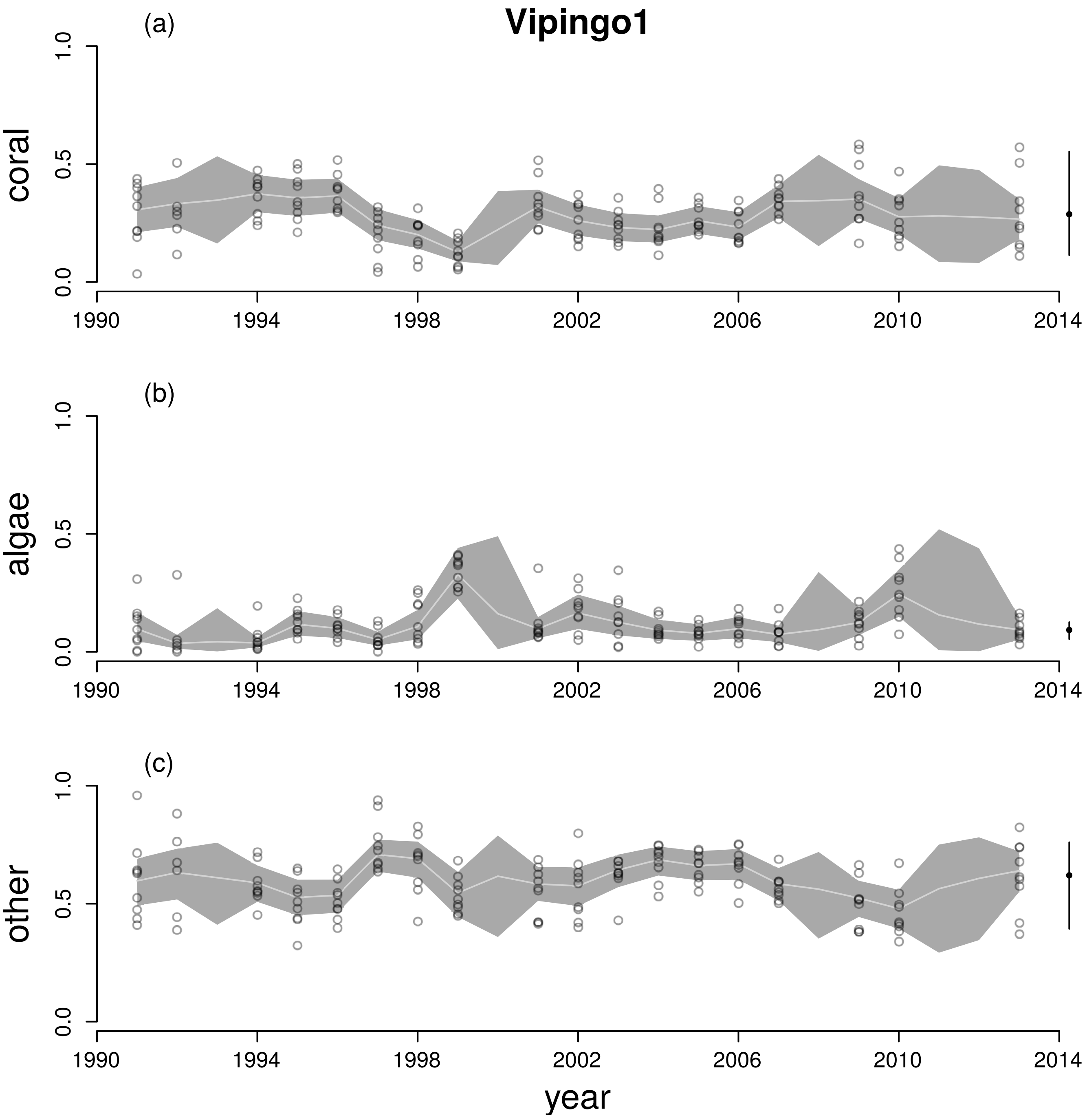

Figure A33: Time series for cover of hard corals (a), macroalgae (b) and other (c) at Vipingo1. See Figure A6 legend for explanation.

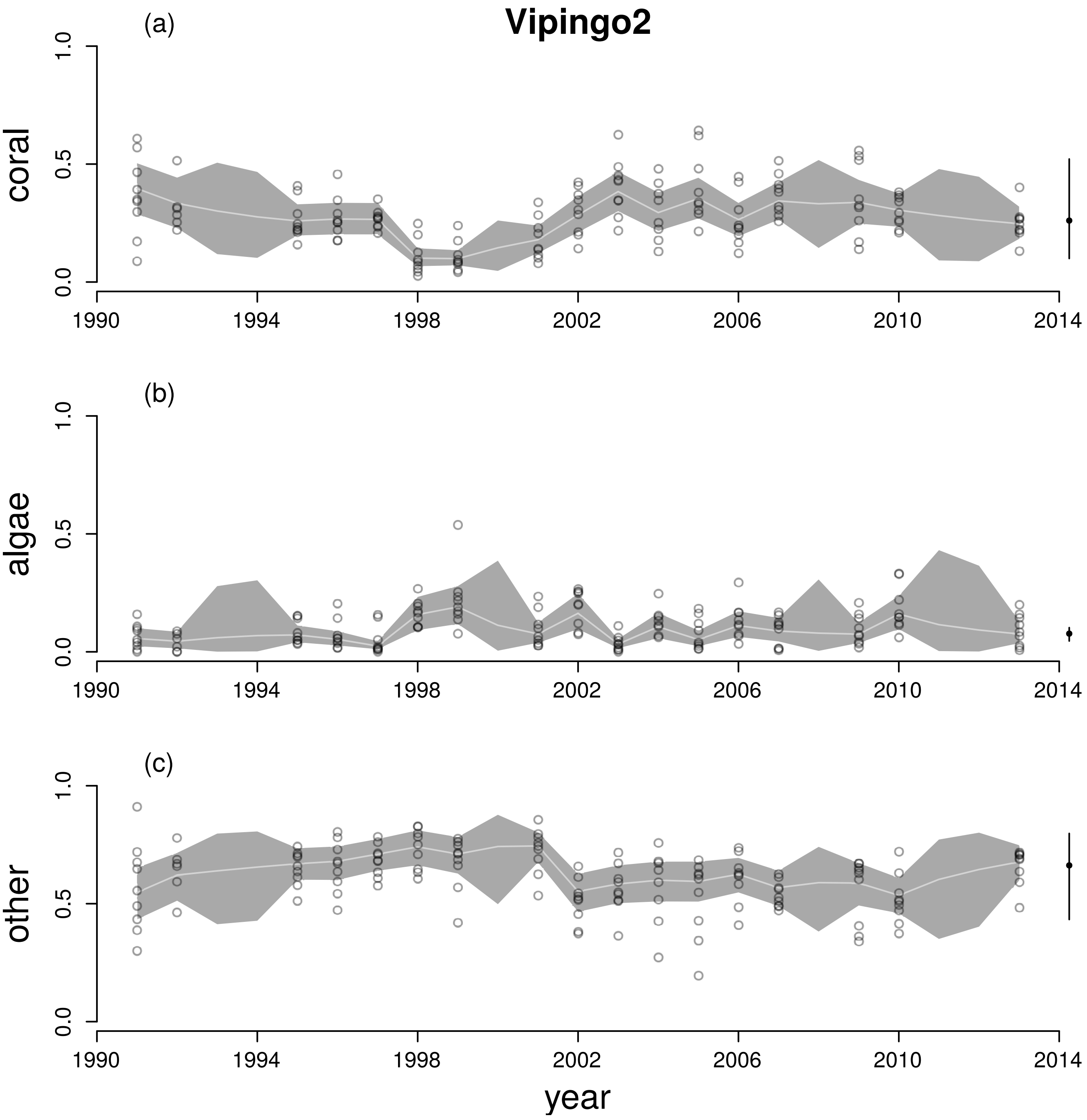

Figure A34: Time series for cover of hard corals (a), macroalgae (b) and other (c) at Vipingo2. See Figure A6 legend for explanation.

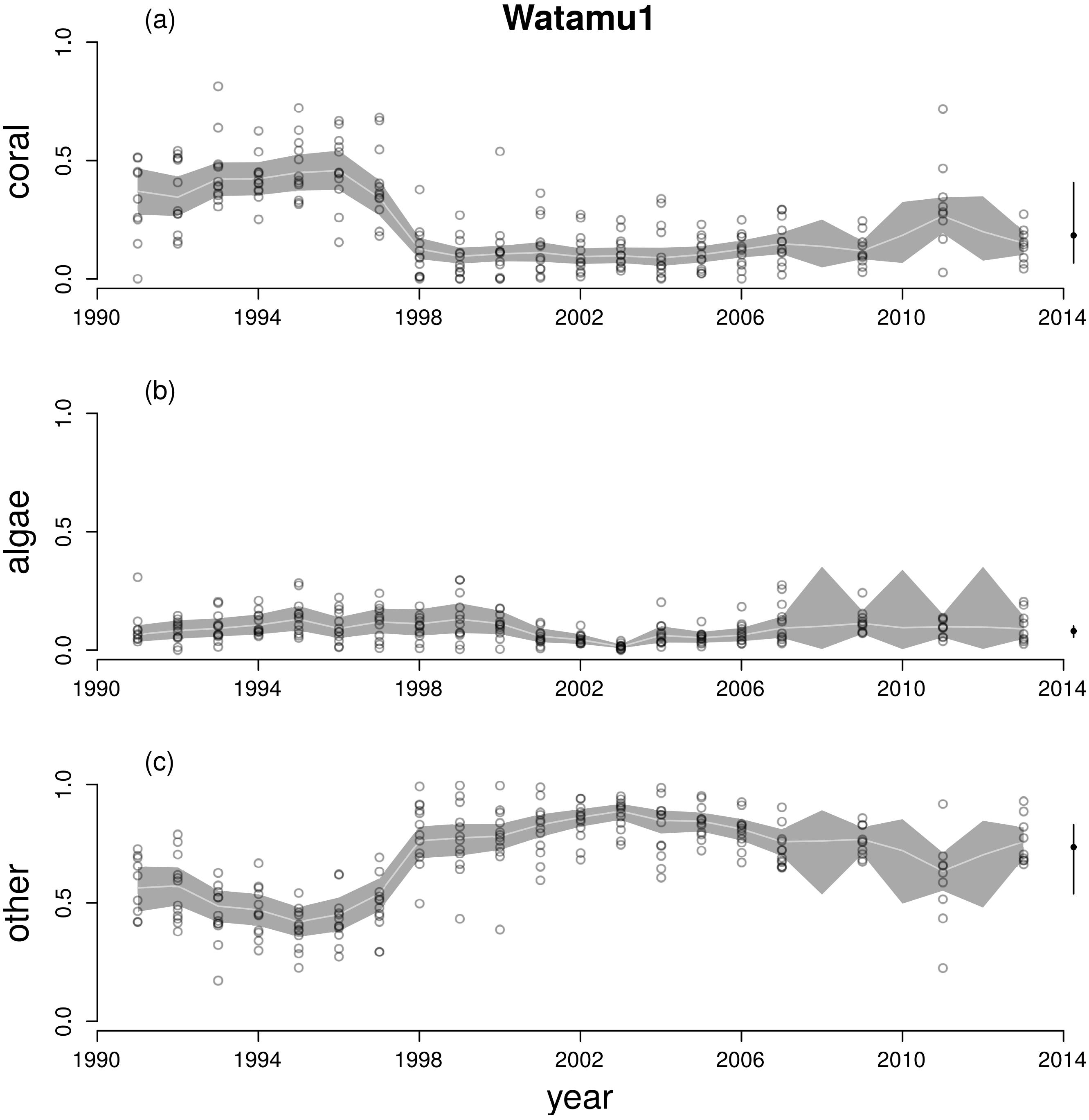

Figure A35: Time series for cover of hard corals (a), macroalgae (b) and other (c) at Watamu1. See Figure A6 legend for explanation.

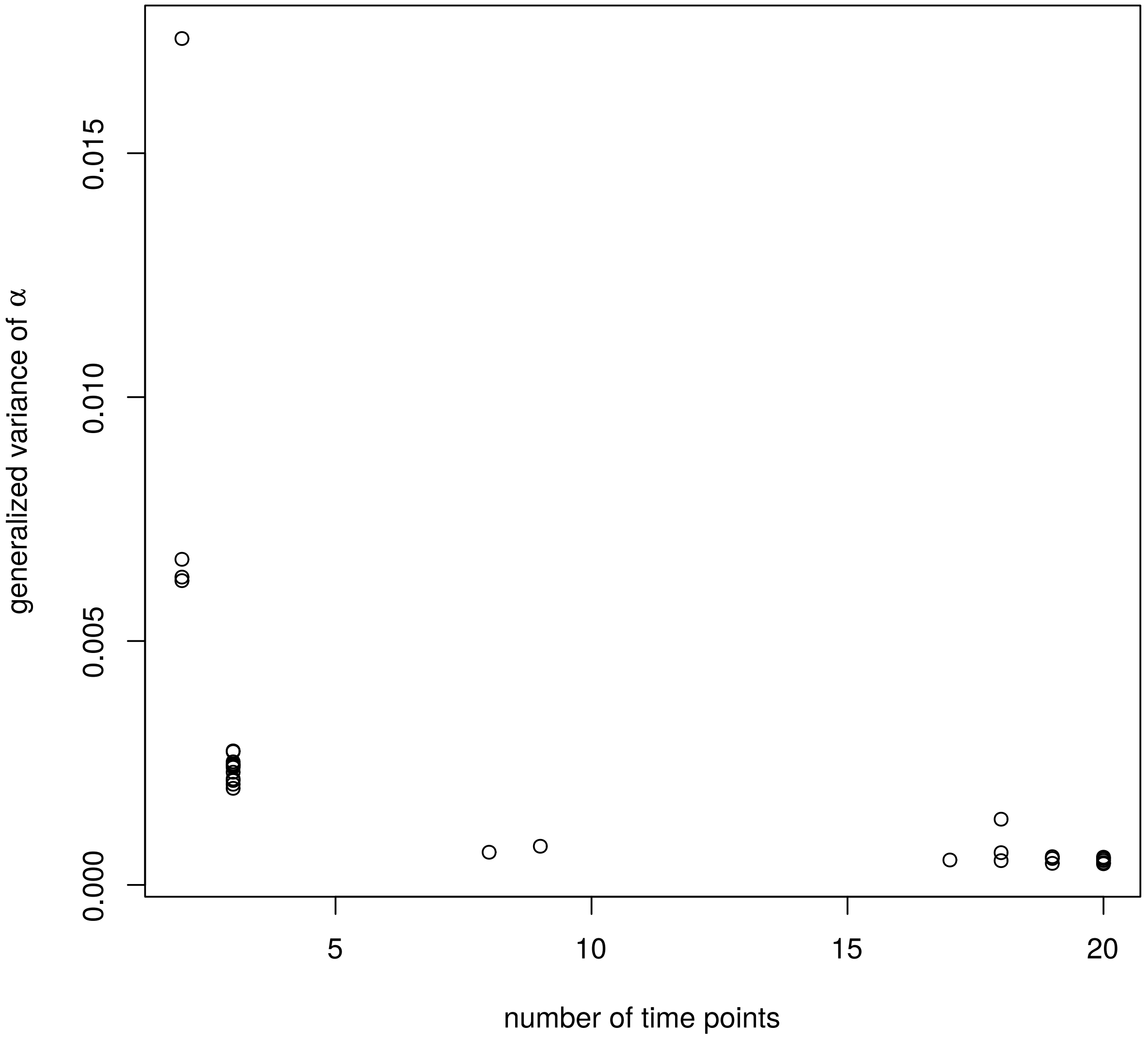

Figure A36: Relationship between sample generalized variance (determinant of sample covariance matrix over Monte Carlo iterations) of site-specific effects on dynamics $\boldsymbol{\alpha}_i$ and number of time points per site.

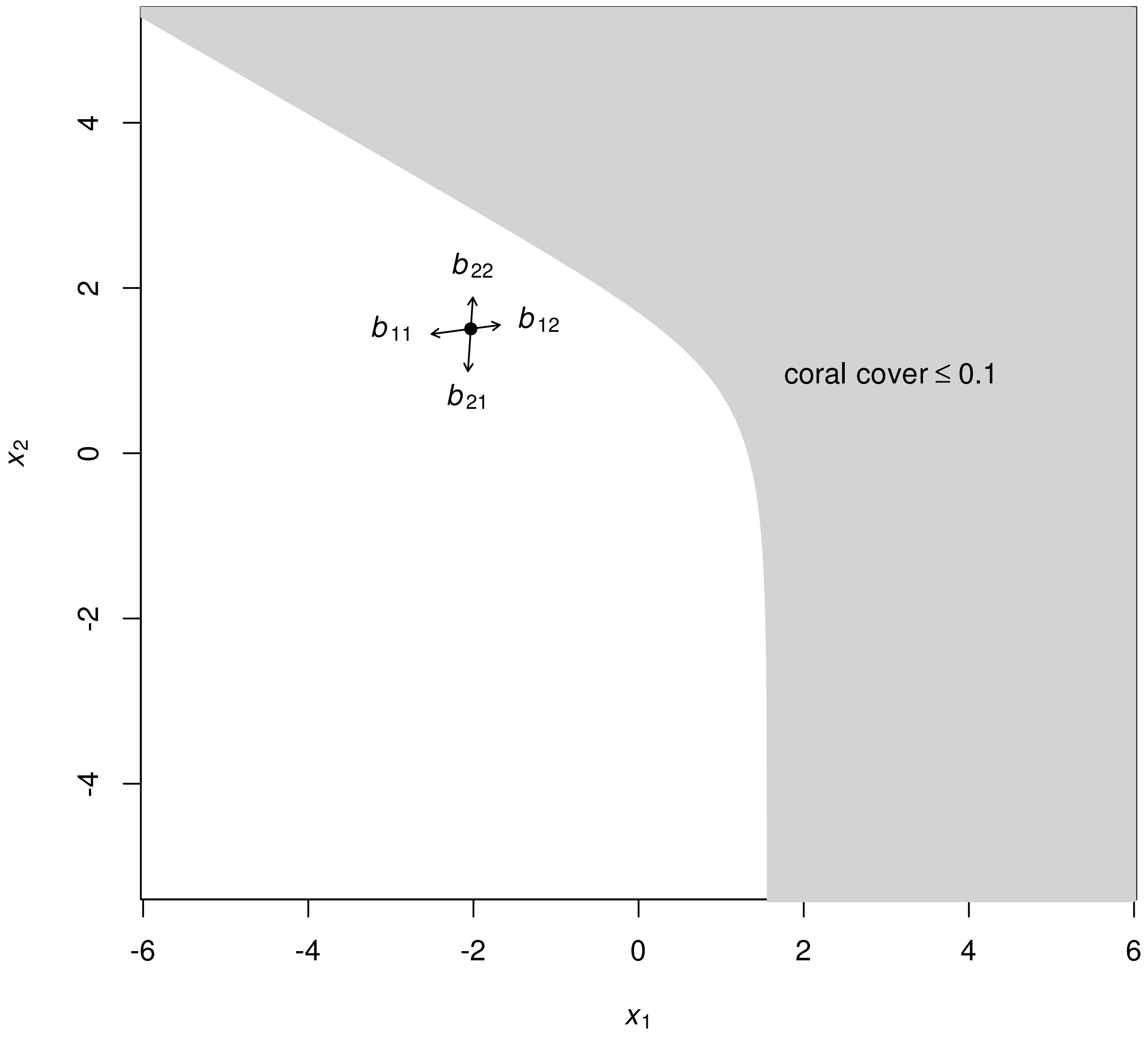

Figure A37: Effects of the elements of $\mathbf{B}$ on the location of the stationary mean $\boldsymbol{\mu}^*$. Axes: the two components of isometric logratio transformed benthic composition (Equation A.1). Component x_1 is proportional to the log of the ratio of algae to coral. Component x_2 is proportional to the log of the ratio of other to the geometric mean of algae and coral. Black dot: point estimate of stationary mean $\boldsymbol{\mu}^*$, calculated from Equation A.4 using posterior means of $\mathbf{a}$ and $\mathbf{B}$. Arrows: directions of derivatives of $\boldsymbol{\mu}^*$ with respect to each element of $\mathbf{B}$ (Equation A.12). Shaded region: coral cover ≤ 0.1.

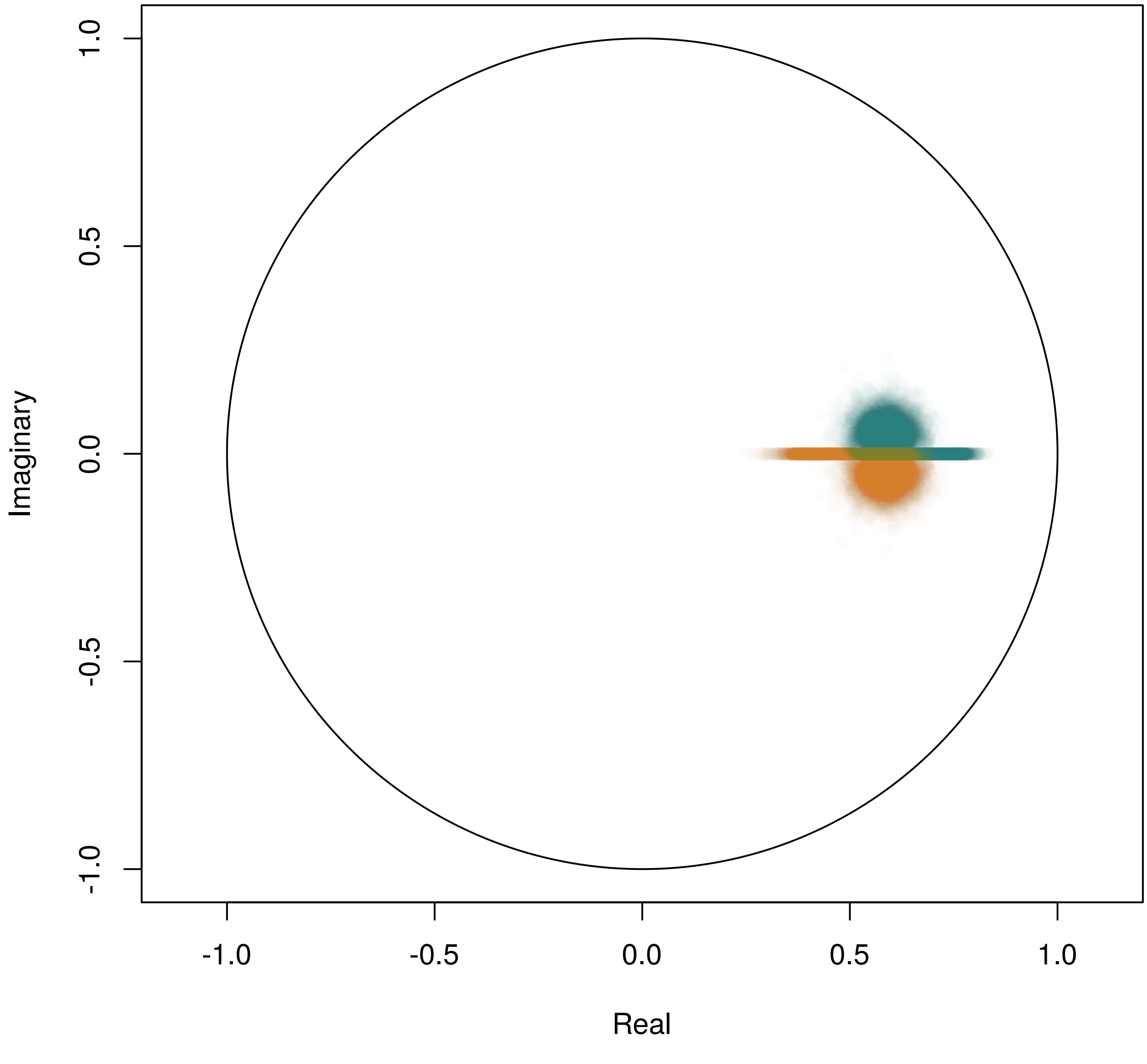

Figure A38: Distribution of the two eigenvalues of **B** in the complex plane. Each Monte Carlo sample gives a pair of eigenvalues, represented by two points: λ_1 (green), posterior mean magnitude 0.64, 95% HPD interval $(0.53, 0.75)$; λ_2 (orange), posterior mean magnitude 0.53, 95% HPD interval $(0.41, 0.66)$)

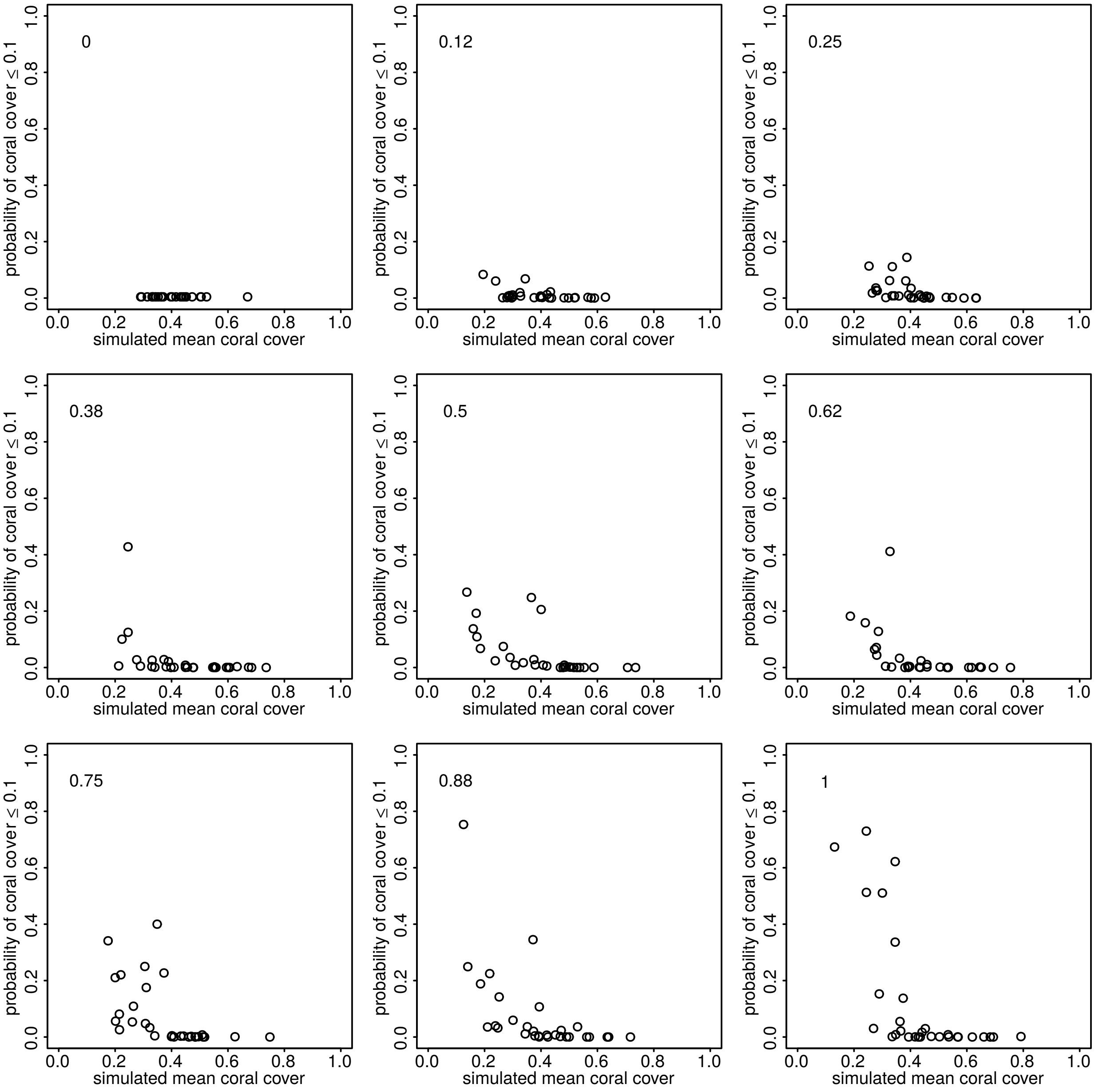

Figure A39: Effects of among-site variability on simulated relationship between long-term probability of coral cover ≤ 0.1 (y-axis) and sample mean coral cover (x-axis). Data sets simulated as described in section A9. Among-site covariance matrices were the posterior mean of $\mathbf{Z}$ from the real data, scaled by a factor $0 \leq c \leq 1$, whose value is given in the top left of each panel. Thus the amount of among-site variability increases from top left to bottom right. The axis scales are the same on all panels.

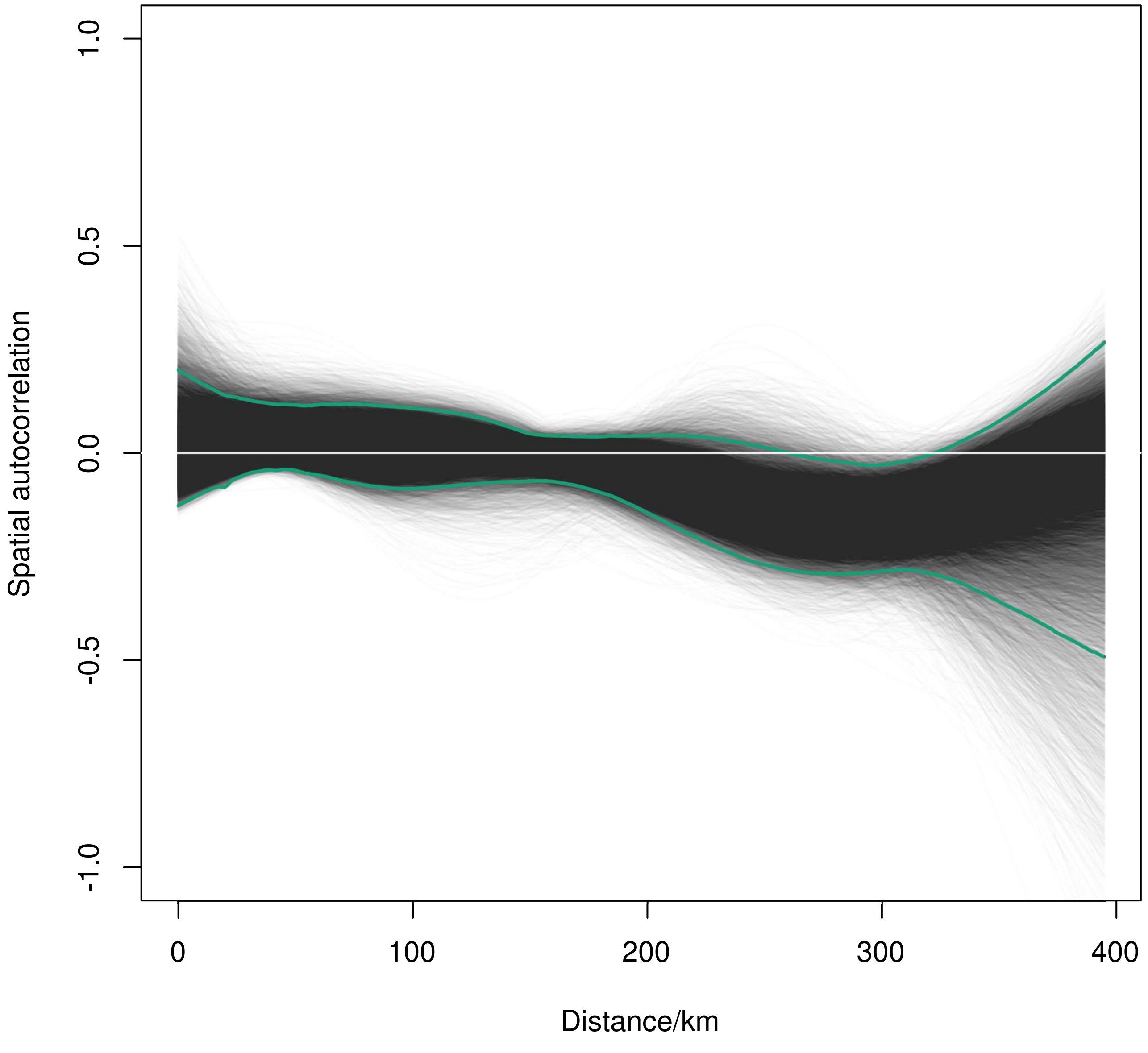

Figure A40: Spline correlogram of spatial autocorrelation in $q_{0,1,i}$. Grey lines: spline correlograms from each of 20000 Monte Carlo iterations. Thick green lines: 95% highest posterior density envelope. White horizontal line: zero-correlation reference line.

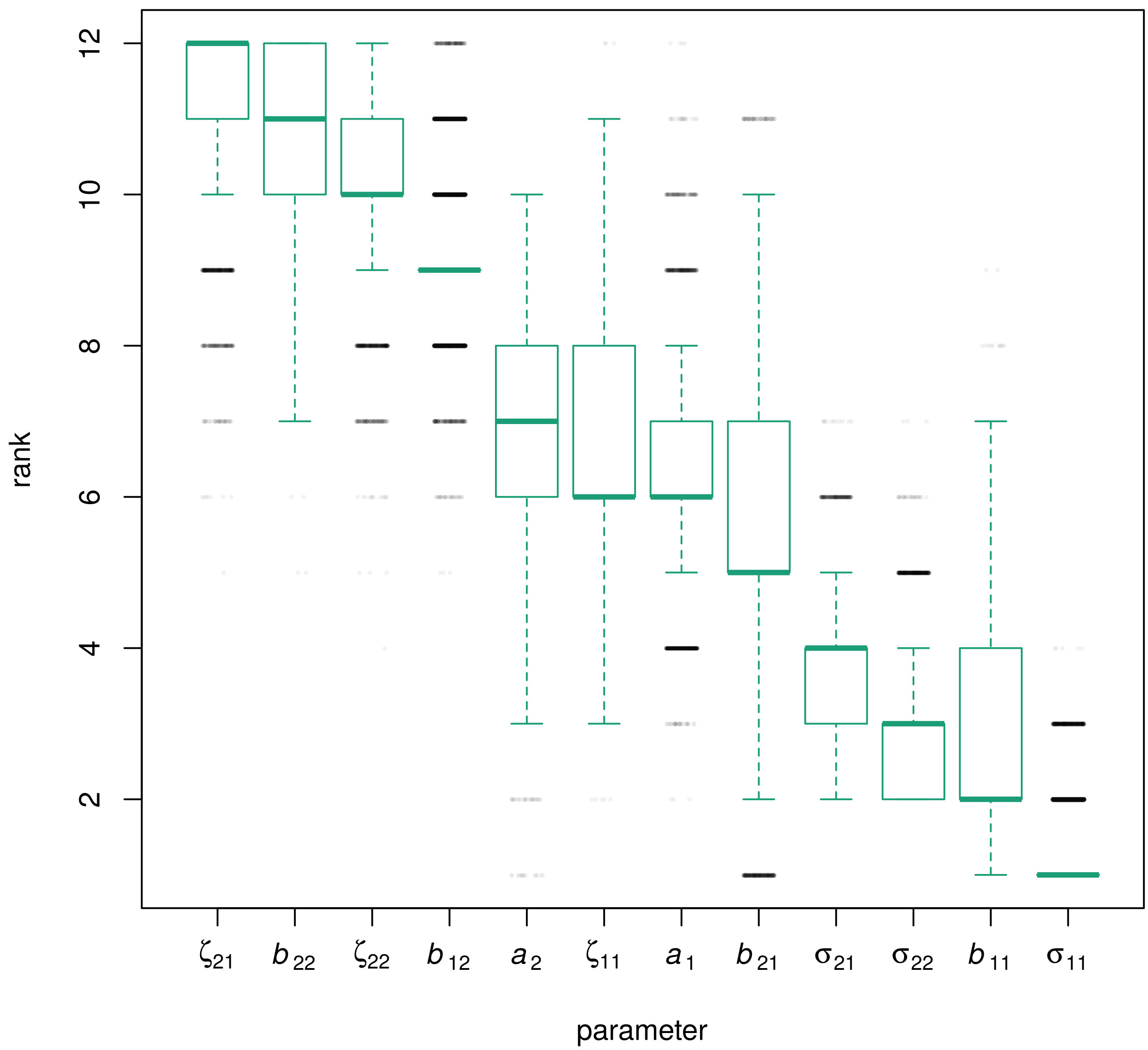

Figure A41: Ranks of partial derivatives of the long-term probability of coral cover less than or equal to 0.1 with respect to elements of the **B** matrix, the **a** vector, the covariance matrix of random temporal variation $\mathbf{\Sigma}$, and the covariance matrix of among-site variability **Z**. Parameters are ranked in descending order of median rank (higher ranks indicate larger magnitudes of partial derivative). Outliers are indicated as jittered black dots. For the covariance matrices, the elements σ_{12} and ζ_{12} are not shown, because they are constrained to be equal to σ_{21} and ζ_{21} respectively.

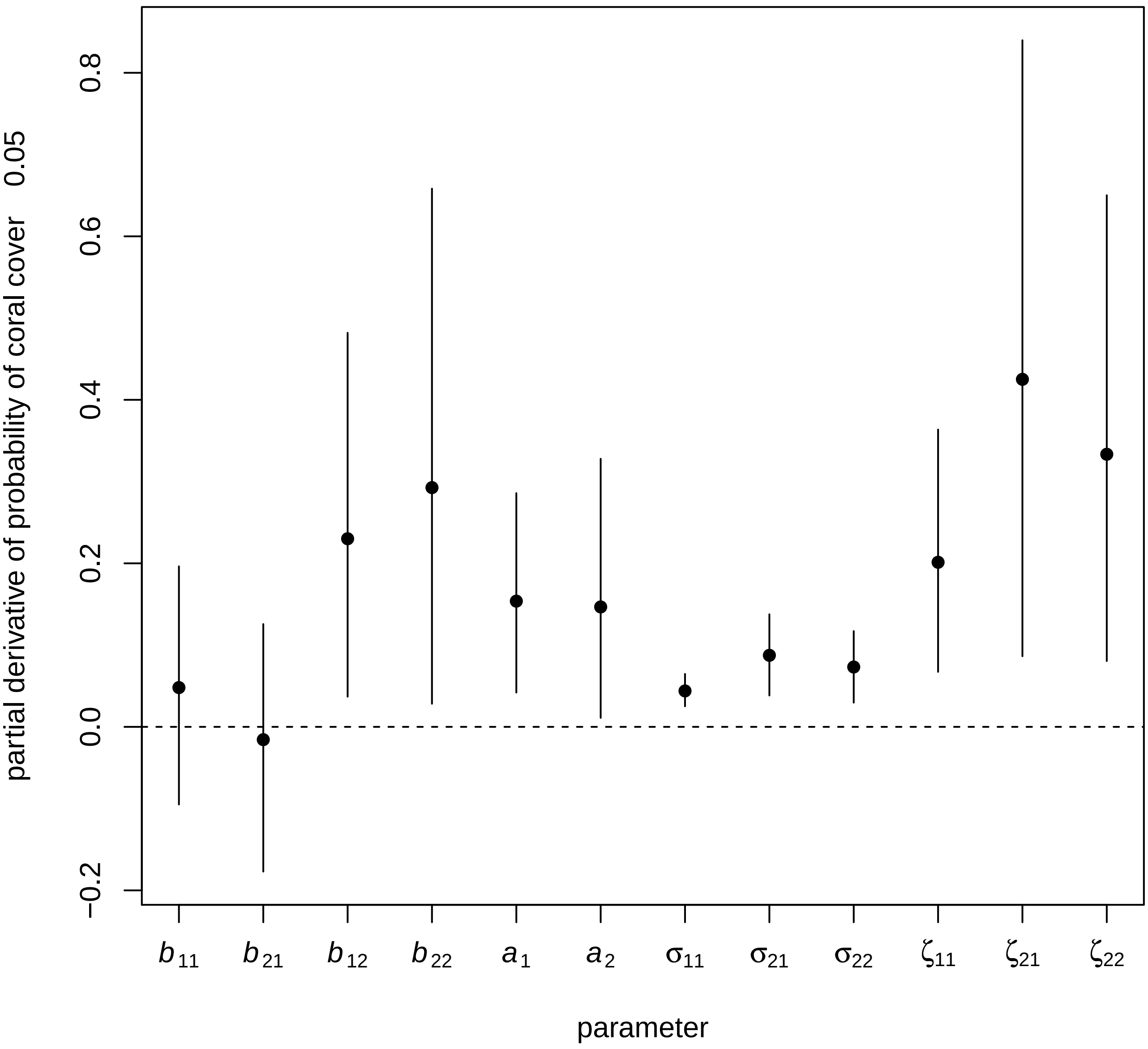

Figure A42: Elements of the gradient vector of partial derivatives of the long-term probability of coral cover less than or equal to 0.05 with respect to elements of the **B** matrix, the **a** vector, the covariance matrix of random temporal variation Σ, and the covariance matrix of among-site variability **Z**. For each parameter, the dot is the posterior mean and the bar is a 95% HPD interval. For the covariance matrices, the elements σ_{12} and ζ_{12} are not shown, because they are constrained to be equal to σ_{21} and ζ_{21} respectively.

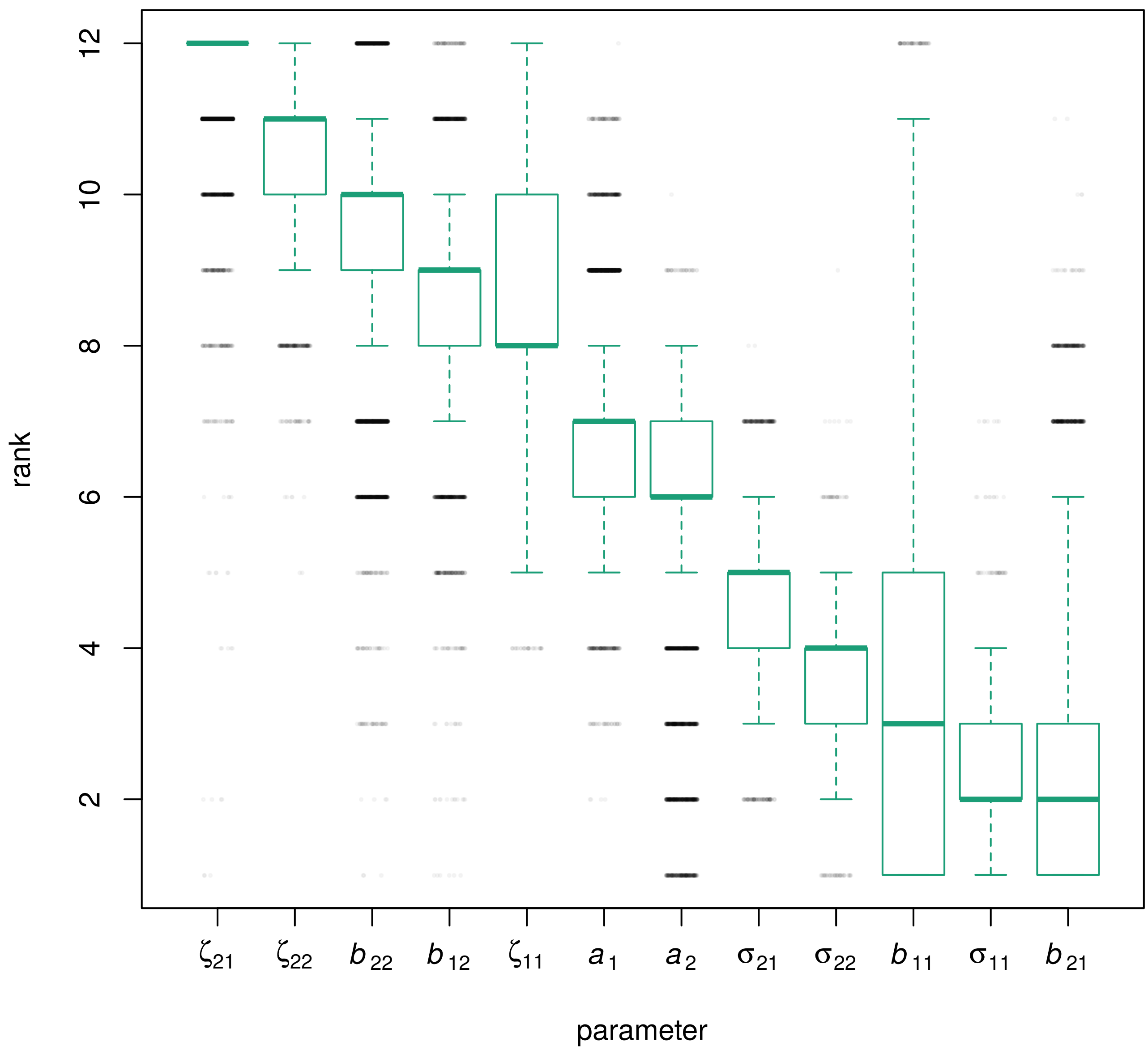

Figure A43: Ranks of partial derivatives of the long-term probability of coral cover less than or equal to 0.05 with respect to elements of the **B** matrix, the **a** vector, the covariance matrix of random temporal variation Σ, and the covariance matrix of among-site variability **Z**. Parameters are ranked in descending order of median rank (higher ranks indicate larger magnitudes of partial derivative). Outliers are indicated as jittered black dots. For the covariance matrices, the elements σ_{12} and ζ_{12} are not shown, because they are constrained to be equal to σ_{21} and ζ_{21} respectively.

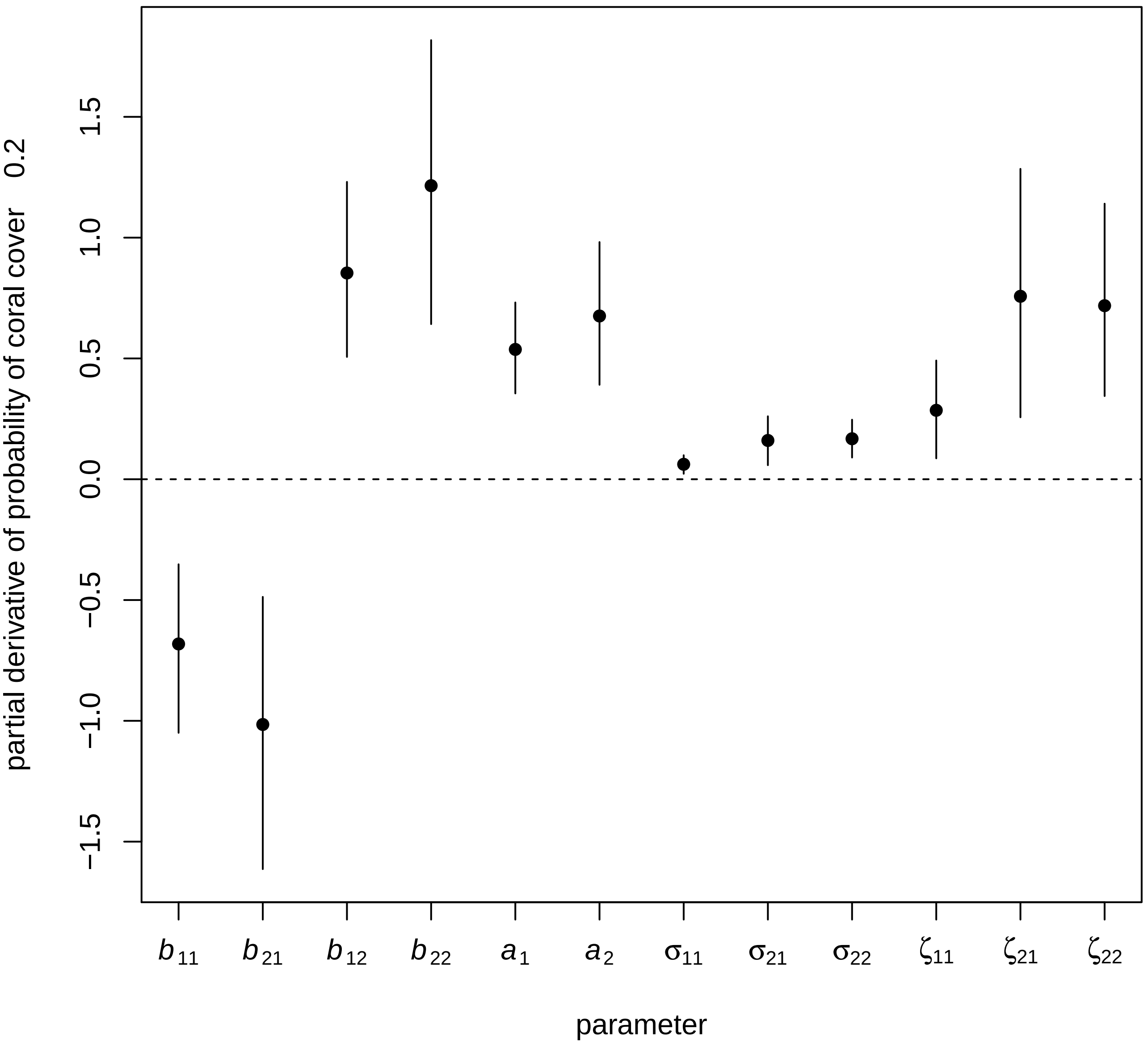

Figure A44: Elements of the gradient vector of partial derivatives of the long-term probability of coral cover less than or equal to 0.2 with respect to elements of the **B** matrix, the **a** vector, the covariance matrix of random temporal variation Σ, and the covariance matrix of among-site variability **Z**. For each parameter, the dot is the posterior mean and the bar is a 95% HPD interval. For the covariance matrices, the elements σ_{12} and ζ_{12} are not shown, because they are constrained to be equal to σ_{21} and ζ_{21} respectively.

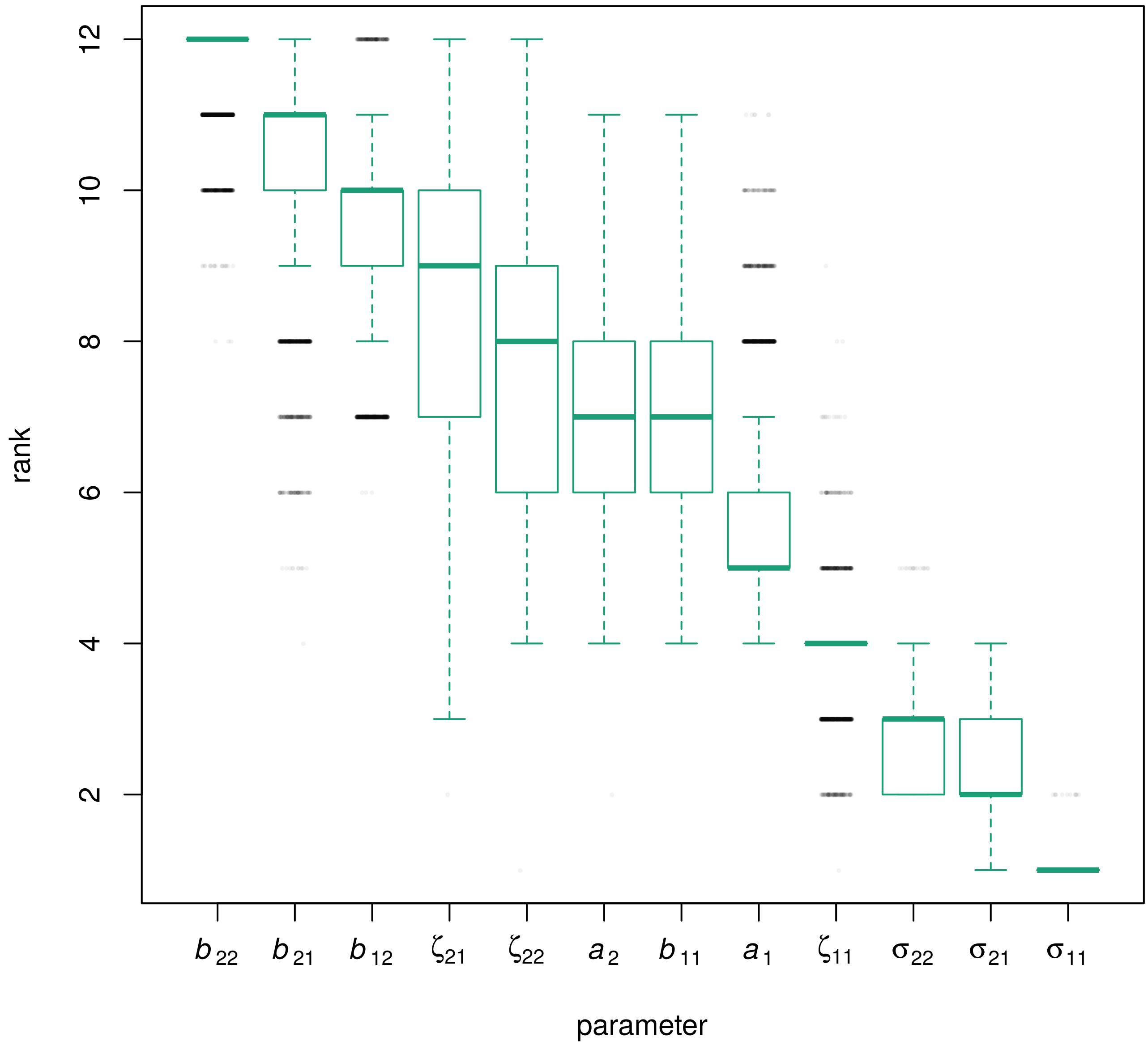

Figure A45: Ranks of partial derivatives of the long-term probability of coral cover less than or equal to 0.2 with respect to elements of the **B** matrix, the **a** vector, the covariance matrix of random temporal variation Σ, and the covariance matrix of among-site variability **Z**. Parameters are ranked in descending order of median rank (higher ranks indicate larger magnitudes of partial derivative). Outliers are indicated as jittered black dots. For the covariance matrices, the elements σ_{12} and ζ_{12} are not shown, because they are constrained to be equal to σ_{21} and ζ_{21} respectively.

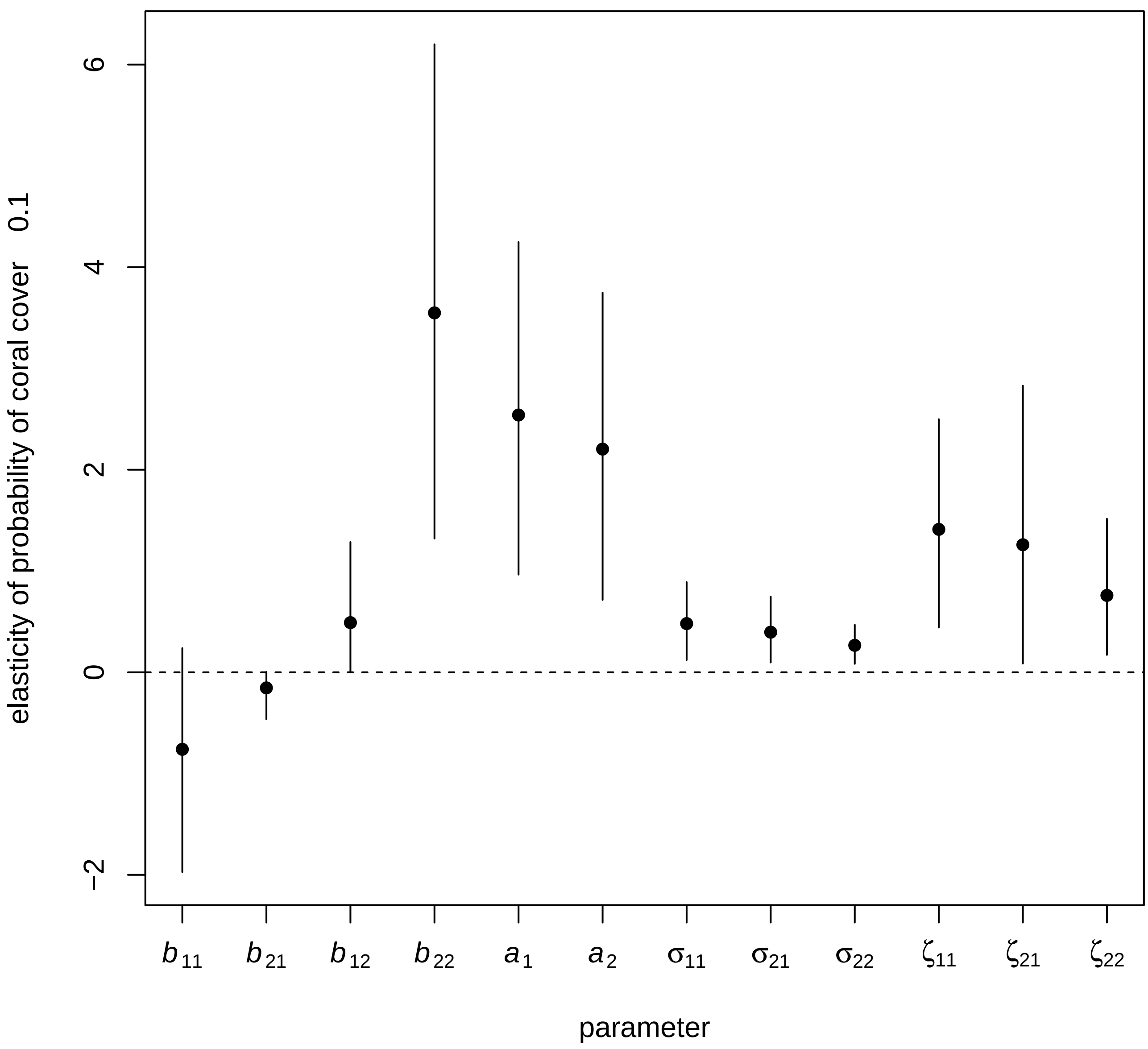

Figure A46: Elasticities of the long-term probability of coral cover less than or equal to 0.1 with respect to elements of the **B** matrix, the **a** vector, the covariance matrix of random temporal variation Σ, and the covariance matrix of among-site variability **Z**. For each parameter, the dot is the posterior mean and the bar is a 95% HPD interval. For the covariance matrices, the elements σ_{12} and ζ_{12} are not shown, because they are constrained to be equal to σ_{21} and ζ_{21} respectively.

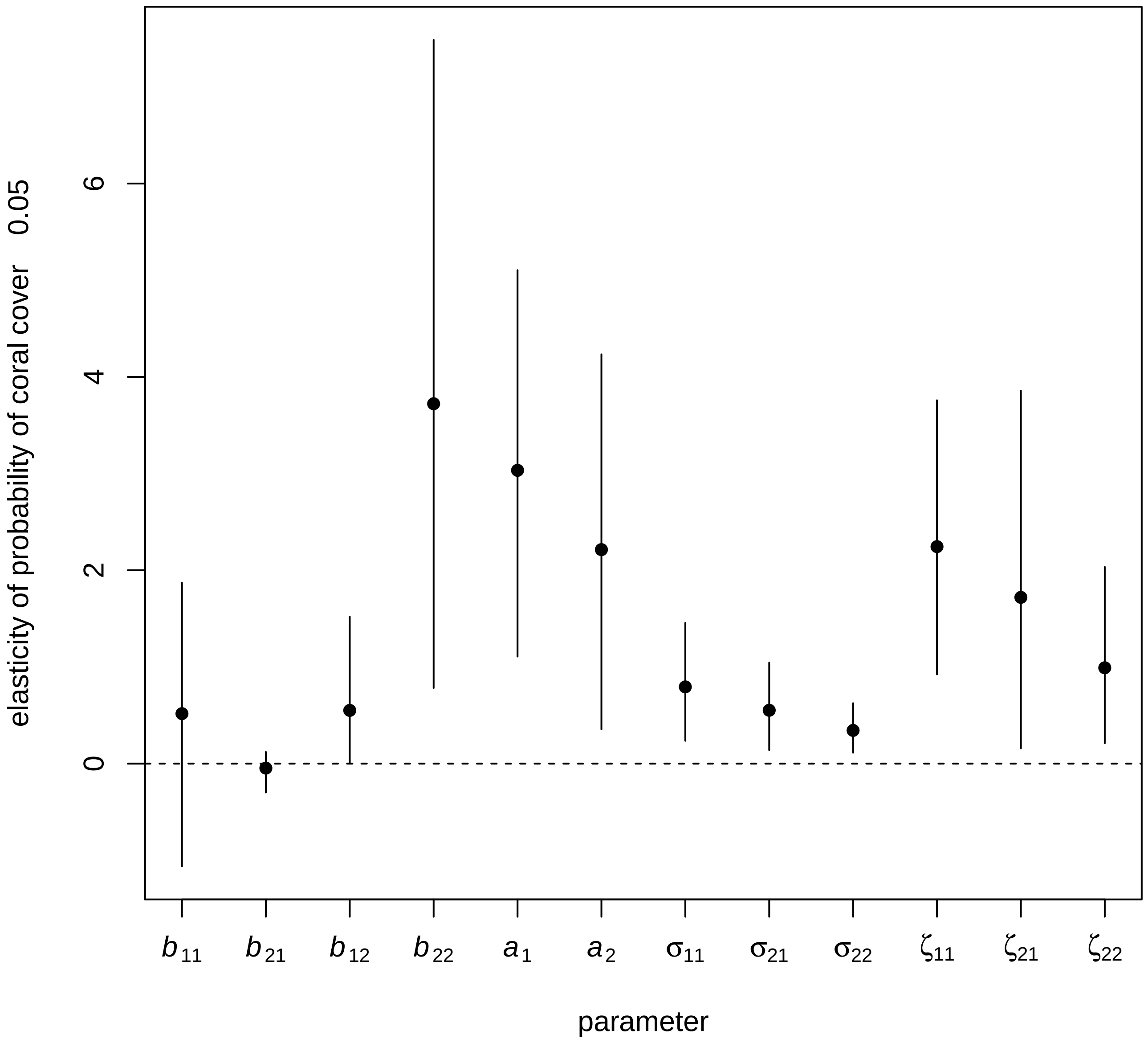

Figure A47: Elasticities of the long-term probability of coral cover less than or equal to 0.05 with respect to elements of the **B** matrix, the **a** vector, the covariance matrix of random temporal variation $\mathbf{\Sigma}$, and the covariance matrix of among-site variability **Z**. For each parameter, the dot is the posterior mean and the bar is a 95% HPD interval. For the covariance matrices, the elements σ_{12} and ζ_{12} are not shown, because they are constrained to be equal to σ_{21} and ζ_{21} respectively.

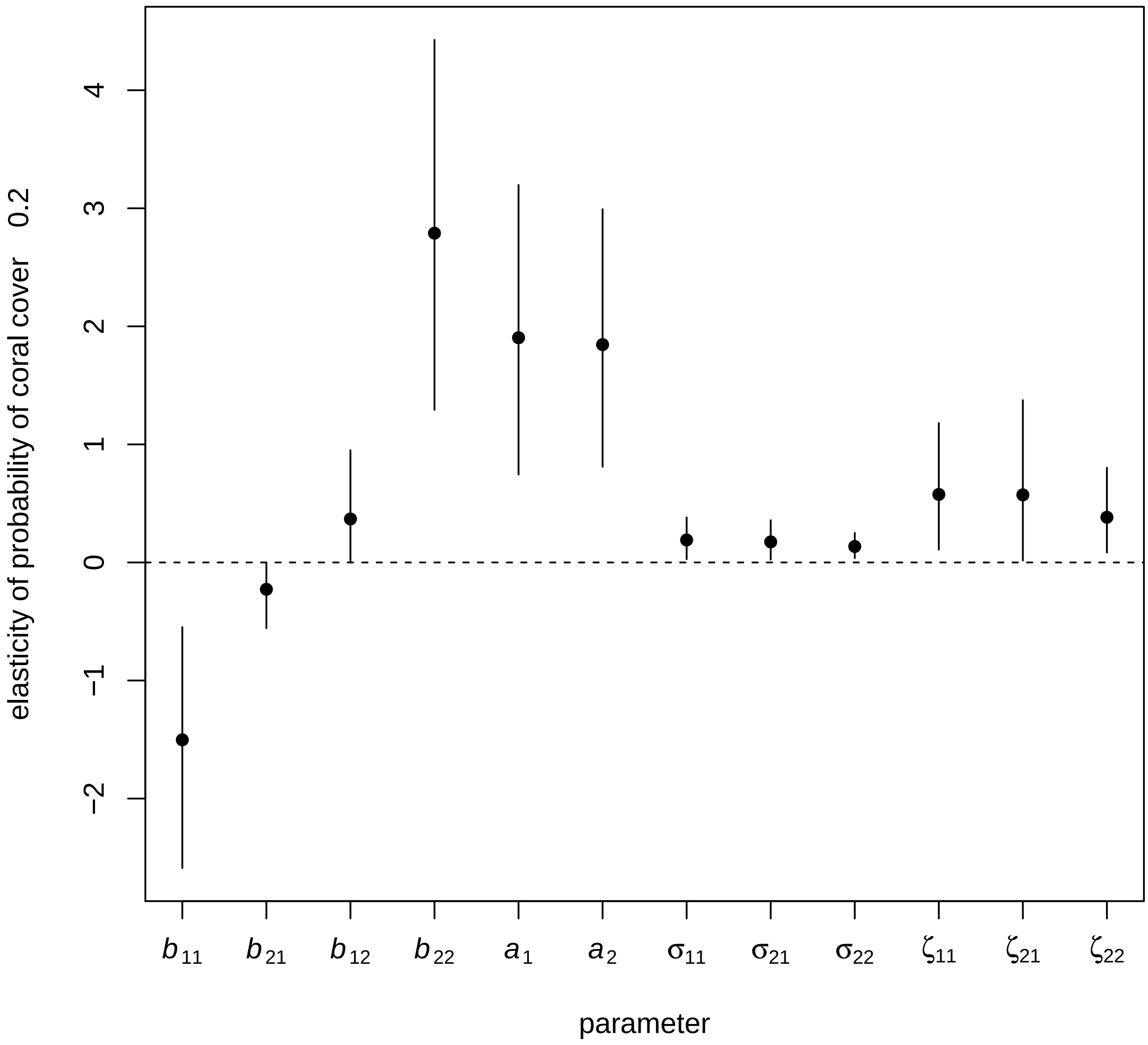

Figure A48: Elasticities of the long-term probability of coral cover less than or equal to 0.2 with respect to elements of the **B** matrix, the **a** vector, the covariance matrix of random temporal variation **Σ**, and the covariance matrix of among-site variability **Z**. For each parameter, the dot is the posterior mean and the bar is a 95% HPD interval. For the covariance matrices, the elements σ_{12} and ζ_{12} are not shown, because they are constrained to be equal to σ_{21} and ζ_{21} respectively.
